# Acquired temozolomide resistance in MGMT^low^ gliomas is associated with regulation of homologous recombination repair by ROCK2

**DOI:** 10.1038/s41419-022-04590-6

**Published:** 2022-02-10

**Authors:** Xin Zhang, Tao Li, Mengdi Yang, Qianming Du, Rui Wang, Bin Fu, Yingying Tan, Mengran Cao, Yaxin Chen, Qing Wang, Rong Hu

**Affiliations:** 1 State Key Laboratory of Natural Medicines, School of Life Science and Technology, China Pharmaceutical University, Nanjing, China; 2 State Key Laboratory of Natural Medicines, School of Basic Medicine and Clinical Pharmacy, China Pharmaceutical University, Nanjing, China; 3 General Clinical Research Center, Nanjing First Hospital, Nanjing Medical University, Nanjing, China; 4 General Clinical Research Center, Nanjing First Hospital, China Pharmaceutical University, Nanjing, China; 5 Department of Neurosurgery, Wuxi Second Hospital Affiliated Nanjing Medical University, Wuxi, Jiangsu China

**Keywords:** Cancer therapeutic resistance, CNS cancer, Oncogenes

## Abstract

It was reported that MGMT^low^ gliomas may still be resistant to TMZ, while the mechanisms remain poorly understood. In this study, we demonstrated that rho-associated kinase 2 (ROCK2), a cytoskeleton regulator, was highly expressed in MGMT^low^ recurrent gliomas, and its expression strongly correlated with poor overall survival (OS) time in a subset of MGMT^low^ recurrent gliomas patients with TMZ therapy. And we also found that overactive ROCK2 enhanced homologous recombination repair (HR) in TMZ-resistant (TMZ-R) glioma cell lines with low MGMT expression. Silencing ROCK2 impaired HR repair, and induced double-strand break (DSB) and eradicated TMZ-R glioma cells in culture. Notably, in MGMT^low^ TMZ-R models, as a key factor of HR, ataxia telangiectasia-mutated (ATM) expression was upregulated directly by hyper-activation of ROCK2 to improve HR efficiency. ROCK2 enhanced the binding of transcription factor zinc finger E-box binding homeobox 1 (ZEB1) to ATM promoter for increasing ATM expression. Moreover, ROCK2 transformed ZEB1 into a gene activator via Yes-associated protein 1 (YAP1). These results provide evidence for the use of ROCK inhibitors in the clinical therapy for MGMT^low^ TMZ-resistant glioma. Our study also offered novel insights for improving therapeutic management of MGMT^low^ gliomas.

## Introduction

Resistance to temozolomide (TMZ) therapy is a major cause of glioma treatment failure, and therefore overcoming its resistance is critical to improving treatment outcomes. The demethylating enzyme O6-methylguanine-DNA methyltransferase (MGMT) has been implicated in intrinsic TMZ-resistance (TMZ-R) and recurrence by removing alkyl groups from the O^6^ position of guanine directly [1]. However, epigenetic silencing of MGMT is common in gliomas [2, 3], and gliomas with low MGMT levels (deficiency and low-expression) are still sufficient to confer TMZ-R [4], suggesting the existence of an MGMT-independent mechanism of acquired TMZ-R.

With low MGMT level, TMZ-R gliomas exhibited a gene mutation [5]. For instance, recurrent gliomas exhibited transcriptional silencing of the MGMT gene, followed by dysfunction of the mismatch repair (MMR) system [6] and hyperfunction of the DNA repair system [7]. With deficiency of MMR, DNA damage repair (DDR) systems were activated, leading to TMZ-R [8, 9]. Therefore, the functional availability of the DDR system presumably regulates the response of recurrent gliomas to TMZ. Hence, we hypothesize that DDR signaling is enhanced in TMZ-R glioma cells, which contributes to their phenotypic resistance.

Our previous studies showed that Rho-associated kinases 2 (ROCK2) was hyper-activated in TMZ-R models and inhibition of ROCK2 reversed TMZ-R with an increase in TMZ sensitivity [10], suggesting an association between ROCK2 and TMZ-R, but the mechanism remains elusive. Accumulating evidence suggests that ROCKs play an important role in mediating resistance to chemotherapeutics. Depletion of ROCKs enhanced the efficacy of enzalutamide in enzalutamide-resistant prostatic carcinoma cells [11]. The ROCK1 axis was reported to overcome cisplatin-related resistance [12]. A clinically administered ROCKs inhibitor fasudil significantly suppressed the growth and tumorigenicity of chemo resistant osteosarcoma cells [13]. ROCKs inhibitors increased the gemcitabine sensitivity in pancreatic cancer stem cells [14]. A previous study in our laboratory also demonstrated that ROCK2 facilitated gemcitabine-resistant pancreatic cancer cells to repair DNA damage. Interestingly, ROCKs-myosin II ablation specifically killed resistant cells via unresolved DNA damage [15]. Recent research also highlighted the involvement of RhoC-ROCK2 signaling in DNA repair in cervical cancer cells [16]. Herein, we aimed to elucidate whether ROCK2 mediates DNA repair systems in TMZ-resistant gliomas.

In this study, we generated MGMT^low^ (or MGMT^−^) TMZ-R glioma cell lines and demonstrated that ROCK2 inhibition reversed TMZ-resistance in glioma cells via impairment of the DNA repair system.

## Results

### ROCK2 expression correlates with the prognosis of TMZ-treated MGMT^low^ recurrent glioma patients

**Fig. 1:**
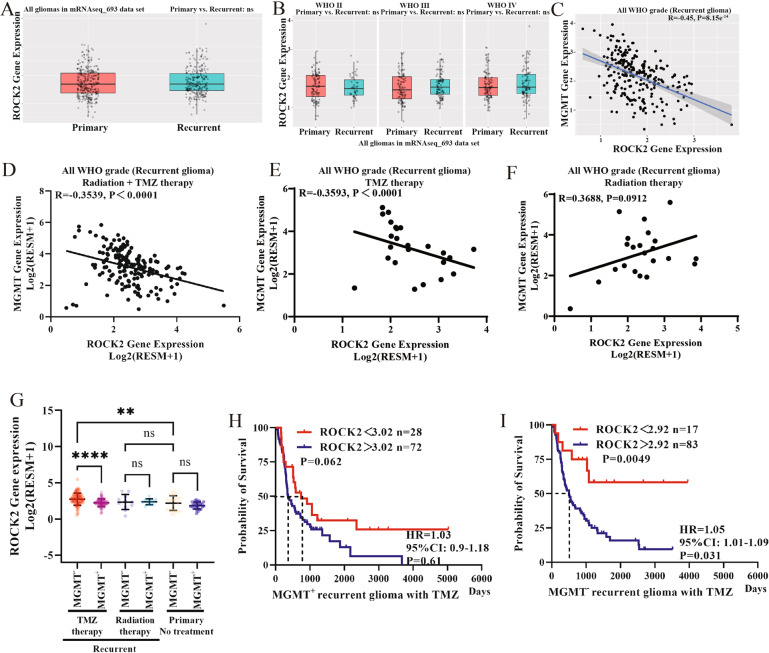
Analysis of correlations between ROCK2, MGMT expressions and TMZ treatment responses in glioma patients in the CGGA database. **A**, **B** ROCK2 gene level in 693 case glioma patients as calculated by CGGA online tools. **C** Co-expression analysis of ROCK2 and MGMT mRNA expression in subset of recurrent gliomas. **D** Co-expression analysis of ROCK2 and MGMT mRNA expression in subset of recurrent gliomas with TMZ + Radiation therapy (*n* = 168). **E** Co-expression analysis of ROCK2 and MGMT mRNA expression in subset of recurrent gliomas with TMZ treatment (*n* = 24). **F** Co-expression analysis of ROCK2 and MGMT mRNA expression in subset of recurrent gliomas with radiation therapy (*n* = 22). **G** ROCK2 mRNA expression levels in recurrent gliomas using mRNA 637 data set. Recurrent group: TMZ-treatment MGMT^−^ (*n* = 12), TMZ-treatment MGMT^+^ (*n* = 12), Radiation treatment MGMT^−^ (*n* = 11), Radiation treatment MGMT^+^ (*n* = 11), TMZ + Radiation treatment MGMT^−^ (*n* = 84), TMZ + Radiation treatment MGMT^+^ (*n* = 84). Primary group: without treatment, MGMT^−^ (*n* = 27), MGMT (*n* = 26). **H**, **I** Survival analysis of recurrent MGMT^high^ and MGMT^low^ glioma patients with different ROCK2 expression. Statistical differences are given as *p < 0.05, **p < 0.01, ***p < 0.005, ****p < 0.001.

In the mRNAseq_693 data set of the Chinese Glioma Genome Atlas (CGGA) [17], ROCK2 gene expression in the recurrent group was not significantly higher than that in the non-recurrent group (Fig. 1A). There were also no observed differences in ROCK2 gene level among various glioma subtypes (Fig. 1B). Interestingly, gene of MGMT was inversely correlated with ROCK2 expression (Fig. 1C) in the recurrent group, especially in the recurrent glioma patient treated with TMZ or TMZ + radiation therapy (Fig. 1D, E). However, no correlation was found between ROCK2 and MGMT gene expression with radiation therapy only (Fig. 1F). MGMT^low^ recurrent glioma patients with TMZ therapy or TMZ + radiation therapy exhibited an increased level of ROCK2 gene (Fig. 1G). Moreover, no alteration of ROCK2 gene was identified in the relapse sample with radiation therapy (Fig. 1G). ROCK2 expression was associated with poor overall survival of MGMT^low^ (MGMT^−^) recurrent gliomas with TMZ therapy (*n* = 100, Fig 1H), compared with MGMT^high^ (MGMT^+^) subset (Fig. 1H, I). Taken together, these findings demonstrated a strong correlation between ROCK2 and TMZ response in MGMT^low^ recurrent glioma patients and might suggest a potential role of ROCK2 on TMZ-R.

### Suppression of ROCK2 lead cell to death and enhances DNA double-strand breaks (DSBs) in TMZ-treated MGMT^low^ TMZ-R glioma cells

**Fig. 2:**
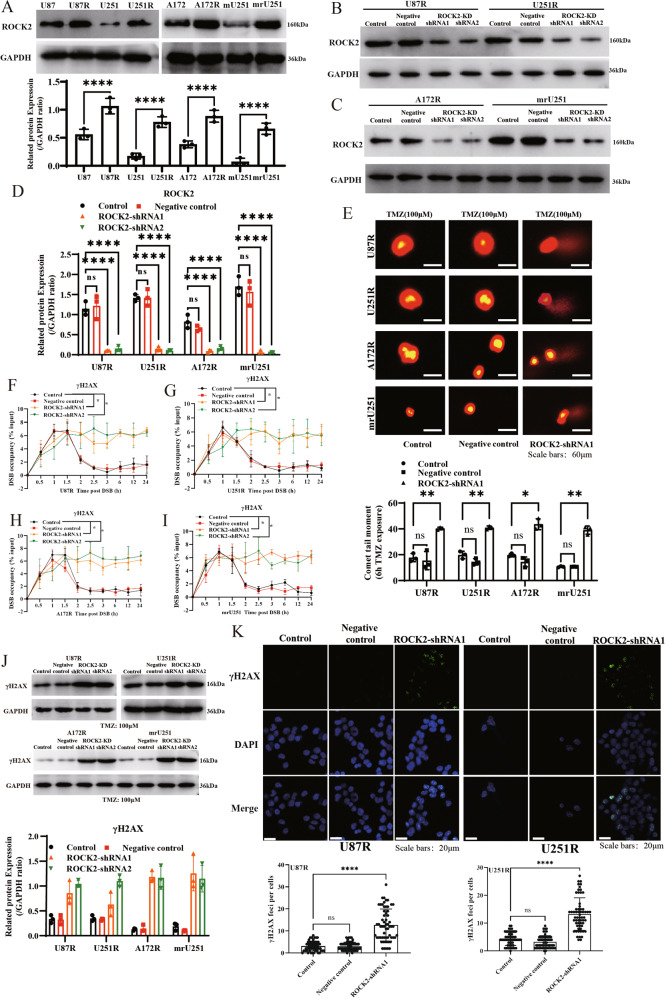
ROCK2 suppression induced DSB in TMZ-R cells. **A** ROCK2 protein expression in TMZ-R cells and parental cells. **B**, **C** ROCK2 protein level was determined with ROCK2-shRNA (ROCK2-KD). **D** WB quantification of **B** and **C**. **E** Neutral comet assay was performed, Scale bars, 60 μm. **F**–**I** DSB-ChIP quantification over time of γH2AX level at the site-specific DSB. **J** γH2AX protein level in ROCK2-KD cells with TMZ (100 μM) for 24 h. **K** IF results of γH2AX-foci in U87R and U251R with ROCK2-KD. Data are mean ± SD of three independent experiments. *p* values determined by ANOVA or two-tailed unpaired *t*-test. Statistical differences compared with the controls are given as *p < 0.05, **p < 0.01, ***p < 0.005, ****p < 0.001.

The MGMT^low^ (U87, U251 and A172) and MGMT^high^ (U138, T98G and U118) cells were used to establish TMZ-R cells (Supplementary Fig. S1A–F). ROCK2 gene expression was increased only in MGMT^low^ TMZ-R cells (Supplementary Fig. S1G) and MGMT expression was substantially upregulated only in MGMT ^high^ TMZ-R cells (Supplementary Fig. S1H). In U87R, U251R and A172R cells, a combination of fasudil (ROCK2 inhibitor) and TMZ treatment was synergistic and inhibited proliferation of MGMT^low^ TMZ-R cells (Supplementary Fig. S1I–R, WB blots were also given in Original Data of WB blots), providing evidence that the TMZ sensitivity observed in fasudil-treated cells was only in MGMT^low^ TMZ-R cells. As a MGMT^low^ cell, U251 cells was used to establish a xenograft tumor, and then followed by a sequential treatment with TMZ at different doses. After four phases, a TMZ-resistant xenograft tumor is established and mrU251 cell line is obtained (details were described in “Methods” and Supplementary Fig. S2). The primary cell mrU251was resistant to TMZ compared with mU251 cells. As showed in Fig. 2A (WB blots were also given in Original Data of WB blots), ROCK2 expression was increased in all MGMT^low^ TMZ-R cells. The combination of ROCK2 knock-down (with shRNA, ROCK2-KD) (Fig. 2B–D; WB blots were also given in Original Data of WB blots) and TMZ treatment enhanced DNA DSBs (Fig. 2E) which were measured by comet assay. Next, as described before [18], a double-strand break and chromatin immunoprecipitation (DSB-ChIP) assay was used to monitor the recruitment of γH2AX to the DSBs site. γH2AX, a marker of DNA damage, was rapidly accumulated on DSBs site with ROCK2-KD after DSB occurred (Fig. 2F–I). And an increased level of γH2AX was determined in ROCK2-KD TMZ-R cells with TMZ treatment (100 μM, 24 h) (Fig. 2J, WB blots were also given in Original Data of WB blots), and the results of immunofluorescence (IF) in TMZ-R with ROCK2-KD showed that the number of γH2AX foci was increased with TMZ-treatment (Fig. 2K).

### ROCK2 directly mediates homologous recombination (HR) repair

**Fig. 3:**
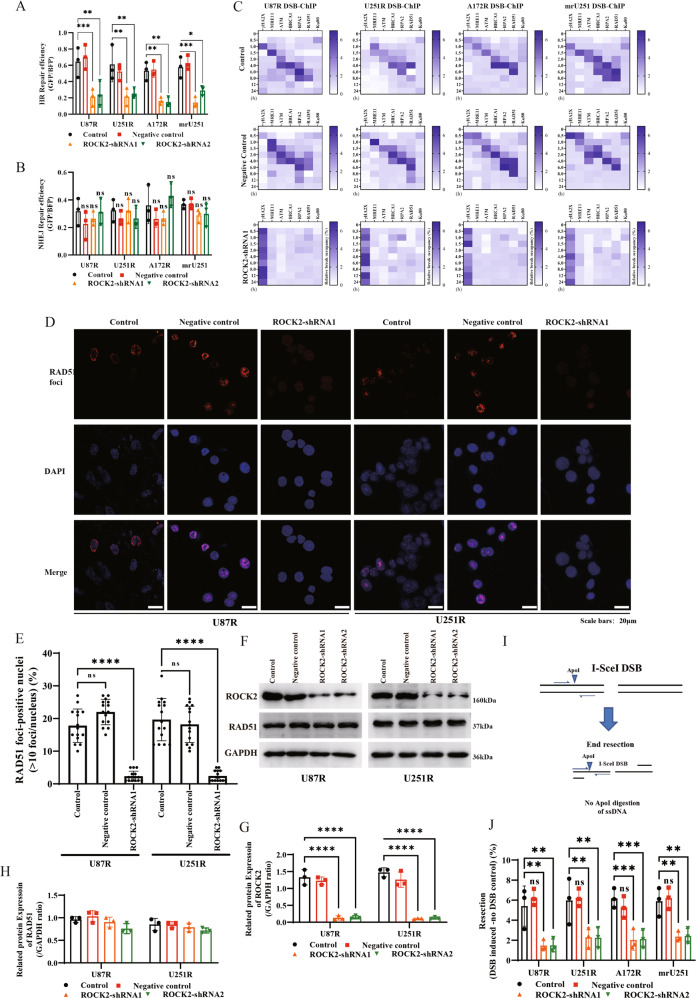
ROCK2-KD suppressed stepwise recruitment of HR factors to DNA DSBs. **A**, **B** ROCK2-KD suppressed HR repair. **C**–**F** Heat maps of relative occupancy of indicated factors at site-directed DSB with ROCK2-KD. **G** Immunofluorescence images of RAD51 in cell nuclei in the indicated cells with TMZ (100 μM), Scale bars, 20 μm. **H** Quantification of RAD51 foci-positive nuclei. **I** Schematic representation of PCR-based DNA end-resection assay. **J** Quantification of end-resection at the same site-specific DSB as used in the DSB-ChIP assay. Data are expressed as mean ± SD. *p* values determined by ANOVA or two-tailed unpaired *t*-test. Statistical differences compared with the controls are given as *p < 0.05, **p < 0.01, ***p < 0.005, ****p < 0.001.

As showed in Supplementary Fig. S3A–C, only HR repair was enhanced in MGMT^low^ cells (U87R, U251R, A172R and mrU251). However, single-strand annealing repair was enhanced in all TMZ-R cell lines (Supplementary Fig. S3D). Next, compared with TMZ-sensitive cells (U87, U251, A172 and mU251), the analysis of DSB-ChIP indicated that γH2AX was rapidly accumulated in MGMT^low^ TMZ-R cells, followed by a coordinated pattern of recruitment of DNA repair factors to sites of DSB damage, including meiotic recombination 11 (MRE11), tat-interactive protein 60 (TIP60, also named KAT5), ataxia telangiectasia-mutated (ATM), replication protein A (RPA), breast cancer susceptibility protein 1 (BRCA1) and RAD51 (Supplementary Fig. S3E–H). Knock-down of ROCK2 reduced the HR repair efficiency (Fig. 3A). Notably, inhibition of ROCK2 did not compromise non-homologous end joining (NHEJ) (Fig. 3B). The DSB-ChIP assay showed that γH2AX rapidly accumulated at the DSB sites in both the control and ROCK2-KD cells and resolved quickly in control cells but persisted in ROCK2-KD cells, suggesting a reduced repair and enhanced DSBs (Fig. 3C). As showed in Fig. 3 and Supplementary Fig. S4, in MGMT^low^ TMZ-R glioma cells, the γH2AX signal persisted in ROCK2-KD cells or fasudil (ROCK2 inhibitor, 10 μM) treatment, whereas the subsequent recruitment of the HR factors was substantially attenuated, suggesting an impaired repair in MGMT^low^ TMZ-R cells. A nucleus-location (NLS) RAD51-RFP-NLS plasmids was transfected into U251R and U87R cells, and after occurrence of DSB, the number of RAD51 foci (RFP) in nucleus and positive cells were reduced (Fig. 3D, E) with ROCK2-KD (Fig. 3F, G, WB blots were also given in Original Data of WB blots), and no alteration of RAD51 expression was detected by western blot (Fig. 3F, H). The results indicated that ROCK2-KD impaired the recruitment of repair factors to the DSB site. HR repair is dependent on DNA end-resection at the DSB. To examine end-resection, a site-specific method was used to measure single-strand DNA (ssDNA) production after activation of I-SceI (Fig. 3I). ssDNA production was reduced in ROCK2-KD TMZ-R cells, suggesting a defect in end-resection (Fig. 3J). These data suggested the direct regulation of HR repair by ROCK2.

### Suppression of HR repair via ROCK2 leads to a useless NHEJ repair

**Fig. 4:**
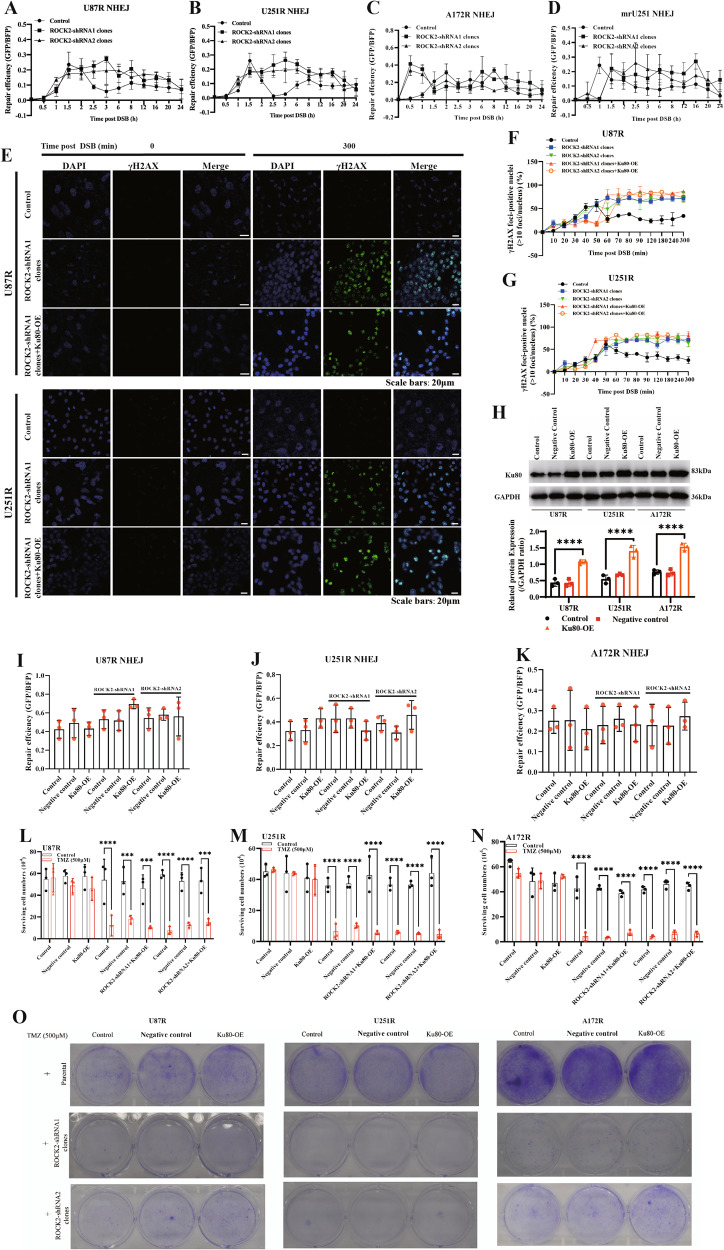
Enhancement of NHEJ by ROCK2 inhibition was useless for DNA repair in TMZ-R cells. **A**–**D** NHEJ versus time was determined in ROCK2-KD cells. **E**–**G** γH2AX foci-positive cells were detected by IF and counted, Scale bars, 20 μm. **H** Ku80 expression was determined with Ku80-overexpression plasmids. **I**–**K** NHEJ for 24 h was determined in ROCK2-KD cells with Ku80-OE. **L**–**N** Cells were counted with TMZ and Ku80-OE in ROCK2-KD cells. **O** Cells were stained with TMZ and Ku80-OE. Data are shown as mean ± SD of three independent experiments. *p* values determined by ANOVA *t*-test. Statistical differences compared with the controls are given as *p < 0.05, **p < 0.01, ***p < 0.005, ****p < 0.001.

The choice of DNA break repair pathway between NHEJ and HR depends on competition between HR and NHEJ factors at DNA break sites [19]. While HR is restricted, NHEJ is active. ATM activity is shown to be necessary for the release of both Ku and DNA-PKcs components of the NHEJ apparatus [20]. In U87R, U251R, A172R and mrU251 cells, it was found that NHEJ repair was enhanced at 1.5 h post DSB occurrence and then reduced, and ablation of ROCK2 induced a persistent NHEJ repair (Fig. 4A–D). γH2AX foci was observed in ROCK2-KD cells by IF (Fig. 4E–G). Ku80 was overexpressed (Ku80-OE) in ROCK2-KD TMZ-R cell with MGMT low-expression (Fig. 4H, WB blots were also given in Original Data of WB blots). After DSB induction, the γH2AX foci was persisted and highly detected in ROCK2-KD cells. In Ku80-OE TMZ-R cells with ROCK2-KD, γH2AX foci number was still highly detected and no different was found with ROCK2-KD only. Next, the repair efficiency of NHEJ was not enhanced in 24 h (Fig. 4I–K). Moreover, cell proliferation remained suppressed under TMZ stimulation (Fig. 4L, O, while DMSO had no significative effects on all cell lines tested). Collectively, our data indicated that NHEJ was enhanced with impairment of HR by ROCK2 inhibition, which may not affect DNA repair under TMZ stimulation in MGMT^low^ TMZ-R cells.

### ROCK2 modulates HR via ATM

**Fig. 5:**
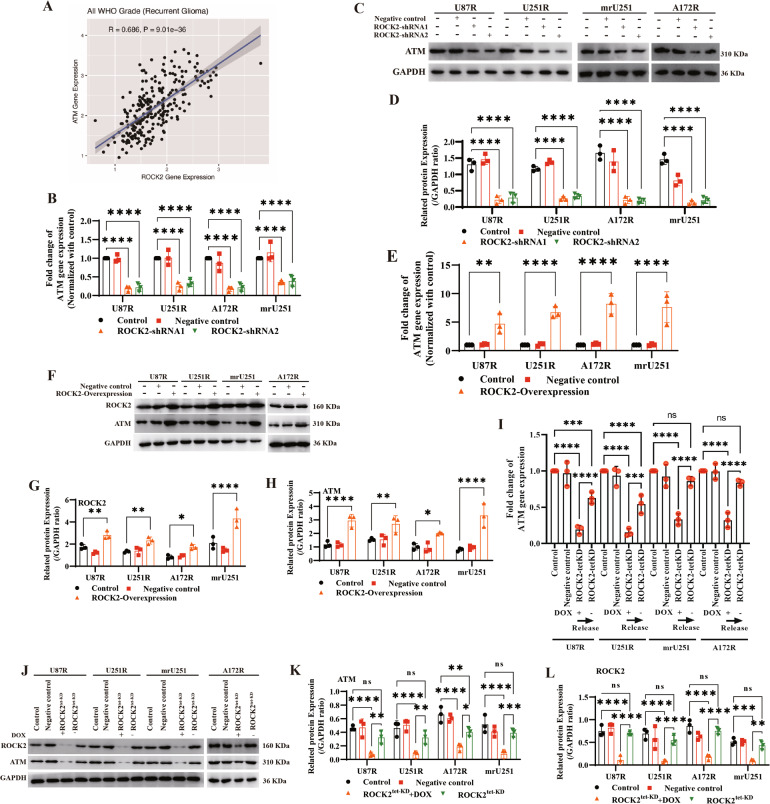
ROCK2 directly regulated ATM expression. **A** Co-expression analyze of ATM with ROCK2 using CGGA data. **B** Gene expressions of ATM was determined with ROCK2-KD. **C**, **D** ATM protein levels were determined with ROCK2-KD. **E**–**H** Gene and protein expressions of ATM and ROCK2 were determined with ROCK2-OE. **I** Gene expression of ATM was determined with ROCK2-tet-KD. **J**–**L** Protein expression of ATM was determined with ROCK2-tet-KD. Data are expressed as mean ± SD of three independent experiments. *p* values determined by ANOVA or two-tailed unpaired *t*-test. Statistical differences compared with the controls are given as *p < 0.05, **p < 0.01, ***p < 0.005, ****p < 0.001.

Analysis of mRNAseq_693 data set (CGGA) showed that both MRE11 (Supplementary Fig. S5A) and ATM (Fig. 5A) were identified as the most correlated potential regulatory partners of ROCK2 with Pearson scores >0.6. It was also found that ROCK2 suppression reduced ATM gene and protein expression (Fig. 5B–D; WB blots were also given in Original Data of WB blots) with no alteration of MRE11 expression (Supplementary Fig. S5B–D; WB blots were also given in Original Data of WB blots). A relatively strong negative correlation of KAT5 and ROCK2 was determined (Supplementary Fig. S6A), and BRCA1, RAD51 and RPA2 had no correlation with ROCK2 expression (Supplementary Fig. S6B–D). In MGMT^low^ TMZ-R cells, KAT5 gene expression was upregulated with ROCK-KD in U87R cell, and no other detectable gene level changes were determined. ROCK2 overexpression (ROCK2-OE) upregulated the ATM level (Fig. 5E–H; WB blots were also given in Original Data of WB blots). In addition, ATM gene and protein levels were repressed with DOX treatment (DOX-induced ROCK2-KD) and recovered with DOX release (Fig. 5I–L; WB blots were also given in Original Data of WB blots). In an animal model of U251R xenograft model as we reported before [10], the immunohistochemistry (IHC) and western blot results of tumor tissue indicated that ATM expression was decreased with fasudil treatment, and γH2AX level was increased with fasudil and TMZ treatment (Supplementary Fig. S7, WB blots were also given in Original Data of WB blots). These results suggest that ROCK2 directly regulates ATM expression.

**Fig. 6:**
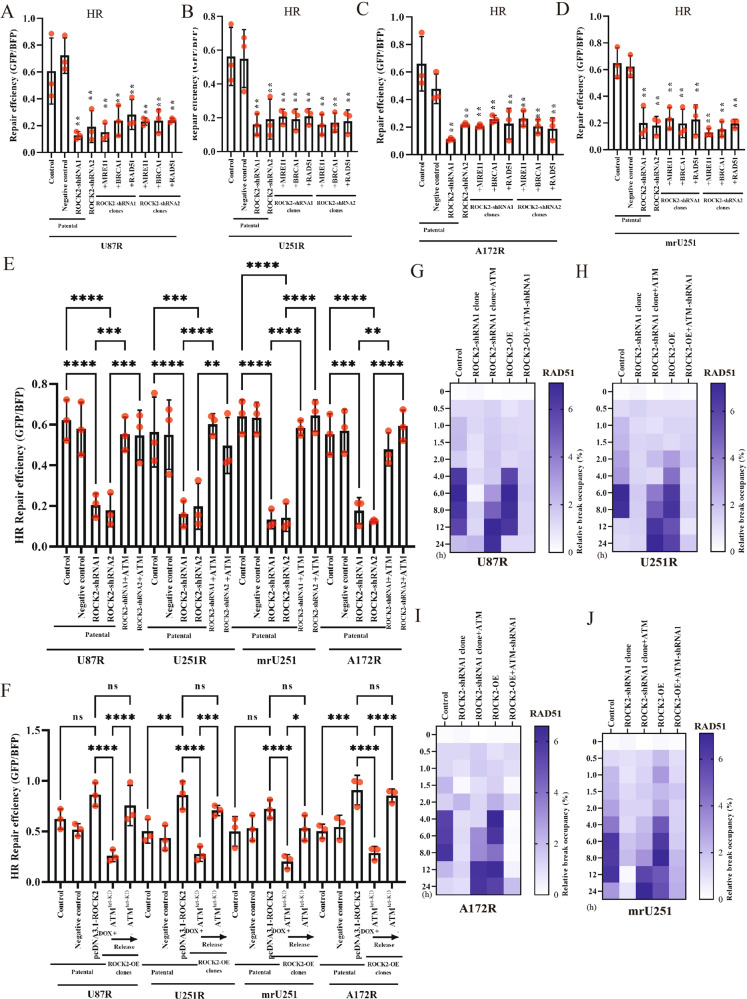
ROCK2 directly regulated HR via ATM. HR efficiency was determined with supplementary of MRE11, RAD51, BRCA1 in U87R (**A**), U251R (**B**), mrU251 (**C**) and A172R (**D**) of ROCK2-KD clones cells. **E** HR efficiency was determined with supplementary of ATM in ROCK2 KD cells. **F** HR efficiency was determined with supplementary of ATM-tet-KD in ROCK2-OE cells. RAD51 levels at the site-specific DSB, **G** U87R, **H** U251R, **I** mrU251, **J** A172R. Data are expressed as mean ± SD of three independent experiments. *p* values determined by ANOVA or two-tailed unpaired *t*-test. Statistical differences compared with the controls are given as *p < 0.05, **p < 0.01, ***p < 0.005, ****p < 0.001.

In rescue experiments performed in ROCK2-KD TMZ-R cells, there was no detectable rescue of HR repair by complements of MRE11, BRCA1 and RAD51 (Fig. 6A–D). Notably, complements of ATM induced an HR repair recovery (Fig. 6E). HR repair was repressed with ATM-KD (DOX-induced ATM-KD) and recovered with DOX release (Fig. 6F). As shown in Fig. 6G–J, RAD51 was recruited in ROCK2-KD cells with ATM complements, indicating that HR repair was impaired. Taken together, the above results established that ROCK2 modulated HR repair through ATM.

### ROCK2 regulates ATM expression via ZEB1

**Fig. 7:**
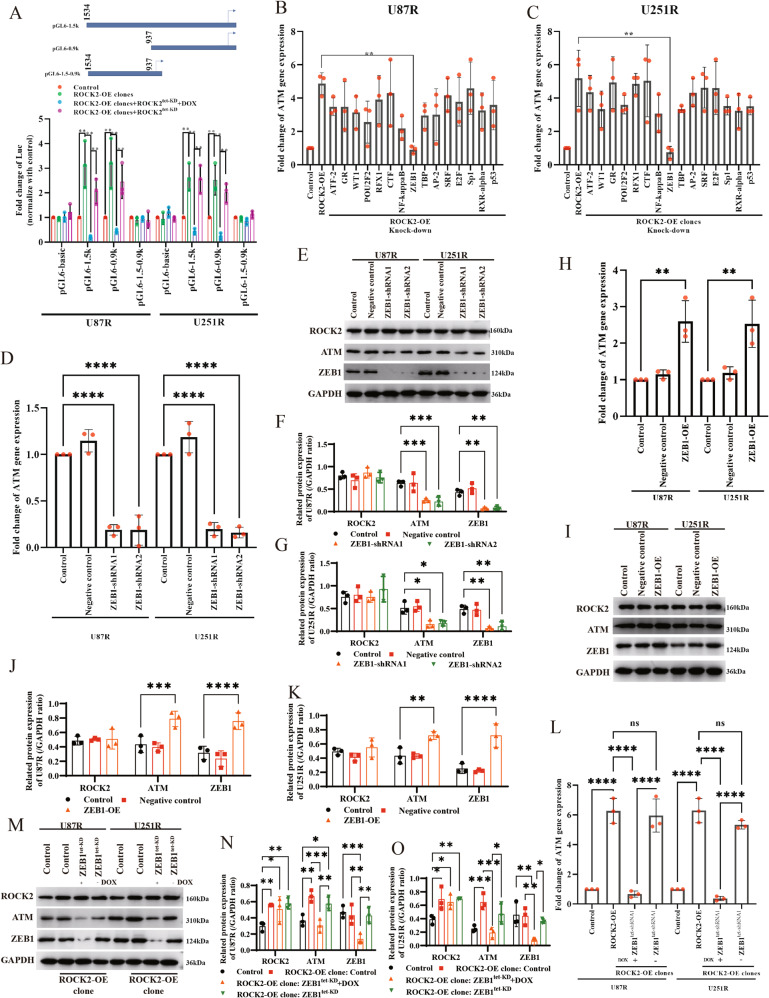
ROCK2 mediated ATM expression via ZEB1. **A** Different wild-type ATM promoter luciferase reporter constructs were used for binding assay. **B**, **C** ATM gene expression of U87R and U251R was determined with different siRNA of TFs as indicated. **D** ATM gene expression was determined with ZEB1 knock-down. **E**–**G** ATM protein expression was determined with ZEB1 knock-down. WB results: **E**, Quantification of WB: **F** and **G**. **H** ATM gene expression was determined with ZEB1 overexpression. **I**–**K** ATM protein expression was determined with ZEB1 overexpression. WB results: **I**, Quantification of WB: **J** and **K**. **L** ATM gene expression was determined with ZEB1-tet-KD in ROCK2-OE cells. **M**–**O** ATM gene expression was determined with ZEB1-tet-KD in ROCK2-OE cells. WB results: **M**, Quantification of WB: **N** and **O**. Data are shown as of three independent experiments. *p* values determined by ANOVA or two-tailed unpaired *t*-test. Statistical differences compared with the controls are given as *p < 0.05, **p < 0.01, ***p < 0.005, ****p < 0.001.

To explore the potential mechanisms of ROCK2 regulates ATM expression, we asked which transcription factors (TFs) would be involved in. The luciferase assay showed that 0.9k carried a transcriptional activation function for ATM gene (Fig. 7A). Databases of known TFs were systematically searched using the LASAGNA-Search 2.0 web tool, which may bind to ATM promotor. It was found that ATF-2, GR, WT1, POU2F2, RFX1, CTF, NF-kappaB, ZEB1, TBP, AP-2, SRF, E2F, Sp1, RXRα and p53 bond to this motif. TFs were knocked down by siRNA in ROCK2-OE cells, and it was determined that only ZEB1-KD suppressed the mRNA level of ATM (Fig. 7B, C), indicating that ZEB1 could be involved in ATM expression regulated by ROCK2. To validate this conjecture, ZEB1 was knocked down by shRNA, and the results showed that ATM expression of gene and protein was reduced (Fig. 7D–G; WB blots were also given in Original Data of WB blots), and overexpression of ZEB1 in TMZ-R cells increased ATM levels (Fig. 7H–K; WB blots were also given in Original Data of WB blots). DOX-induced ZEB1 depletion decreased ATM expression, and removal of DOX recovered ATM level in ROCK2-OE cells (Fig. 7L–O; WB blots were also given in Original Data of WB blots). As showed in Supplementary Fig. S8A, B, the enrichment of ZEB1 bound to ATM promotor was enhanced. In addition to its well-characterized role as a repressor, ZEB1 has also been implicated in transcriptional activation with few observations. In general, ZEB1 inhibited genes expression via binding to the E-box motif [21]. In addition to the E-box, the HMG-box matches the consensus binding site for ZEB1 [22]. Therefore, we use constructed five promotor mutation reporter plasmids (Supplementary Fig. S8C, D). The result of electrophoretic mobility shift assay (EMSA) showed that ZEB1 could bind to E-box in vitro (Supplementary Fig. S8F; WB blots were also given in Original Data of WB blots). Mutation of site 646-635 (646-635^mu^) which contains E-box, suppressed ATM expression (Supplementary Fig. S8E, G, H; WB blots were also given in Original Data of WB blots) and in TMZ-R ROCK2 KD cells, 646-635^mu^ with additional of ZEB1 protein did not completely restore ATM expression (Supplementary Fig. S8I, J; WB blots were also given in Original Data of WB blots). In ROCK2-OE cells, KD of ZEB1 reduced ATM expression and additional of ZEB1 in 646-635^mu^ cells did not recover ATM expression (Supplementary Fig. S8K, L, WB blots were also given in Original Data of WB blots). These results strongly suggested that ZEB1 binds to E-box and induces the expression of ATM. In summary, we found that ROCK2 regulated ATM expression by ZEB1 and ZEB1 directly binds to the ATM promoter.

### ROCK2 transforms ZEB1 as a gene activator via yes-associated protein 1 (YAP1)

**Fig. 8:**
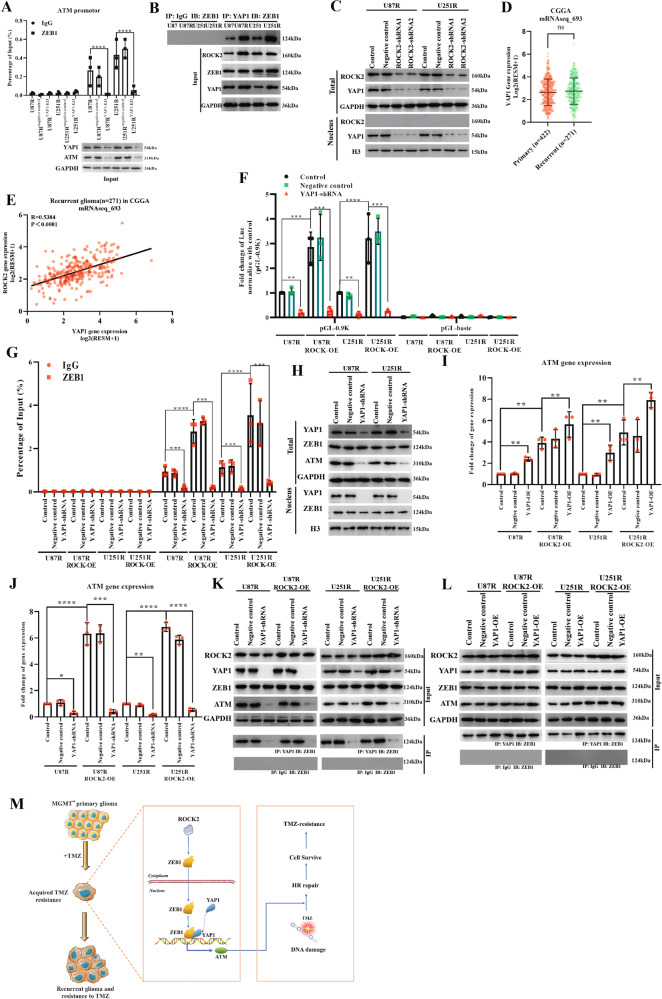
ROCK2 transformed ZEB1 to activator via YAP1. **A** ChIP assay for ATM promoter with YAP1-siRNA, and expression of ATM protein was determined with YAP1-siRNA. **B** IP assay for YAP1 and ZEB1. **C** ROCK2 regulated YAP1 expression. **D** YAP1 expression in 693 glioma patients as determined by CGGA online tools. **E** Co-expression analysis of YAP1 and ROCK2 mRNA level in subset of recurrent gliomas. **F** Luciferase activities were determined after transfection with YAP-1 shRNA in U87R, U87R-ROCK2OE, U251R and U251R-ROCK2-OE cells. Luciferase were detected. **G** ChIP assay of ZEB1 on ATM promotor was determined with transfection of YAP1-shRNA in U87R, U87R-ROCK2OE, U251R and U251R-ROCK2OE cells. **H** YAP1, ZEB1 and ATM levels in YAP1-KD cells were determined by WB. **I**, **J** ATM gene expression was detected by Q-PCR after transfection of YAP-shRNA or YAP-overexpression plasmid in U87R, U87R-ROCK2OE, U251R and U251R-ROCK2OE cells. **K**, **L** ROCK2, YAP1, ZEB1 and ATM expressions were determined by WB after transfection of YAP-shRNA or YAP-overexpression plasmid in U87R, U87R-ROCK2OE, U251R and U251R-ROCK2OE cells. **M** Mechanisms through which ROCK2 mediates TMZ-resistance in MGMT^low^ glioma. Data are expressed as mean ± SD of three independent experiments. *p* values determined by ANOVA or two-tailed unpaired *t*-test. Statistical differences compared with the controls are given as *p < 0.05, **p < 0.01, ***p < 0.005, ****p < 0.001.

ZEB1 activation requires interaction with co-activators. Co-activators of ZEB1, such as Smad3, P300/CBP associated factor (PCAF), lymphoid enhancer-binding factor 1 and yes-associated protein 1 (YAP1), were knocked down. The ChIP and western blot assay showed that only YAP1-KD reduced ATM promotor activity and ATM protein expression (Fig. 8A and Supplementary Fig. S9A–D; WB blots were also given in Original Data of WB blots), suggesting that YAP1 could be the co-activator for ZEB1. Histone H3 K27 trimethylation (H3K27me3) was associated with silenced genes, whereas histone H3 K4 trimethylation (H3K4me3) and H3 K79 dimethylated (H3K79me2) were most often associated with transcriptionally active genes [23]. With unchanged protein level, results of the ChIP assay showed that enrichment level of H3K4me3 and H3K79me2 were increased in MGMT^low^ TMZ-R cells for ATM promotor activity (Supplementary Fig. S9E–I), indicating a transcriptionally active manner of genes expression.

YAP1 is a co-activator for ZEB1 [24, 25] and can be regulated by ROCK2 [26, 27]. Therefore, the binding of YAP1 to ZEB1 was assessed d in TMZ-R cells. The results revealed that the binding of YAP1 to ZEB1 was enhanced by increased expression of YAP1 (Fig. 8B and Supplementary Fig. S10A; WB blots were also given in Original Data of WB blots). Consistent with previous reports, ROCK2-KD suppressed YAP1 expression (Fig. 8C and Supplementary Fig. S10B, C; WB blots were also given in Original Data of WB blots). Analysis of the CGGA-mRNAseq_693 data set revealed that no differences in YAP1 expression among various glioma subtypes, but YAP1 was identified as the most correlated potential regulatory partner of ROCK2 with Pearson scores >0.5 (Fig. 8D, E). In U87R and U251R cells, ROCK2-OE increased the activation of the promoter, which contained ZEB1-binding site (pGL-0.9k), and knock-down of YAP1 reversed this effect (Fig. 8F). Meanwhile, the ChIP assay demonstrated that YAP1-shRNA remarkably decreased the binding of ZEB1 to the ATM promotor (Fig. 8G). In U87R and U251R cells, YAP1-KD significantly decreased ATM expression level, with unchanged ZEB1 expression in the nucleus (Fig. 8H and Supplementary Fig. S10D, E; WB blots were also given in Original Data of WB blots). YAP1-KD suppressed ATM expression by reduced YAP1-ZEB1 binding (Fig. 8J, K). Contrarily, YAP1 overexpression in TMZ-R and ROCK2-OE cells resulted in increased ATM expression as well as the binding of YAP1 to ZEB1 (Fig. 8I, L and Supplementary Fig. S10F–I; WB blots were also given in Original Data of WB blots). Overall, these findings suggested that YAP1 regulated ZEB1 by specific-site binding without changing its expression.

## Discussion

A recent study revealed that MGMT-negative deficient gliomas were responsive to TMZ and would still develop therapeutic resistance, leading to treatment failure. Previous work from our laboratory determined that ROCK2 was the important mediator of acquired resistance to TMZ in glioma cells and the mechanism underlying the regulation of ABCG2, which was functions as a high-capacity drug efflux transporter [10]. In a separate project in our laboratory, we determined a role of ROCK2 in EMT-induced gemcitabine resistance in pancreatic cancer cells, and we revealed that ROCK2 induced increasement of ZEB1 expression and enhancement of DNA repair system [28]. In this study, we found that ROCK2 was a critical regulator in MGMT^low^ TMZ-R glioma models. ROCK2 regulated DNA repair via HR repair, and the mechanism involved a direct regulation of ATM via YAP1/ZEB1. Unlike TMZ, for a cell to resist treatment with dFdC (one active metabolite of gemcitabine), the chain-terminating nucleoside analog must be removed to allow replication restart [29]. However, the main HR repair factors such as ATM [30], RAD50 [30] or MRE11 [31], have been reported to remove dFdC but not increase HR repair efficiency. In our study of TMZ-resistance, HR efficiency was enhanced depended on ROCK2 induced ATM overexpression. Our results indicated a diverse role for DNA repair factors on TMZ-resistance compared with gemcitabine.

Due to hypermethylation in recurrent gliomas treated with TMZ, deficiency of MMR pathway and MGMT expression led to enhancement of DNA repair systems such as HR, NHEJ and base excision repair, suggesting that a hyperactive DNA repair system was essential for TMZ-resistance [5]. In our MGMT^low^ TMZ-resistant models, HR and NHEJ repair were increased in TMZ-R cells, which agrees with previous findings.

The role of ROCKs in chemo-resistance has been widely reported. Intriguingly, ROCKs-myosin II ablation specifically kills resistant cells via unresolved DNA damage [15]. Our data are consistent with previous findings, where ROCK2 inhibition was a strong inducer of DSB in resistant cells. However, how ROCKs regulated DNA repair is still unclear. Melanoma cells with low levels of Rho-ROCK-driven actomyosin were subjected to oxidative stress-dependent DNA damage [32]. Inhibition of ROCK2 resulted in reduced expression of H2AX and MRN complex proteins, critical to the repair of DSBs [16]. Unlike the results mentioned, we found that inhibition of ROCK2 decreased the ATM level in U87R and U251R cells, induced DSB and impaired DNA repair, suggesting that a novel mechanism was responsible for DSB repair in TMZ-R gliomas.

Furthermore, our findings showed that inhibition of HR induced a transient NHEJ repair which was ineffective for DNA repair in U87R and U251R cells. NHEJ is employed to rejoin double-ended DSBs that occur when the two strands of the DNA double helix are simultaneously broken in close proximity [33]. Several layers of control dictate DSB-repair-pathway choice between NHEJ and HR, including activation of HR by cyclin-dependent kinase activity, or direct competition between HR and NHEJ promoting their factors at DSB sites [34, 35]. Although ATM modulates the ability of CtIP to promote Ku removal, it is unclear what the impact of losing this function would be in ATM-deficient cells [20, 36]. Additionally, much research interest has been focused on the competition between the HR-promoting factor BRCA1 and the NHEJ-promoting factor 53BP1 [37, 38], however, the mechanisms underlying this antagonism remain elusive.

The main transcriptional function of ZEB1 is to suppress the expression of its target genes, such as epithelial markers (E-cadherin) [39]. Several cofactors were also recruited during the transcriptional suppression process of ZEB1 for its downstream target genes. It has been proposed that ZEB1 can act as an activator or repressor to gene transcription depending on the TF it interacts with. ZEB1-CtBP directly binds to DNA and mediates its repressor effects [40]. ZEB1/p300/PCAF or ZEB1/YAP1 complex promoter gene expression [25, 41, 42]. In the current study, ROCK2 can increased the nuclear expression and transcriptional activity of YAP1 [26], and we found that YAP1 can specifically and directly interacted with ZEB1, converting ZEB1 from a transcriptional repressor to a transcriptional activator. Further studies are needed to dissect the underlying mechanisms.

In summary, our study revealed that ROCK2 increased HR repair via up-regulating ATM expression in MGMT^low^ TMZ-resistant glioma cells. In TMZ-R cells, ROCK2 transformed ZEB1 into an activator for ATM expression via YAP1 (Fig. 7M). These findings highlight the potential for exploiting a resistance mechanism regulated by ROCK2, providing evidence for the use of ROCK inhibitors in the clinical therapy of MGMT^low^ TMZ-resistant glioma.

## Materials and methods

### Cell culture

Human glioma cell lines U87, U251 and A172 were obtained from the Type Culture Collection of the Chinese Academy of Sciences (Shanghai, China). T98G cell line were gift from QW. U118, U138 and 293T were obtained from Shanghai Zhongqiao Xinzhou Biotechnology Co.,Ltd. All cell lines were authenticated with methods of short tandem repeat. The TMZ-resistant cells were established from varies of cells. TMZ-R cells were cultured with 50 μM TMZ. All glioma and resistance cell lines were cultured in DMEM or MEM containing 10% FBS, and maintained in a humidified atmosphere of 95% air, 5% CO_2_ at 37 °C. Cells that had been passaged 4–10 times were used for the experiments. The cells were passaged twice a week and discarded after 20 passages.

### Gene expression profiling and survival analysis

The CGGA online tool (http://www.cgga.org.cn/analyse/RNA-data.jsp) and GraphPad Prism 8.0 was used for the analysis of co-expression in gliomas database. All of the survival curves were generated using the GraphPad Prism 8.0. The gene expression data sets and related clinical data can be downloaded from the following websites: CGGA (http://www.cgga.org.cn) and calculated as log2(RESM + 1) for analysis.

### Reagents and plasmids

TMZ (S1237, dissolved in dimethyl sulfoxide, 100 mM) and fasudil (S1573, dissolved in PBS, 100 mM) were purchased from Selleck Chemicals Inc (Selleck Chemicals Inc. Houston, USA). Doxycycline was purchased from MedChemExpress (NJ, USA). Sources of the antibodies were as follows: Anti-ROCK2 (21645-1-AP, 1:1000 for western blotting (WB)), anti-ATM (27156-1-AP, 1:1000 for WB, 1:150 for DSB-ChIP, 1:200 for IHC), anti-RAD51 (14961-1-AP, 1:1000 for WB, 1:150 for DSB-ChIP, 1:200 for IF, 1:200 for IHC), anti-BRCA1 (22362-1-AP, 1:1000 for WB, 1:200 for DSB-ChIP, 1:200 for IHC), anti-TIP60 (also named anti-KAT5, 10827-1-AP, 1:2000 for WB, 1:100 for DSB-ChIP), anti-GAPDH (10494-1-AP, 1:3000 for WB), anti-MRE11 (10744-1-AP, 1:2000 for WB, 1:100 for DSB-ChIP, 1:200 for IHC), anti-RPA2 (10412-1-AP, 1:1000 for WB, 1:100 for DSB-ChIP), anti-Ku80 (also named XRCC5, 16389-1-AP, 1:2000 for WB, 1:100 for DSB-ChIP), anti-Histone-H3 (17168-1-AP, 1:3000 for WB), anti-ZEB1 (21544-1-AP, 1:1000 for WB, 1:200 for ChIP), anti-YAP1 (13584-1-AP, 1:2000 for WB, 1:200 for IP), HRP-conjugated Affinipure Goat Anti-Rabbit IgG (H + L) (SA00001-2, 1:10,000 for WB) were purchased from Proteintech Group, Inc (IL, USA). Anti-γH2AX (Anti-Phospho-Histone H2AX (Ser139), bsm-52163R, 1:1000 dilution for WB, 1:50 for DSB-ChIP, 1:100 for IF) was purchased from Beijing biosynthesis biotechnology CO., Ltd (Beijing, China). Anti-ROCK2 (phospho Y722, ab182648, 1:1000 dilution for WB) was purchased from Abcam (MA, USA). Anti-Tri-Methyl-Histone H3 (Lys4) (9751, 1:1000 dilution for WB, 1:200 dilution for ChIP), anti-Tri-Methyl-Histone H3 (Lys27) (9733, 1:1000 dilution for WB, 1:150 dilution for ChIP) was purchased from Cell Signaling Technology, Inc. (MA, USA). Anti- Histone H3K79me2 (39144, 1:2000 dilution for WB, 1:100 dilution for ChIP) was purchased from Activemotif (CA, USA). Fluorophore-conjugated secondary antibodies (keyFluor 488 and 568) were from Keygen Biotech. Stbl3 and DH5α Chemically Competent Cell was from Shanghai Weidi Biotechnology Co., Ltd (Shanghai, China). pCDNA3.1-Flag-His-ATM wt, pCDNA3.1-ZEB1, pCDNA3.1, pGL6, Tet-pLKO-Puro, pLP1, pLP-VSVG and pLP2 were obtain from MiaoLing Plasmid Sharing Platform. pLVX-shRNA2-zsgreen was from Sangon Biotech Co., Ltd. (Shanghai, China).

### Western blotting analysis

For total cell lysis, cells were lysed in extraction buffer (RIPA, P0013B, Beyotime, Shanghai, China) for 1 h on ice. The lysates were centrifuged at 12,000 × *g* for 20 min. For extracting the nuclear and cytoplasmic protein extraction, a nuclear and cytoplasmic protein extraction Kit (KGP150, KenGen, Nanjing, China) was used following the instructions of the manufacturer. The protein concentration was quantified by BCA assay (P0010S, Beyotime, Shanghai, China). Western blots were performed as previously described [43, 44].

### Neutral comet assay

Neutral comet assays on cell line samples were performed as previously described [45, 46]. After TMZ treatment, cells were washed, and if required, recovered for 6 h in media without TMZ. Following trypsinization, cells were resuspended in PBS at a concentration of 2 × 10^5^ cells per ml. Then cells were processed with the comet assay reagent kit (KGA240-100, KeyGen BioTech, Nanjing, China).

### DSB-ChIP assays

DSB-ChIP assays were performed as previously described [18]. Briefly, protein A agarose/salmon sperm DNA beads were added to HR report plasmid transfected cells after I-SceI treatment and incubated at 4 °C for 120 min under constant agitation. Next, the beads were pelleted at a maximum speed of 5 min. The beads were washed successively three times each with wash buffer. The precipitated DNA was analyzed with quantitative polymerase chain reaction (Q-PCR) using the following primers: 5′-TTATTGTGCTGTCTCATCATT-3′ (forward) and 5′-GTGCTGCATGCTTCTTCGGCA-3′ (reverse). Q-PCR was performed using AceQ Universal SYBR qPCR Master Mix (Q511-02, Vazyme, Nanjing, China) and Applied Biosystems Step One Plus Real-Time PCR machine.

### In vitro DNA double-strand break repair

DNA DSB repair reporter plasmids were used to assess the effect of TMZ-R cells on DDR following a previously described protocol [47]. The system is based on an enhanced green fluorescent protein (EGFP) reporter for each type of repair. Plasmids are linearized by restriction digest with I-SceI endonuclease (R0694S, NEB, MA, USA), exposing “broken” DNA ends, and transfected into cells with ExFect transfection reagent (Vazyme Biotech, Nanjing, China) following the manufacturer’s instructions. If the repair machinery within cells is active, the linear break is repaired, reconstituting the EGFP gene and emitting a green fluorescence readout. In both instances, p-EBFP2-N1 plasmid (MiaoLing Plasmid Sharing Platform) was included as an internal transfection control.

### DNA end resection assay

DNA end resection at the site of I-SceI-induced DSB was monitored using the same approach as described in previous studies [18]. I-SceI DNA DSB was induced, and cells were collected 3 h after the addition of the ligands. Genomic DNA was extracted. Genomic DNA was digested with ApoI (R0566V, NEB, MA, USA) or HincII (R0103V, NEB, MA, USA) for 5 h at 37 °C followed by heat inactivation at 80 °C for 15 min. Q-PCR was performed using AceQ Universal SYBR qPCR Master Mix (Q511-02, Vazyme, Nanjing, China) and Applied Biosystems Step One Plus Real-Time PCR machine. The primers of the I-SceI restriction cut site were F: GCTAACCATGTTCATGCCTTCT and R: TAGTGGCGGCGGATCTGAAT. The β-Actin control primers were F: GGGCCTCAGGTGATAAATTCTG and R: CCTGAGTCCAAAGGCTGTTTG.

### Q-PCR analysis

Total RNA was extracted using TRIzol reagent (Thermo Fisher). Total RNA was reverse-transcribed to cDNA using the First-Strand cDNA synthesis superMix kit (TransGen Biotech). Real-time PCR was performed using the AceQ qPCR SYBR Green Master Mix Kit (Q511-02, Vazyme, Nanjing, China). The primer sequences used in this study are shown in Supplementary Table 1.

### Immunoprecipitation assay

Aliquots of cell lysates were incubated with antibodies at 4 °C for 2 h and then precleared with protein-A/D-Sepharose (Beyotime, Hangzhou, China) at 4 °C overnight. Immunoprecipitated complexes were subjected to WB with the primary antibody, followed by peroxidase-conjugated appropriate secondary antibody and visualized by 5200 chemiluminescence imaging system (Tenon, Shanghai, China).

### Plasmid construction, knock-down and overexpression assay

Cells were transfected for 24 h with siRNA (Synthesized by Sangon Biotech), control-siRNA (Obtained from Sangon Biotech), plasmid and control plasmid using ExFect transfection reagent (Vazyme Biotech, Nanjing, China) for 24 h. The RNA sequences are shown in Supplementary Table 2.

The shRNA plasmids were established based on instruction of pLVX-shRNA2 and Tet-pLKO-Puro. Empty plasmids were digested with a restriction endonuclease including BamHI (1605, Takara Biomedical Technology, Beijing, China), EcoRI (1611, Takara Biomedical Technology, Beijing, China) and AgeI (R0552V, NEB, MA, USA) and ligated with the annealed product. The mixture was incubated with T4 ligase (DNA Ligation Kit Ver.2.1, 6022, Takara Biomedical Technology, Beijing, China) for 4 h. The DNA sequences are shown in Supplementary Table 3.

For overexpression plasmids, the full-length gene was amplified by PCR from human genomic DNA with different sticky ends and integrated into pcDNA3.1. The DNA sequences are shown in Supplementary Table 4.

Based on the published literature [48], human ATM promoter (−1534/+235) sequences were obtained by PCR from human genomic DNA and cloned into a pGL6 vector. Mutagenesis of the E2‑box in the human ATM promoter was performed using a QuickMutation™ Site-directed Gene Mutagenesis Kit (D0206, Beyotime, Shanghai, China). The primers were: wild-type (wt) 1.5k forward 5′-ACCGAAACTCAACTAGATTACC‑3′, wt 0.9k forward 5′-CATTTCACACCTCTACACTGG‑3′, wt 0.4k forward 5′-CCTCAAAGGTCCTTCTGTCC‑3′ and wt reverse 5′-CAAACCCTGCGTGACTGC‑3′. For directed mutation of ATM promoter, the primers were: 932-921-mu: forward 5′-TGGCATTTCACAtaTCTACACTGGACGACGTATTG‑3′, reverse 5′-CAATACGTCGTCCAGTGTAGAatTGTGAAATGCCA‑3′, 815-909-mu: forward 5′- GCCAGGAAGGTCTCTCTAgtAAGATGGGGGGCCGGG‑3′, reverse 5′- CCCGGCCCCCCATCTTacTAGAGAGACCTTCCTGGCG‑3′, 646-635-mu forward 5′-CTTTCCTTCCGAATCCGCCCCAtaTGTTCCACCCCGAGCTTCCCC‑3′, reverse 5′-GGGGAAGCTCGGGGTGGAACAtaTGGGGCGGATTCGGAAGGAAAG‑3′, 571-564-mu: forward 5′- GAGGAGCATCTACATAgtAAGAGGCTTAAACTGCC‑3′, reverse 5′-GGCAGTTTAAGCCTCTTacTATGTAGATGCTCCTC‑3′, 291-280-mu: forward 5′-ATTGGTGGACATGGCGCtcGCGCGTTTGCTCCGAC‑3′, reverse 5′-GGCAGTTTAAGCCTCTTacTATGTAGATGCTCCTC‑3′.

### Immunofluorescent staining

Cells were washed with PBS and fixed with methyl alcohol for 20 min at −20 °C, and then cells were blocked with PBS containing 3% bovine serum albumin (BSA) for 1 h at 37 °C. After being incubated with the primary antibody overnight at 4 °C, cells were treated with the secondary antibody for 1 h at 37 °C. Nuclei were stained with 4′,6-diamidino-2-phenylindole and IF photomicrographs were captured using a fluorescent microscope (Carl Zeiss, Germany).

### Clonogenic survival assays

The day before treatment, cells were seeded in 6-well plates at 500 or 1000 cells per well and three replicates per condition. After appropriate treatment for 5–7 days, cells were stained with crystal violet, and the number of colonies per well was counted.

### Lentivirus production and transduction

Lentiviral production and transduction were performed using a lentiviral vector with a lentiviral packaging mix. The transfection complex was added to 80% confluent HEK-293T cells in a 10-cm dish and incubated for 24 h followed instruction of ExFect transfection reagent (Vazyme Biotech, Nanjing, China) following the manufacturer’s instructions. The medium was replaced 24 h after transfection. Viral supernatant was harvested 48 h after transfection and stored at −80 °C. Transduction of cells was performed in suspension as follows: 15,000 cells and diluted virus were mixed in 100 μl of the cells medium containing 5 μg/ml polybrene (MCE, NJ, USA), incubated for 30 min at 37 °C in a well of a round-bottomed 96-well plate, plated onto a well of a feeder containing 96-well plate, and cultured until functional analyses.

### ChIP assay

ChIP assays were performed using the ChIP assay kit (P2078, Beyotime, Shanghai, China) according to the manufacturer’s instructions. The fragment of the human ATM promoter containing the E2-box element in immunoprecipitates was amplified by Q-PCR. The primers were forward 5′-CAGACAAATGCTCTTTCAGGGG-3′, reverse 5′-GCCTCTTTGTATGTAGATGCTC-3′.

### EMSA

Cells from a 10-cm dish were resuspended in PBS. The cytoplasmic and nuclear fraction were collected using nuclear and cytoplasmic protein extraction kit (P0028, Beyotime, Hangzhou, China). For analysis of ZEB1 DNA binding, EMSA was used and performed as previously described [49]. Double-stranded oligonucleotide probes were synthesized (Beyotime, Hangzhou, China) to contain the E-box site sequences from the promoters of the indicated target genes (ATM). The oligos were end labeled with biotin using biotin 3′end DNA labeling kit (GS008, Beyotime, Hangzhou, China). EMSA was performed by incubating 5 μg of nuclear extract with 4 nmol biotin-labeled DNA probe. Absence of nuclear extract, competition with 100-fold molar excess unlabeled DNA prob or non-specific oligo served as controls. Supershift studies were performed by pre-incubation with the specified antibodies for 30 min. All EMSA assays were performed by chemiluminescent EMSA kit (GS009, Beyotime, Hangzhou, China). Complexes were separated by electrophoresis on non-denaturing 6.5% acrylamide gel. Sequences of EMSA probes are provided as follow: E-box: forward 5′-TGGCATTTCACACCTCTACACTGGACG′, reverse 5′-CGTCCAGTGTAGAGGTGTGAAATGCCA-3′; Non-specific: forward 5′-TCGAGTTGATGTAACCGACTCAGGCACT′, reverse 5′-AGTGCCTGAGTCGGTTACATCAACTCGA-3′.

### Immunohistochemistry

Immunohistochemical stains were performed using IHC kit (Key-GEN, Nanjing, China). Briefly, paraffin-embedded slides were deparaffinized, rehydrated and washed in 1% PBS-Tween. The sections were then treated with 3% hydrogen peroxide and blocked with 10% goat serum for 1 h at 37 °C. Slides were incubated with primary antibodies in PBS containing 1% BSA (1:50) for 1 h at 37 °C. Biotinylated secondary anti-rabbit antibodies were added and incubated at room temperature for 1 h. Streptavidin-HRP was added, and after 40 min the sections were stained with 3,3′ diaminobenzidine substrate and counterstained with hematoxylin.

### Primary cell lines obtained from xenograft model

For the establishment of mU251, U251 cell suspension (1 × 10^6^ cells per mouse, 0.2 ml) was injected subcutaneously into the left armpit of nude mice and 10 nu-mice were inoculated. After 15 days, excised tumors were digested in collagenase/hyaluronidase and DNase I (Biosharp) and cultured for the establishment of mU251 cells. For mrU251 cells, the U251 tumors were treated with different doses of TMZ. Next, tumors were cleaned, digested in collagenase/hyaluronidase and DNase I (Biosharp) and cultured for establishment of mrU251 cells. Animal welfare and experimental procedures were performed in accordance with the Guide for the Care and Use of Laboratory Animals and the related ethical regulations of China pharmaceutical university. Pathogen-free BALB/c-nu mice were purchased from the Model Animal Research Center of Nanjing University (Nanjing, China)

### Statistical analysis

All experiments were performed with at least three independent replicates. Results are presented as the mean ± standard deviation. Statistical analyses were performed with GraphPad software using Student’s *t* test for two groups or one-way ANOVA for multiple groups. *p* values <0.05 were considered significant.

## Supplementary information

Supplementary Figure legends and tables

Supplementary Figure S1

Supplementary Figure S2

Supplementary Figure S3

Supplementary Figure S4

Supplementary Figure S5

Supplementary Figure S6

Supplementary Figure S7

Supplementary Figure S8

Supplementary Figure S9

Supplementary Figure S10

aj-checklist

Original Data of WB blots

## Data Availability

All western blots presented in figures were given in the section of Supplementary Materials which were named Original Data of WB blots. The data that support the findings of this study are available from the corresponding author, upon reasonable request.
