# Supplementary material for: Acquired temozolomide resistance in MGMTlow gliomas is associated with regulation of homologous recombination repair by ROCK2
Source: Cell Death Dis. 2022 Feb 10;13(2):138. doi: 10.1038/s41419-022-04590-6 (PMC8831658; doi:10.1038/s41419-022-04590-6)
Supplement: Supplementary file 1 — Supplementary Figure legends and tables [file 41419_2022_4590_MOESM1_ESM.docx]

**Supplementary Figure Legends**

**Supplementary Figure S1. The features of TMZ-R cells.** The viability of resistance cells and parental cells after treatment with TMZ. (A) U87/U87R, (B) U251/U251R, (C) A172/A172R, (D) U138/U183R, (E) U118/U118R, (F) T98G/T98GR. Cell were treated with various concentrations of TMZ (0, 50, 100, 500, 1000, or 2000 μM) for 48 h, and then cell viabilities were determined by the MTT assay. (G) ROCK2 mRNA expression in cells. (H) MGMT mRNA expression in cells. (I, J, K, L, M and N) Bliss synergism analysis of cells treated with varying doses of fasudil and TMZ. (O, P and Q) TMZ showed an increased cytotoxicity with fasudil in MGMT^low^ TMZ-R cells. (R) p-ROCK2, γH2X expression with fasudil. Data are shown as mean ± SD of three independent experiments. P values determined by ANOVA or two-tailed unpaired t-test. Statistical differences compared with the controls are given as *P < 0.05, **P < 0.01, ***P < 0.005, ****P < 0.001.

**Supplementary Figure S2. Primary cells were obtained from U251 xenograft tumor model. (A)** U251 cells was used to establish a xenograft tumor, followed by a sequential treatment of with TMZ at different doses. After 4 four phases, a TMZ-resistant xenograft tumor is established, and tumor tissue was isolated from the xenograft tumor and disseminated into single cells until growing at a rate of exponential proliferation, in vitro. The cell was named mrU251.(B) Primary cells were obtained from U251 xenograft tumor model. This cell was named as mU251. Cell were treated with various concentrations of TMZ (0, 50, 100, 500, 1000, or 2000 μM) for 48 h, and then cell viabilities were determined by the MTT assay (C and D). (E) Clone formation with TMZ (100μM). Data are mean ± SD. P values determined by ANOVA or two-tailed unpaired t-test. Statistical differences compared with the controls are given as *P < 0.05, **P < 0.01, ***P < 0.005, ****P < 0.001.

**Supplementary Figure S3. HR repair in TMZ-R cells.** Efficiency of HR (A), NHEJ (B), SSA (C), a-EJ (D) repair in TMZ-R cells compared with parental cells. Data are mean ± SD of three independent experiments. (E, F, G, H) DSB–ChIP quantification over time of γH2AX, MRE11, ATM, BRCA1, PRA2 and RAD51 levels at the site-specific DSB. P values determined by ANOVA or two-tailed unpaired t-test. Statistical differences compared with the controls are given as *P < 0.05, **P < 0.01, ***P < 0.005, ****P < 0.001.

**Supplementary Figure S4. DSB–ChIP quantification of HR factors.** DSB–ChIP quantification over time of MRE11, ATM, BRCA1, PRA2 and RAD51 levels at the site-specific DSB with ROCK2-KD or fasudil. U87R cells: (A) MRE11. (B) ATM. (C) BRCA1. (D) RPA2. (E) RAD51. (F) IgG control. U251R cells: (G) MRE11. (H) ATM. (I) BRCA1. (J) RPA2. (K) RAD51. (L) IgG control. A172R cells: (M) MRE11. (O) ATM. (P) BRCA1. (Q) RPA2. (R) RAD51. (S) IgG control. mrU251 cells: (T) MRE11. (U) ATM. (V) BRCA1. (W) RPA2. (X) RAD51. (Y) IgG control. Data are mean ± SD of three independent experiments. P values determined by ANOVA or two-tailed unpaired t-test. Statistical differences compared with the controls are given as *P < 0.05, **P < 0.01.

**Supplementary Figure S6. ROCK2 did not regulated MRE11 expression.** (A) Co-expression analyze of MRE11A expression with ROCK2 expression using CGGA data. (B) Gene expressions of MRE11 was determined with ROCK2-KD. (C and D) MRE11 protein level was determined with ROCK2-KD. Data are presented as mean ± SD of three independent experiments. P values determined by ANOVA or two-tailed unpaired t-test. Statistical differences compared with the controls are given as *P < 0.05, **P < 0.01.

**Supplementary Figure S6. ROCK2 did not regulated other HR factors.** Co-expression of KAT5 (A), BRCA1 (B), RAD51 (C), RPA2 (D) with ROCK2 using the mRNAseq_693 data set from the CGGA. (E, F, G and H) Gene expression of HR factors were determined in U87R/U251/A172R/mrU251R cells with ROCK2-KD. Data are shown as mean ± SD of three independent experiments. P values determined by ANOVA or two-tailed unpaired t-test. Statistical differences compared with the controls are given as *P < 0.05, **P < 0.01.

**Supplementary Figure S7. ATM expression in slides and tumor samples of animal study with fasudil + TMZ therapy. The slides and tumor samples were collected in the research we reported before.** (A) γH2AX and ATM expression were test in vivo. For U251R assay: TMZ (25mg/kg), fasudil (20mg/kg), TMZ (25mg/kg) plus fasudil (20mg/kg). (B) Scores of IHC. (C) Percentage of positive stained cell. (D) ROCK2, p-ROCK2, ATM expression were determined in samples from U251 xenograft tumors. P values determined by ANOVA or two-tailed unpaired t-test. Statistical differences compared with the controls are given as*P < 0.05, **P < 0.01, ***P < 0.005, ****P < 0.001.

**Supplementary Figure S8.** **ZEB1 bond to motif for ATM regulation.** (A) ChIP assay of ATM promoter in U87/U87R and U251/U251R cells. (B) Luciferase reporter assay of ATM promoter in U87/U87R and U251/U251R cells. (C) Computational prediction of transcription-factor binding site locations by JASPAR (http://jaspar.genereg.net/analysis). (D) Motif in 0.9k. (E) ATM gene expression was determined with promotor mutation plasmids in TMZ-R cells. (F) EMSA assay of ZEB1 combined with probes contained E2-box. Competition with specific (SC) or nonspecific (NS) probe results were showed in the right panel. (G) Extract luciferase activities were determined 36 h after transfection. Luciferase were detected. (H) ATM protein expression was determined with promotor mutation plasmids. (I and J) ATM expression was tested with promotor mutation plasmids in ROCK2-KD cells. (K and L) ATM expression was tested with promotor mutation plasmids in ROCK2-OE cells. Data are shown as mean ± SD of three independent experiments. P values determined by ANOVA or two-tailed unpaired t-test. Statistical differences compared with the controls are given as *P < 0.05, **P < 0.01, ***P < 0.005, ****P < 0.001.

**Supplementary Figure S9. Determination of transcription cofactors for ZEB1.** (A) ChIP assay of ATM promoter with Smad3-siRNA. Expression of ATM with Smad3-siRNA. (B) ChIP assay of ATM promoter with PCAF-siRNA. Expression of ATM with PCAF-siRNA. (C) ChIP assay of ATM promoter with LEF1-siRNA. Expression of ATM with LEF1-siRNA. (D) Quantification of Fig. 8A. (E and F) Expression of K27me3, K4me3 and K79me2 of Histone 3 were texted in nucleus. (G, H and I) ChIP assay of ATM promoter with K27me3, K4me3 and K79me2 of Histone 3. Data are shown as mean ± SD of three independent experiments. P values determined by ANOVA or two-tailed unpaired t-test. Statistical differences compared with the controls are given as *P < 0.05, **P < 0.01, ***P < 0.005, ****P < 0.001.

**Supplementary Figure S10.** Quantification of Fig. 8B, 8C, 8F, 8H, 8K and 8L. (A) Quantification of WB of input in Fig. 8B. (B and C) Quantification of WB in Fig. 8C. (D and E) Quantification of WB in Fig. 8H. (F and G) Quantification of WB in Fig. 8K. (H and I) Quantification of WB in Fig. 8L. Data are shown as mean ± SD of three independent experiments. P values determined by ANOVA or two-tailed unpaired t-test. Statistical differences compared with the controls are given as *P < 0.05, **P < 0.01, ***P < 0.005, ****P < 0.001.

**Supplementary table 1. The sequences of primers for Q-PCR**

| Gene name | F | R |
| --- | --- | --- |
| ATM | ATCTGCTGCCGTCAACTAGAA | GATCTCGAATCAGGCGCTTAAA |
| GAPDH | AAGGTCGGAGTCAACGGATTT | AGATGATGACCCTTTTGGCTC |

**Supplementary table 2. The sequences of siRNA**

| Gene name | sense | anti-sense |
| --- | --- | --- |
| ATF-2 | UAAACAAACCCACUUCUUCAC | GAAGAAGUGGGUUUGUUUAAU |
| GR | UCUUCGAAUUUUAUCGAUGAU | CAUCGAUAAAAUUCGAAGAAA |
| WT1 | AUUUGGUAUAAAUUGUCACUG | GUGACAAUUUAUACCAAAUGA |
| POU2F2 | UAUUUUGGGGGUUCUGAUGAU | CAUCAGAACCCCCAAAAUAAG |
| RFX1 | ACAGAAAAGGUUCCAUUACAA | GUAAUGGAACCUUUUCUGUGC |
| CTF | AUUUUCCACCGAAAACGUGGG | CACGUUUUCGGUGGAAAAUUA |
| NF-KB | UCCUUAUCUCUAAAUGUCCCU | GGACAUUUAGAGAUAAGGACA |
| ZEB1 | UUAUGGAUUGGAAGGAAACAU | GUUUCCUUCCAAUCCAUAAUU |
| TBP | AAUAGACAGACUAUUGGUGUU | CACCAAUAGUCUGUCUAUUUU |
| AP-2 | UUUUCUCUUAAAGAUCUUCCU | GAAGAUCUUUAAGAGAAAAAC |
| SRF | AUACUCAUGGCAAACAUCGAG | CGAUGUUUGCCAUGAGUAUUA |
| E2F2 | UAAUCUGACCCUUCUUUACAG | GUAAAGAAGGGUCAGAUUAAA |
| SP1 | UUGAUACUGAAUAUUAGGCAU | GCCUAAUAUUCAGUAUCAAGU |
| RXR-a | AUUGGUAAUACUACUACAGGU | CUGUAGUAGUAUUACCAAUCC |
| P53 | ACAAGAAGUGGAGAAUGUCAG | GACAUUCUCCACUUCUUGUUC |
| Smad3 | GGCAAGUCAUACAGCUCAAAU | UUGAGCUGUAUGACUUGCCAA |
| PCAF | GAGGAGUCUUGUAAAUGUAAU | UACAUUUACAAGACUCCUCGG |
| LEF1 | CGAAGAGGAAGGCGAUUUAGC | UAAAUCGCCUUCCUCUUCGGG |
| YAP1 | GGUUGAUCACUCAUAAUAAUU | UUAUUAUGAGUGAUCAACCUA |

**Supplementary table 3. The sequences of shRNA**

| Gene name | / | Clone ID | Sequence |
| --- | --- | --- | --- |
| ROCK2 | shRNA1 | NM_004850.3-5578s1c1 | CCTTGATGTCTGTCTATTATTCTCGAGAATAATAGACAGACATCAAGG |
|  | shRNA2 | NM_004850.3-922s1c1 | CCTTTCAAGATGATAGGTATCCTCGAGGATACCTATCATCTTGAAAGG |
| ZEB1 | shRNA1 | NM_030751.4-1268s21c1 | TGTCTCCCATAAGTATCAATTCTCGAGAATTGATACTTATGGGAGACA |
|  | shRNA2 | NM_030751.2-572s1c1 | CCTCTCTGAAAGAACACATTACTCGAGTAATGTGTTCTTTCAGAGAGG |
| YAP1 | shRNA1 | NM_006106.2-1928s1c1 | CCCAGTTAAATGTTCACCAATCTCGAGATTGGTGAACATTTAACTGGG |
| ATM | shRNA1 | NM_000051.3-4332s1c1 | CCAAGGTCTATGATATGCTTACTCGAGTAAGCATATCATAGACCTTGGT |
|  | shRNA2 | NM_000051.x-9530s1c1 | TGATGGTCTTAAGGAACATCTCTCGAGAGATGTTCCTTAAGACCATCA |
| MRE11 | shRNA1 | NM_005591.3-1038s21c1 | TGTTGGTTTGCTGCGTATTAACTCGAGTTAATACGCAGCAAACCAACA |
|  | shRNA2 | NM_005591.3-1233s21c1 | ACGGGAACGTCTGGGTAATTCCTCGAGGAATTACCCAGACGTTCCCG |
| BRCA1 | shRNA1 | NM_007294.x-1481s1c1 | AGAATCCTAGAGATACTGAACTCGAGTTCAGTATCTCTAGGATTCTC |
|  | shRNA2 | NM_007294.x-7048s1c1 | TATAAGACCTCTGGCATGAATCTCGAGATTCATGCCAGAGGTCTTATA |
| Ku80 | shRNA1 | NM_021141.3-185s21c1 | CGTGGGCTTTACCATGAGTAACTCGAGTTACTCATGGTAAAGCCCACG |
|  | shRNA2 | NM_021141.3-2204s21c1 | AGAGGAAGCCTCTGGAAGTTCCTCGAGGAACTTCCAGAGGCTTCCTCT |
| Control | shRNA | / | CAACAAGATGAAGAGCACCAACTCGAGTTGGTGCTCTTCATCTTGTTG |

**Supplementary table 4. The primers for full-length PCR**

| Gene name | NM ID | F | R |
| --- | --- | --- | --- |
| ROCK2 | LT978485.1 | atgagccggcccccgccgacggggaaa | ttagctaggtttgtttggggcaagct |
| BRCA1 | NM_007300.4 | atggatttatctgctcttcgcgttgaa | tcagtagtggctgtgggggatctggggt |
| MRE11 | NM_005591.4 | atgagtactgcagatgcacttgatga | ttatcttctatttcttcttaaagaactagtgttc |
| ATM | NM_001351834.2 | ttatcttctatttcttcttaaagaactagtgttc | ttatcttctatttcttcttaaagaactagtgttc |
| RAD51 | D14134.1 | atggcaatgcagatgcagcttgaagcaa | gatggagtgggagatgccaaagactga |
| YAP1 | NM_001282101.2 | atggatcccgggcagcagccgccgcc | ctataaccatgtaagaaagctttctttatctag |
| Ku80 | NM_021141.4 | atggtgcggtcggggaataaggcag | ctatatcatgtccaataaatcgtccac |
