# Supplementary figures and images for: Acquired temozolomide resistance in MGMTlow gliomas is associated with regulation of homologous recombination repair by ROCK2
Source: Cell Death Dis. 2022 Feb 10;13(2):138. doi: 10.1038/s41419-022-04590-6 (PMC8831658; doi:10.1038/s41419-022-04590-6)

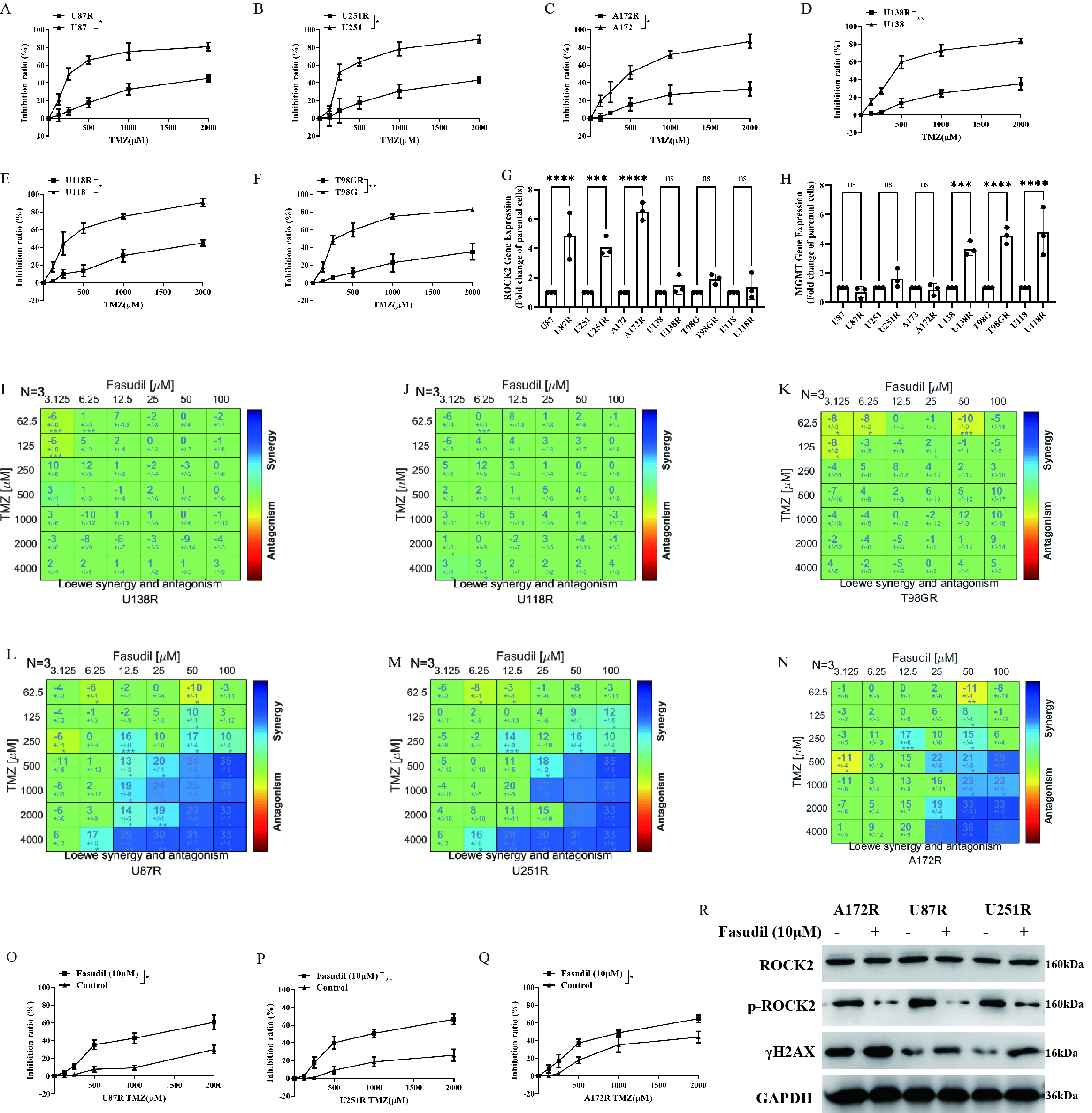

Supplement: Supplementary file 2 — Supplementary Figure S1 [file 41419_2022_4590_MOESM2_ESM.jpg]

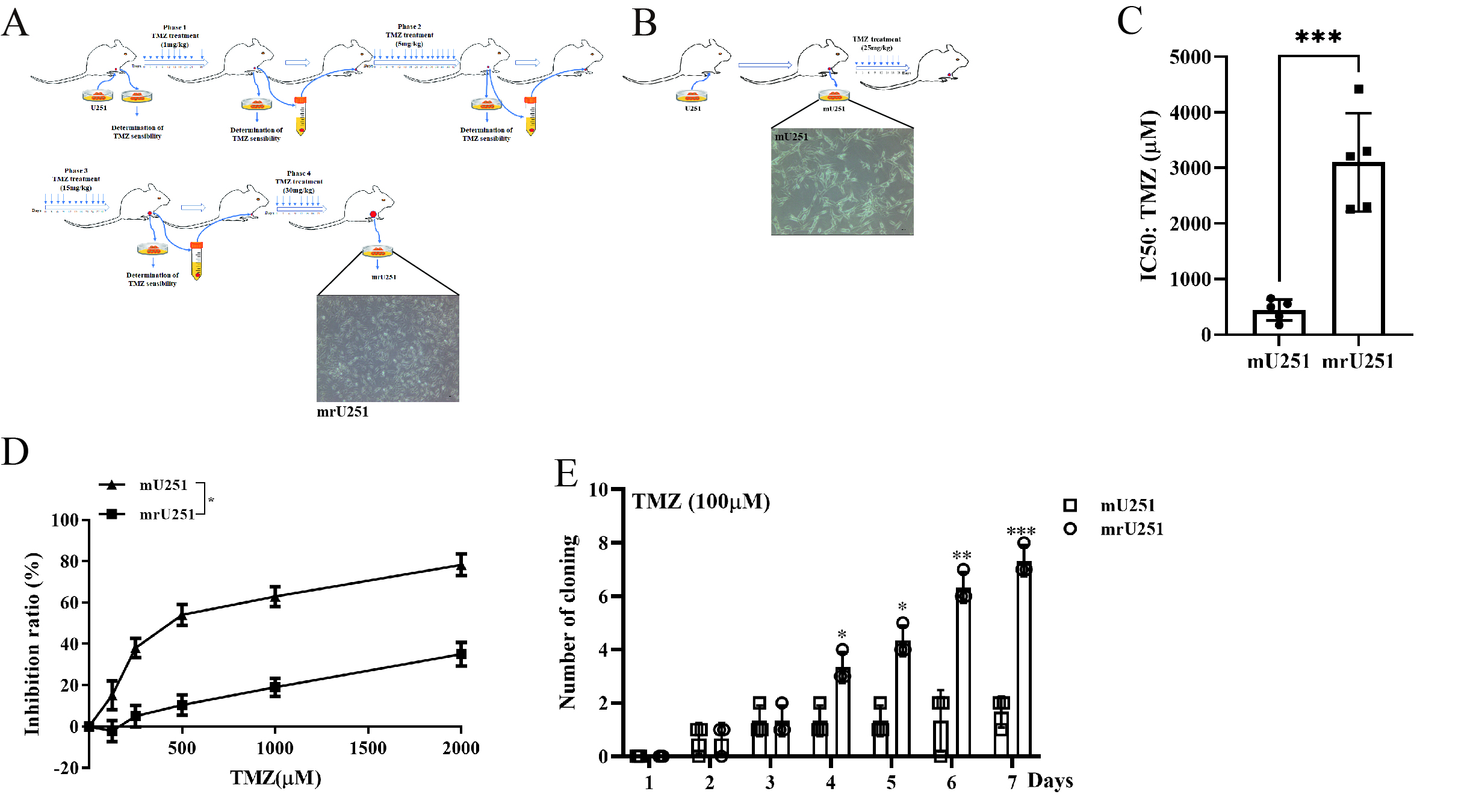

Supplement: Supplementary file 3 — Supplementary Figure S2 [file 41419_2022_4590_MOESM3_ESM.jpg]

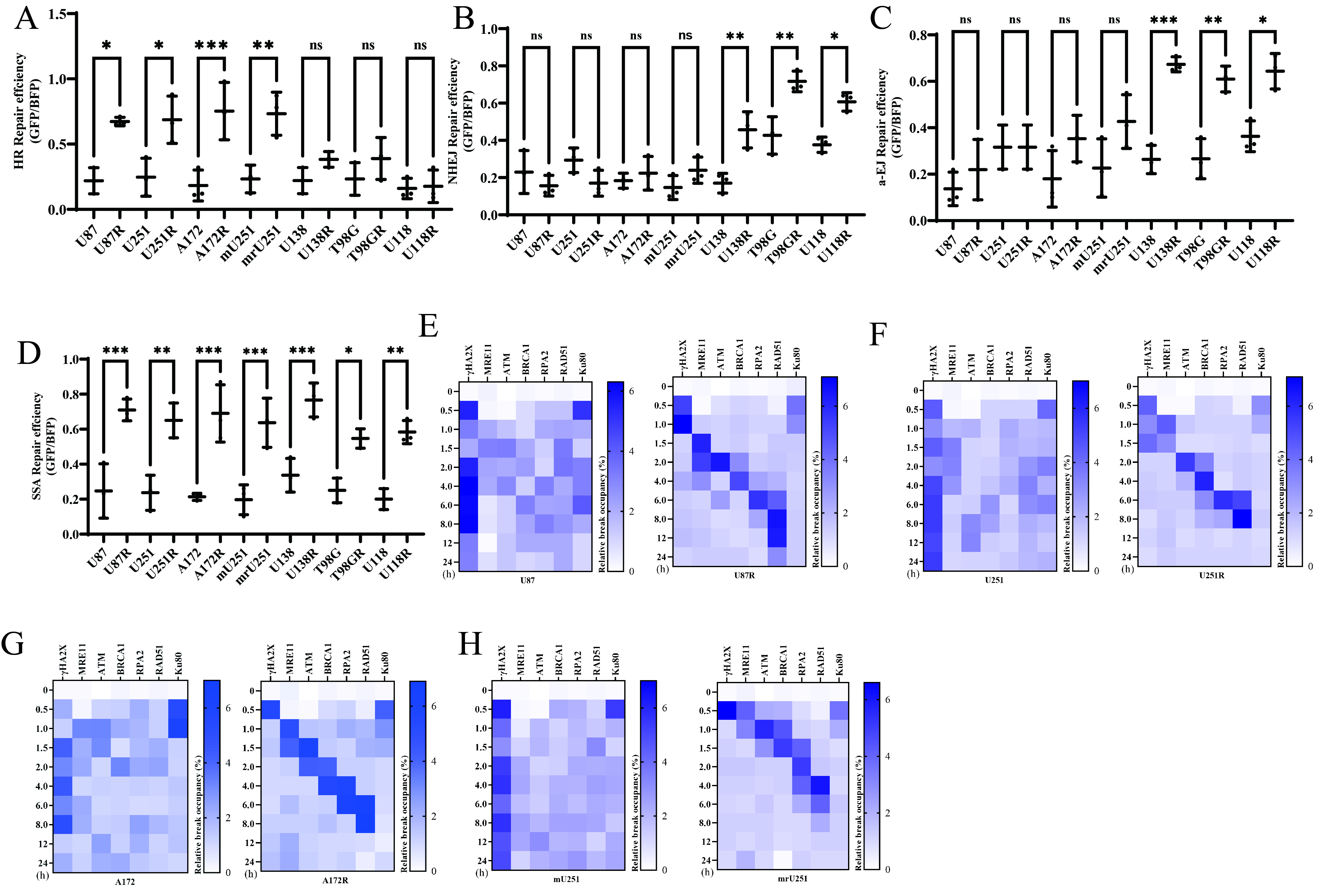

Supplement: Supplementary file 4 — Supplementary Figure S3 [file 41419_2022_4590_MOESM4_ESM.jpg]

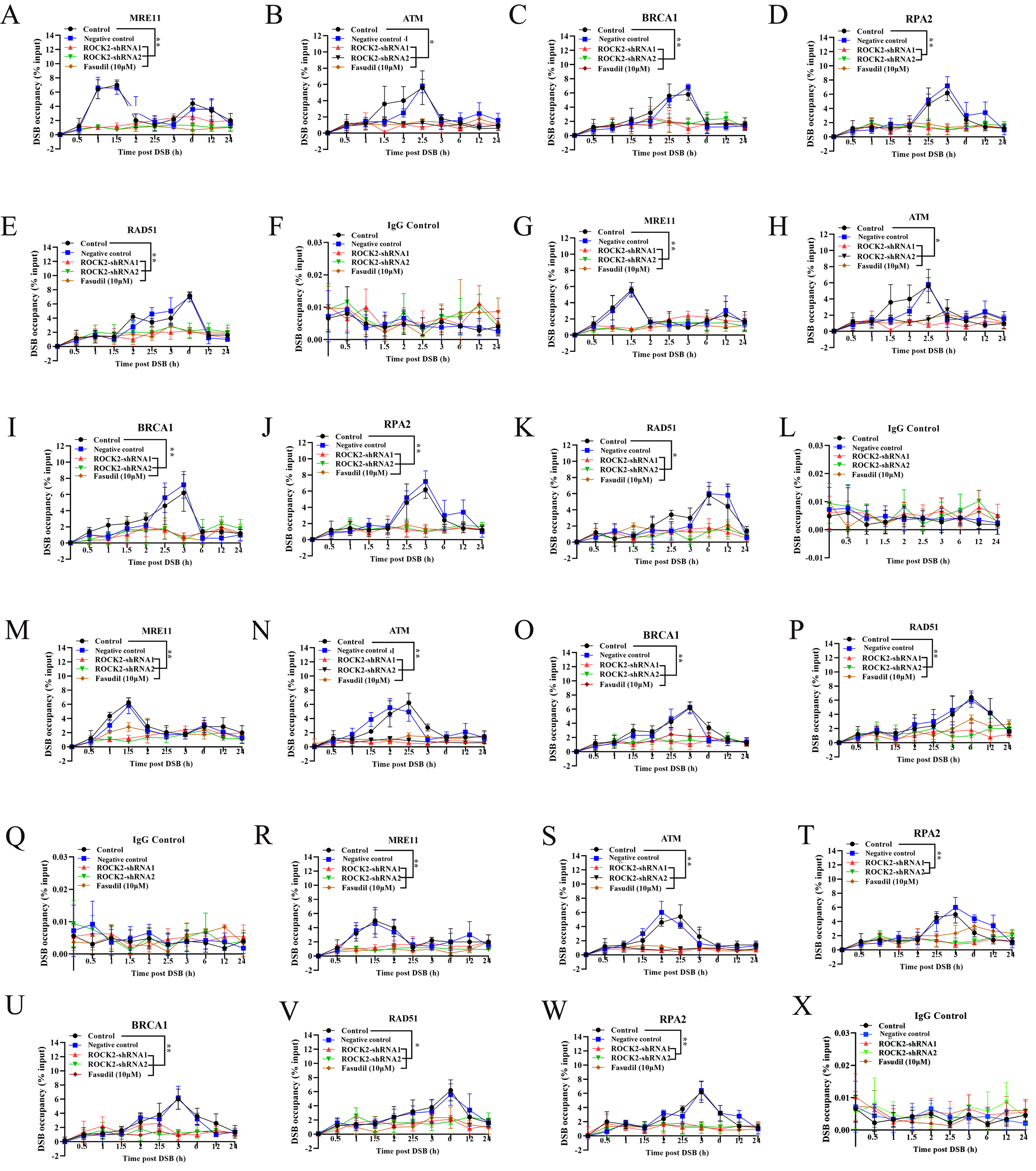

Supplement: Supplementary file 5 — Supplementary Figure S4 [file 41419_2022_4590_MOESM5_ESM.tif]

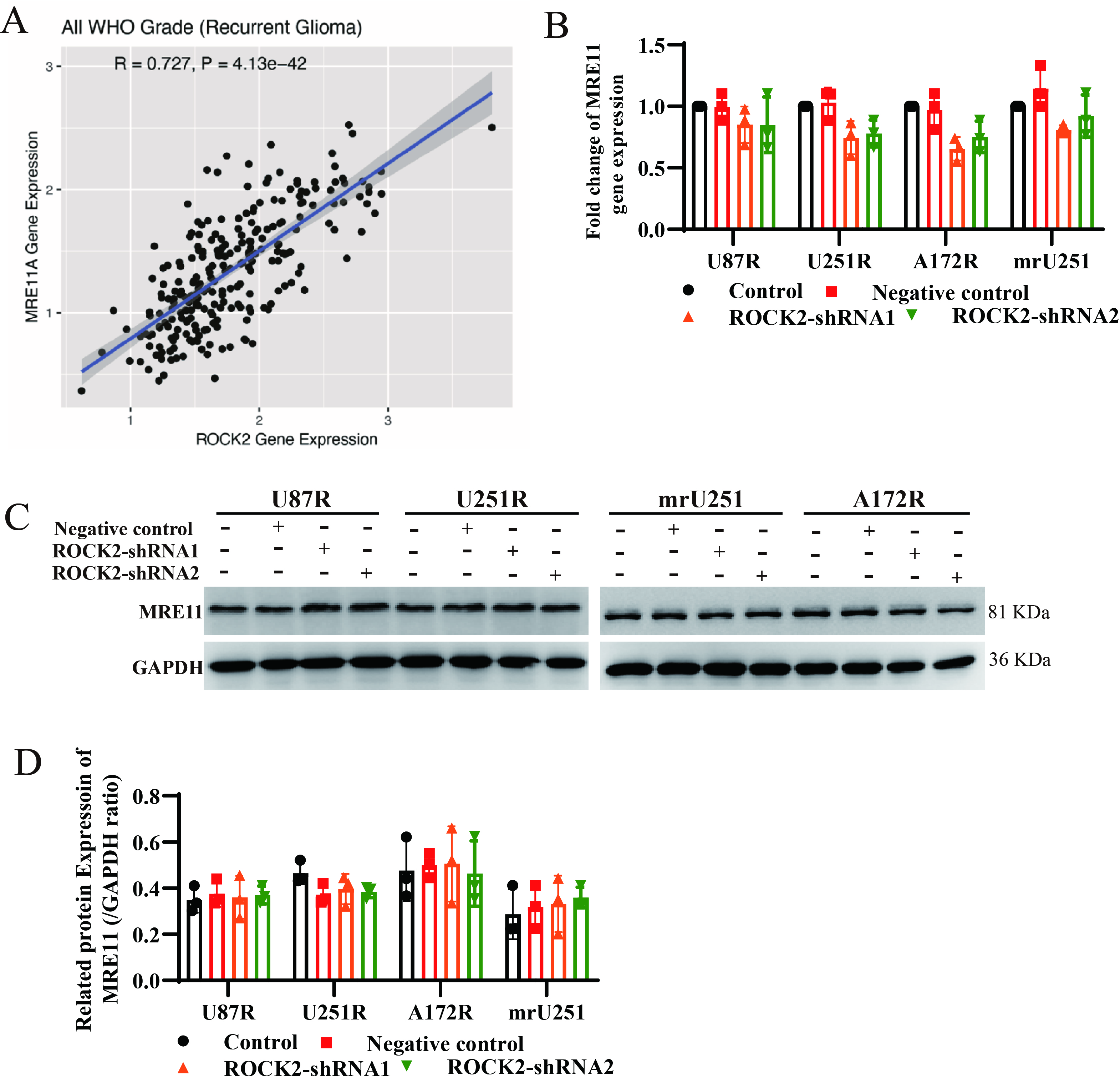

Supplement: Supplementary file 6 — Supplementary Figure S5 [file 41419_2022_4590_MOESM6_ESM.jpg]

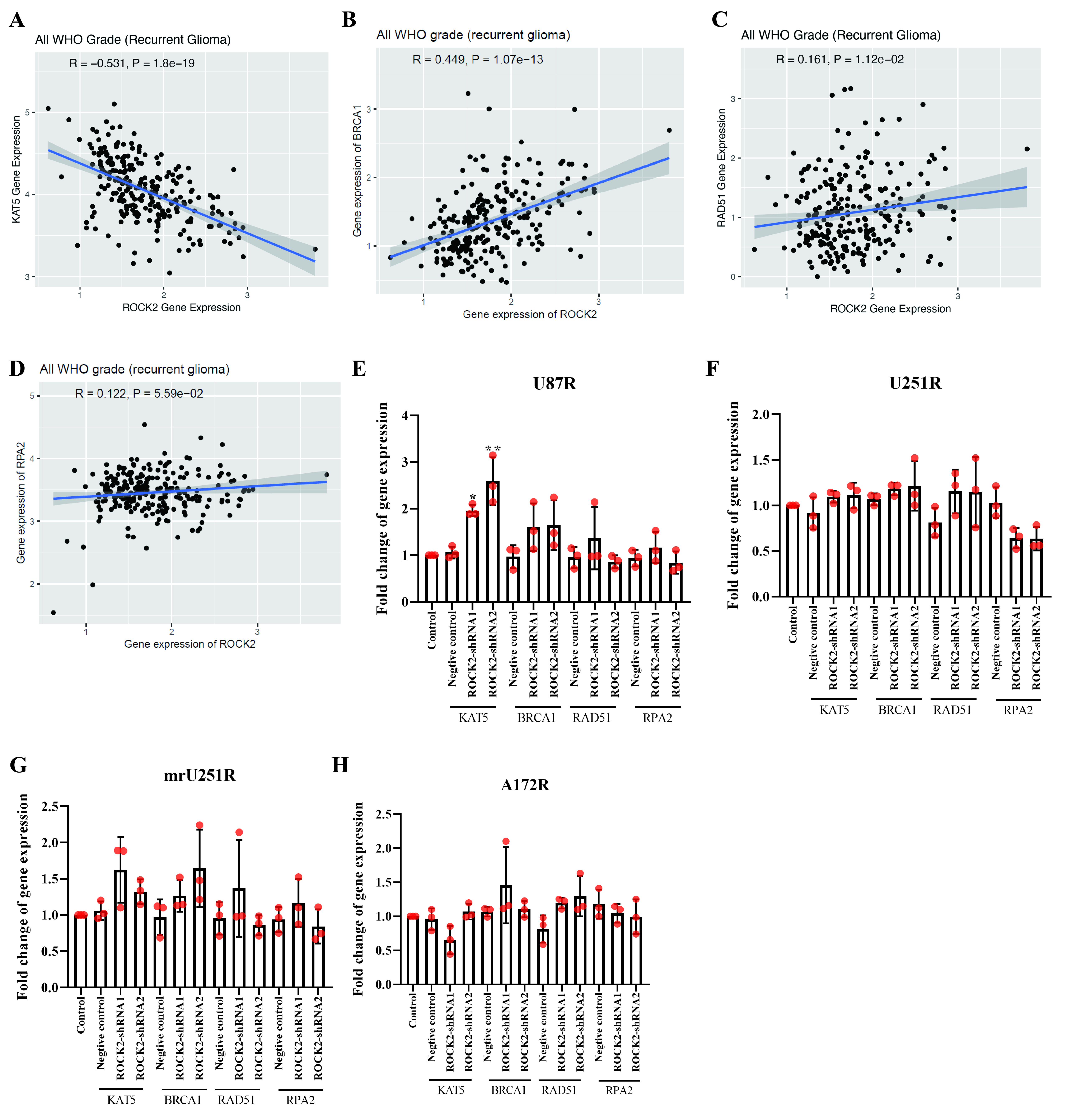

Supplement: Supplementary file 7 — Supplementary Figure S6 [file 41419_2022_4590_MOESM7_ESM.jpg]

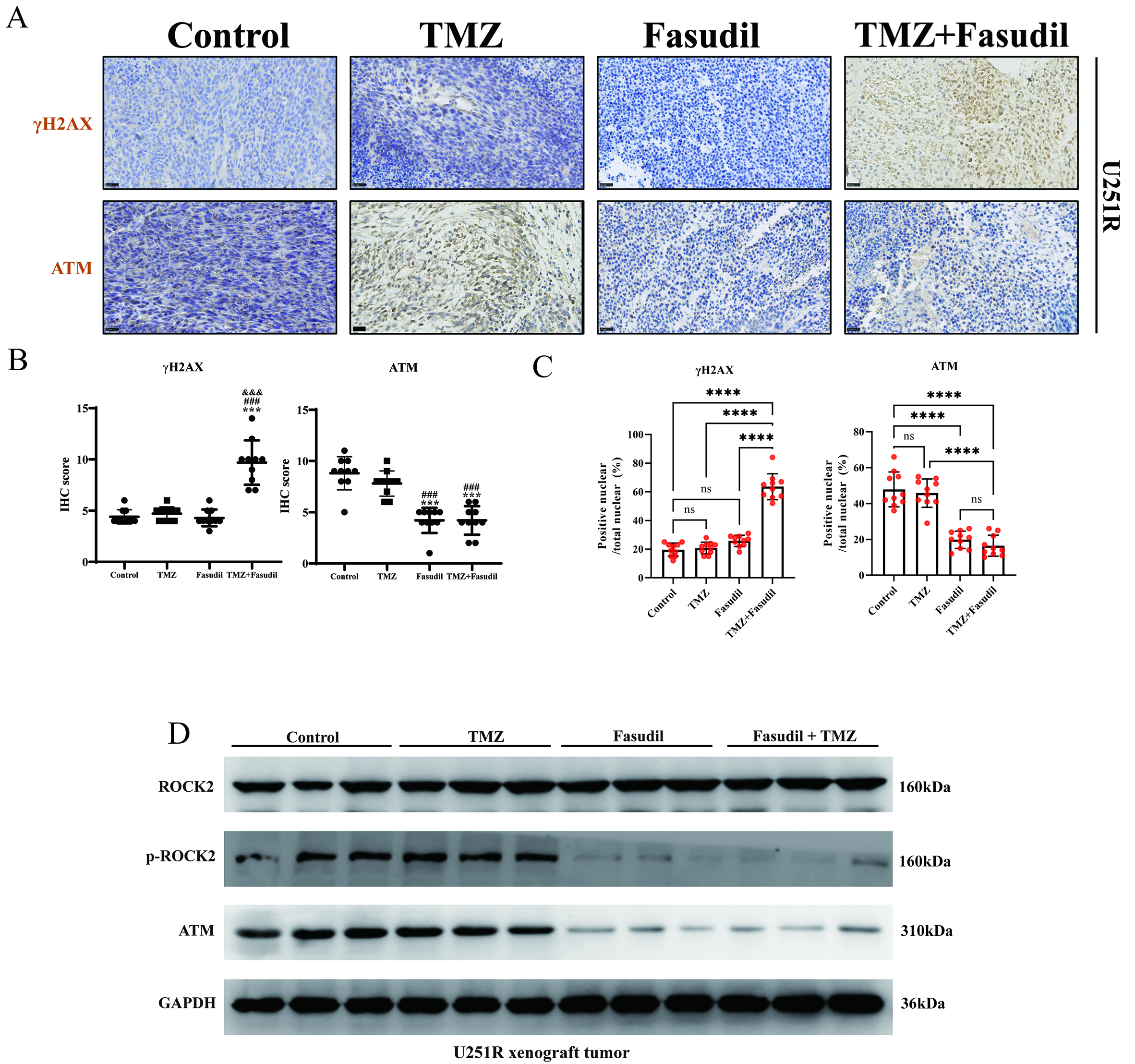

Supplement: Supplementary file 8 — Supplementary Figure S7 [file 41419_2022_4590_MOESM8_ESM.tif]

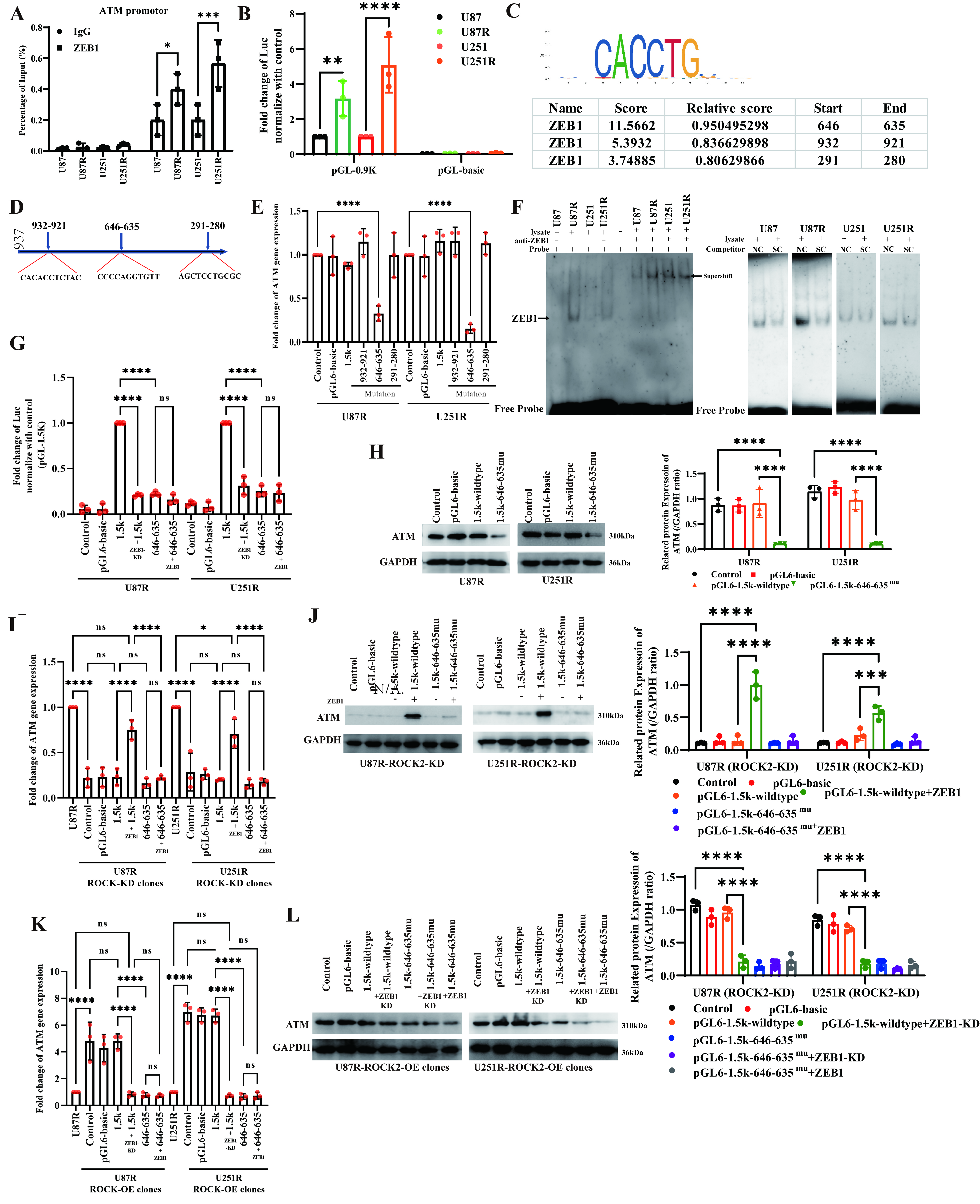

Supplement: Supplementary file 9 — Supplementary Figure S8 [file 41419_2022_4590_MOESM9_ESM.jpg]

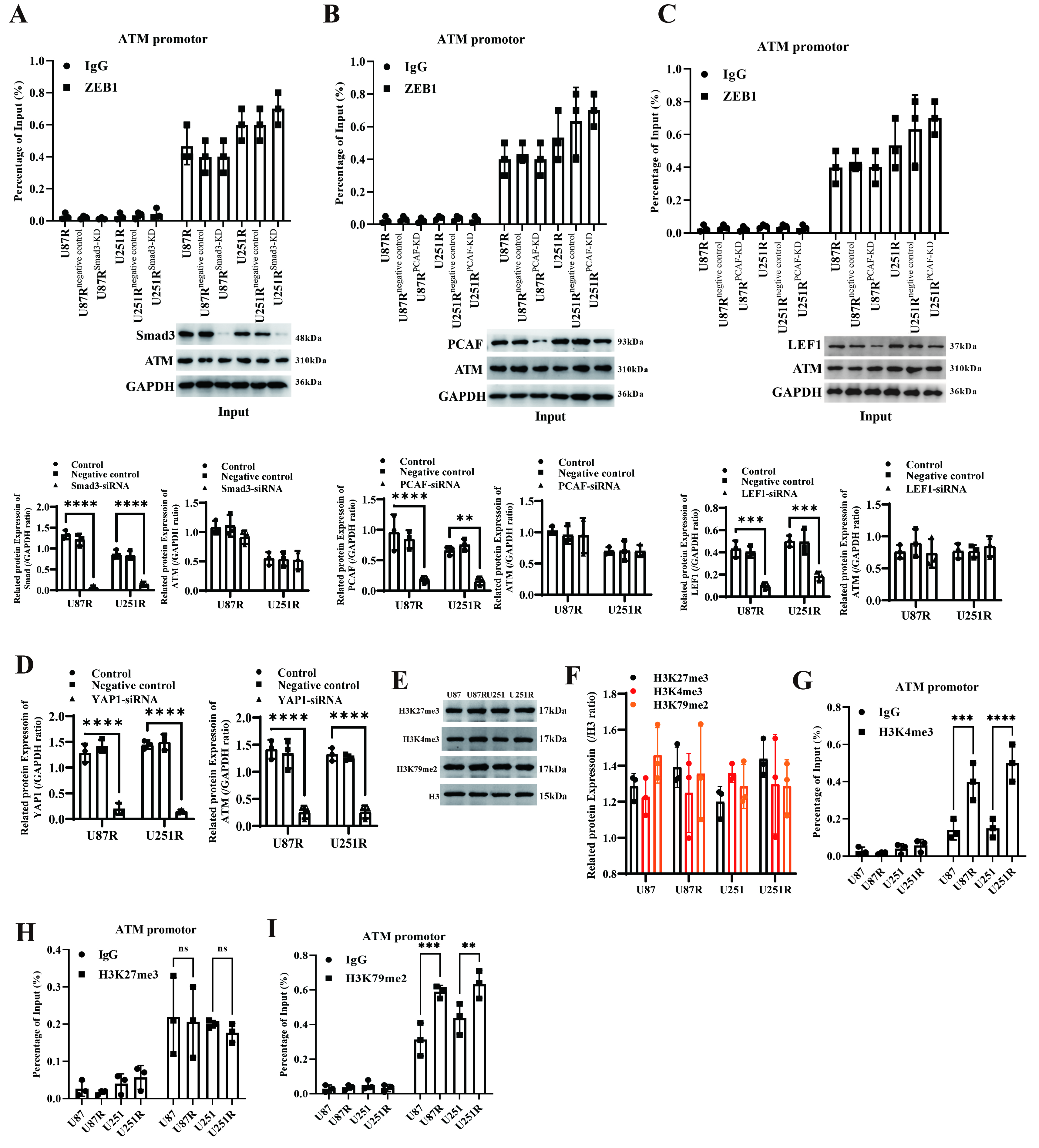

Supplement: Supplementary file 10 — Supplementary Figure S9 [file 41419_2022_4590_MOESM10_ESM.jpg]

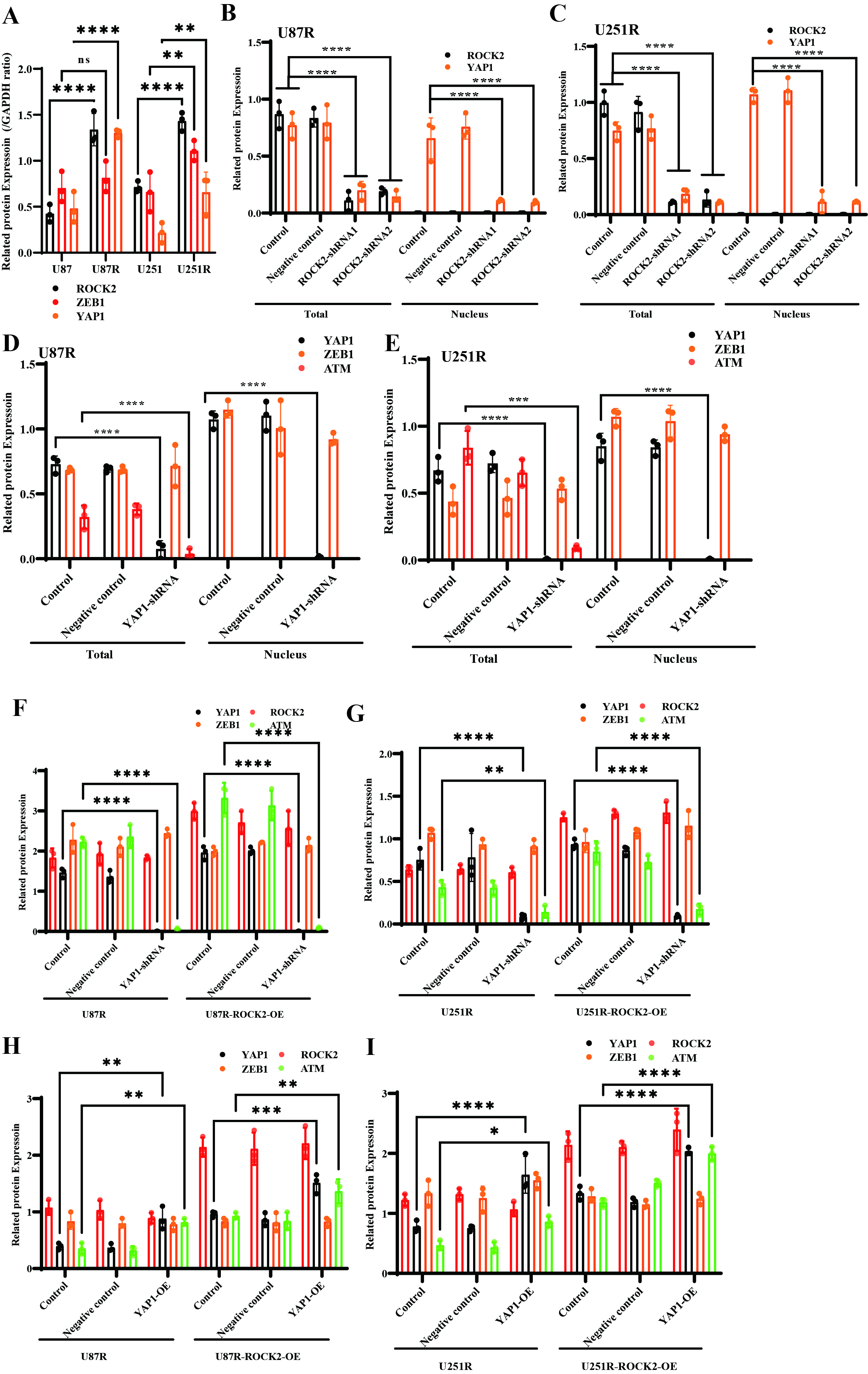

Supplement: Supplementary file 11 — Supplementary Figure S10 [file 41419_2022_4590_MOESM11_ESM.jpg]
