# Supplementary material for: Acquired temozolomide resistance in MGMTlow gliomas is associated with regulation of homologous recombination repair by ROCK2
Source: Cell Death Dis. 2022 Feb 10;13(2):138. doi: 10.1038/s41419-022-04590-6 (PMC8831658; doi:10.1038/s41419-022-04590-6)

The original data of WB blots

Fig.2A

Lane： 1 2 3 4

U87 U87R U251 U251R

ROCK2

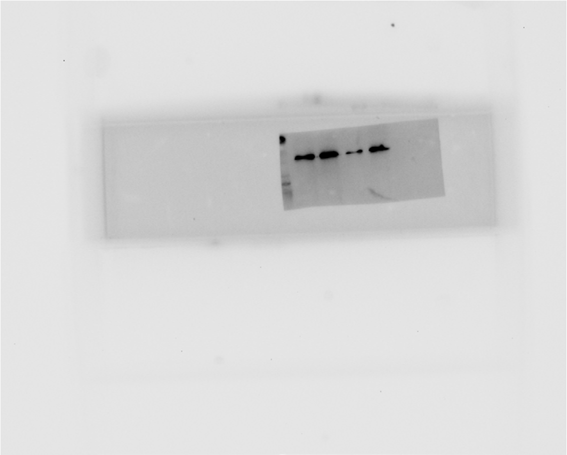

160kDa

GAPDH

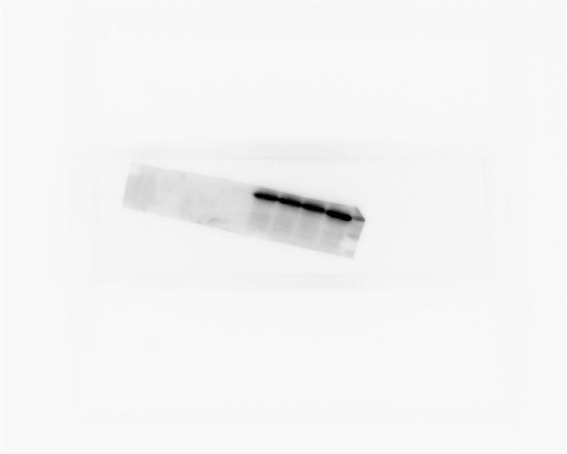

36kDa

Lane： 1 2 3 4

A172 A172R mU251 mrU251

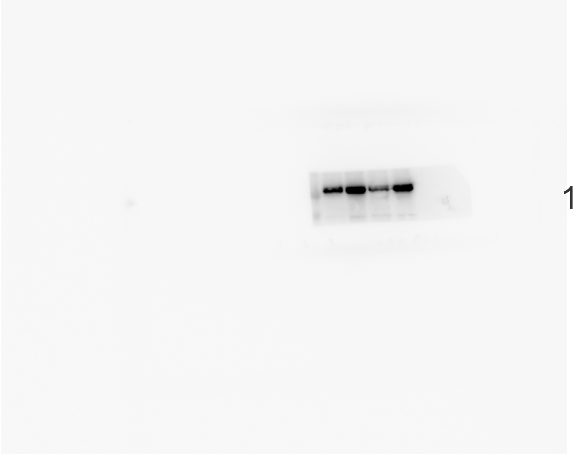

160kDa

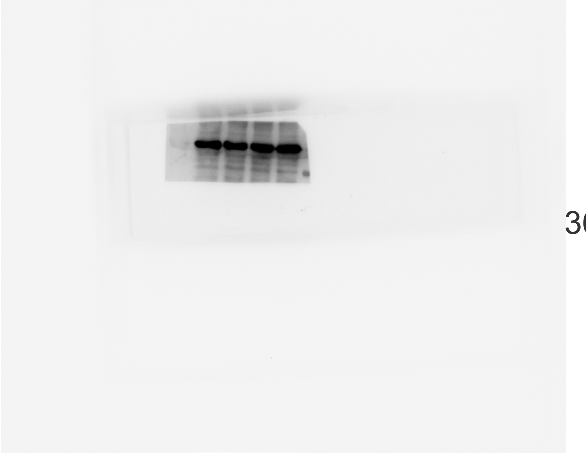

36kDa

Fig.2
B

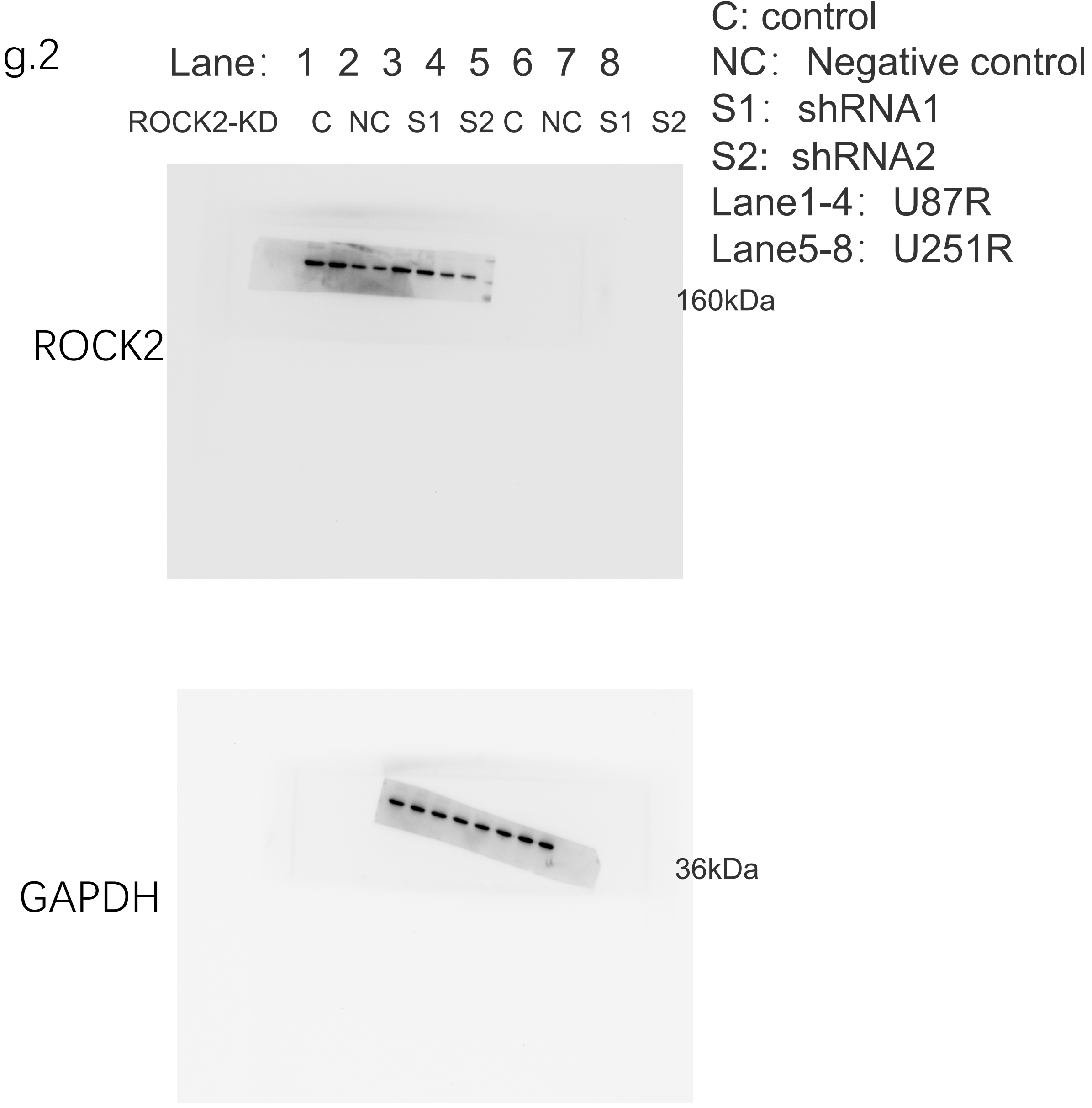
Lane： 1 2 3 4 5 6 7 8
ROCK2-KD C NC S1 S2 C NC S1 S2
C: control
NC： Negative control
S1： shRNA1
S2: shRNA2
Lane1-4： U87R
Lane5-8： U251R
ROCK2
160kDa
GAPDH
36kDa

Fig.2
C

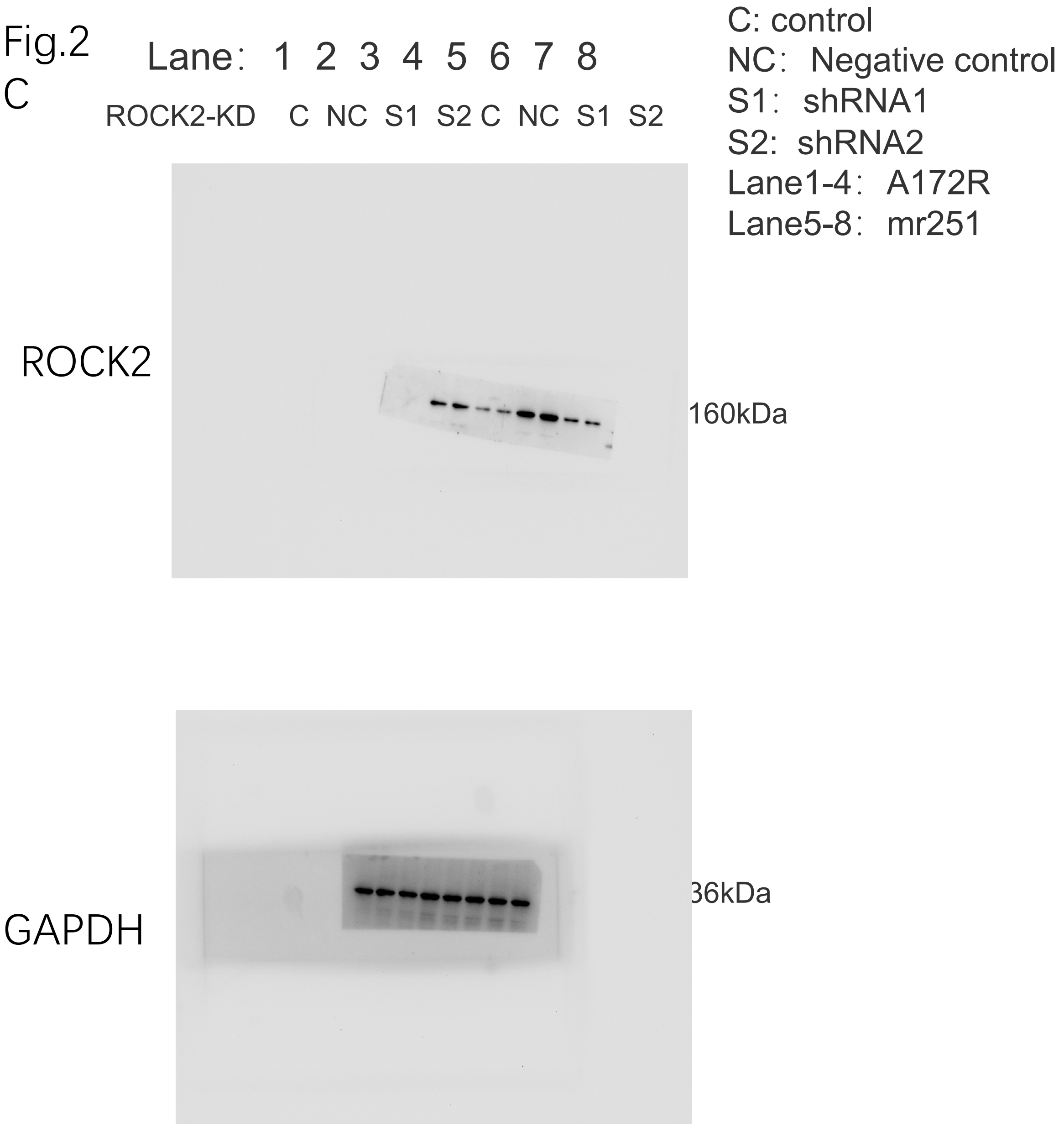
Lane： 1 2 3 4 5 6 7 8
ROCK2-KD C NC S1 S2 C NC S1 S2
C: control
NC： Negative control
S1： shRNA1
S2: shRNA2
Lane1-4： A172R
Lane5-8： mr251
ROCK2
160kDa
GAPDH
36kDa

Fig.2

J

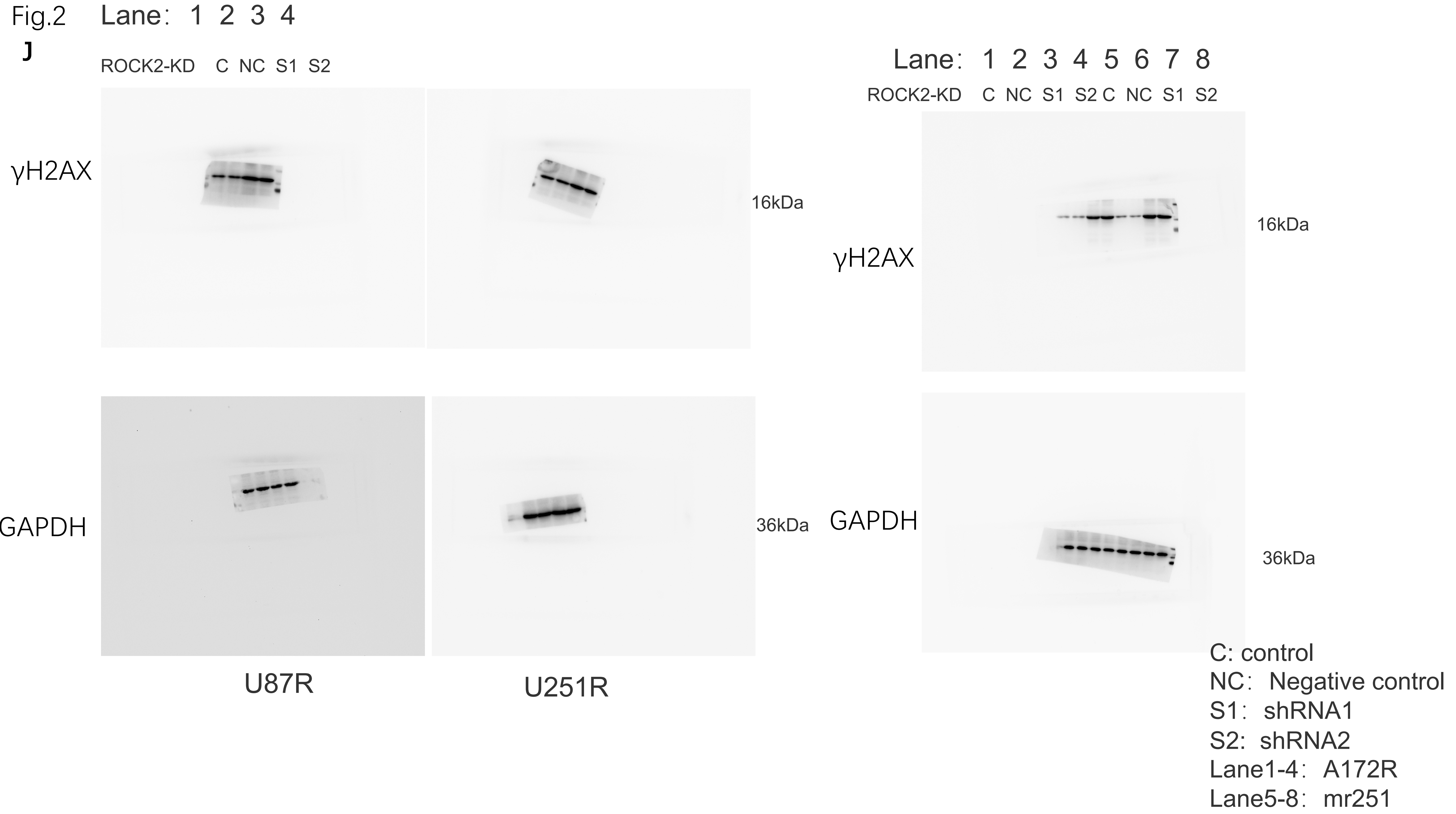
Lane: 1 2 3 4
ROCK2-KD C NC S1 S2
γH2AX
GAPDH
16kDa
36kDa
U87R
U251R
Lane: 1 2 3 4 5 6 7 8
ROCK2-KD C NC S1 S2 C NC S1 S2
γH2AX
GAPDH
16kDa
36kDa
C: control
NC: Negative control
S1: shRNA1
S2: shRNA2
Lane1-4: A172R
Lane5-8: mr251

Fig.3
F

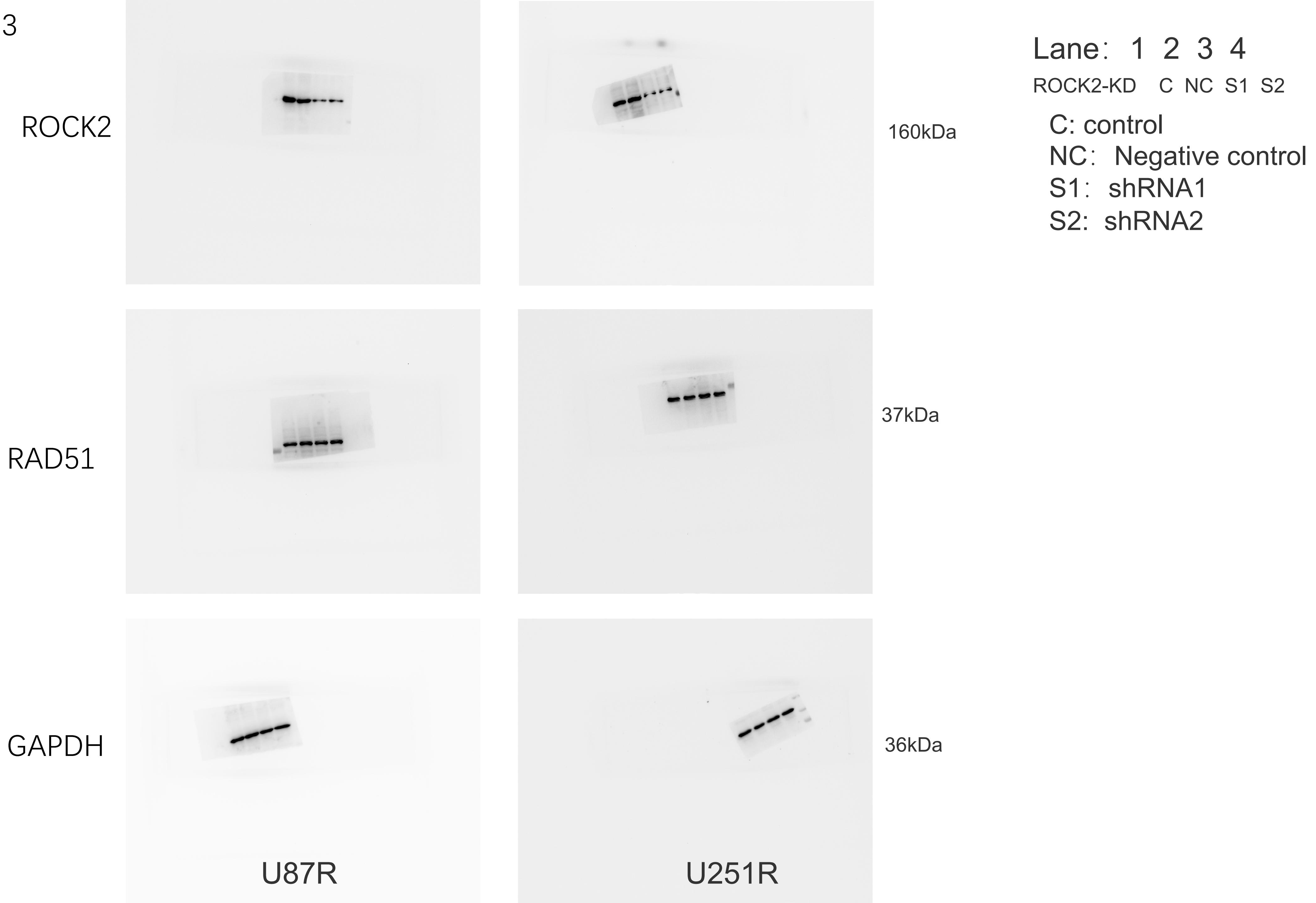
ROCK2
RAD51
GAPDH
160kDa
37kDa
36kDa
U87R
U251R
Lane： 1 2 3 4
ROCK2-KD C NC S1 S2
C: control
NC： Negative control
S1： shRNA1
S2: shRNA2

Fig.4
H

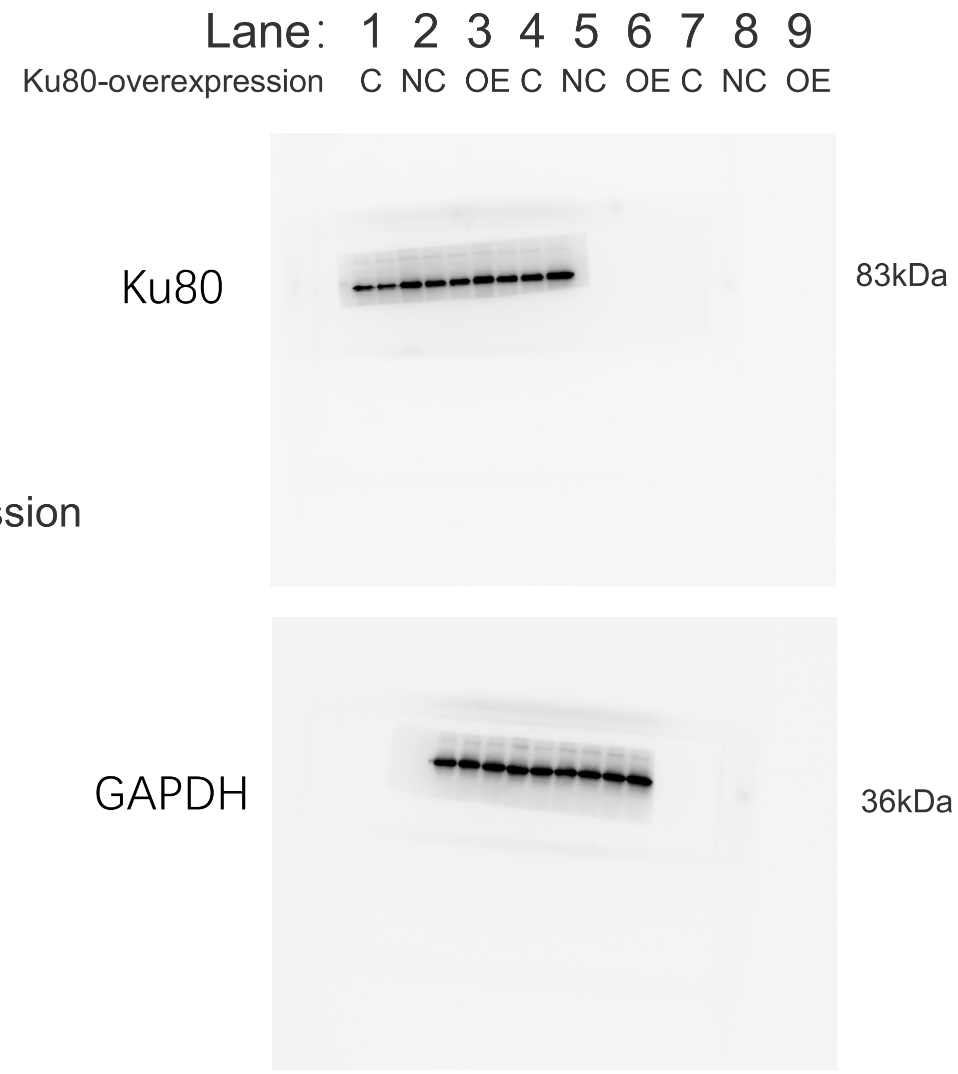

C: control
NC: Negative control
OE: Ku80-overexpression
Lane 1-3: U87R
Lane 4-6: U251R
Lane 7-9: A172R

Fig.5
C

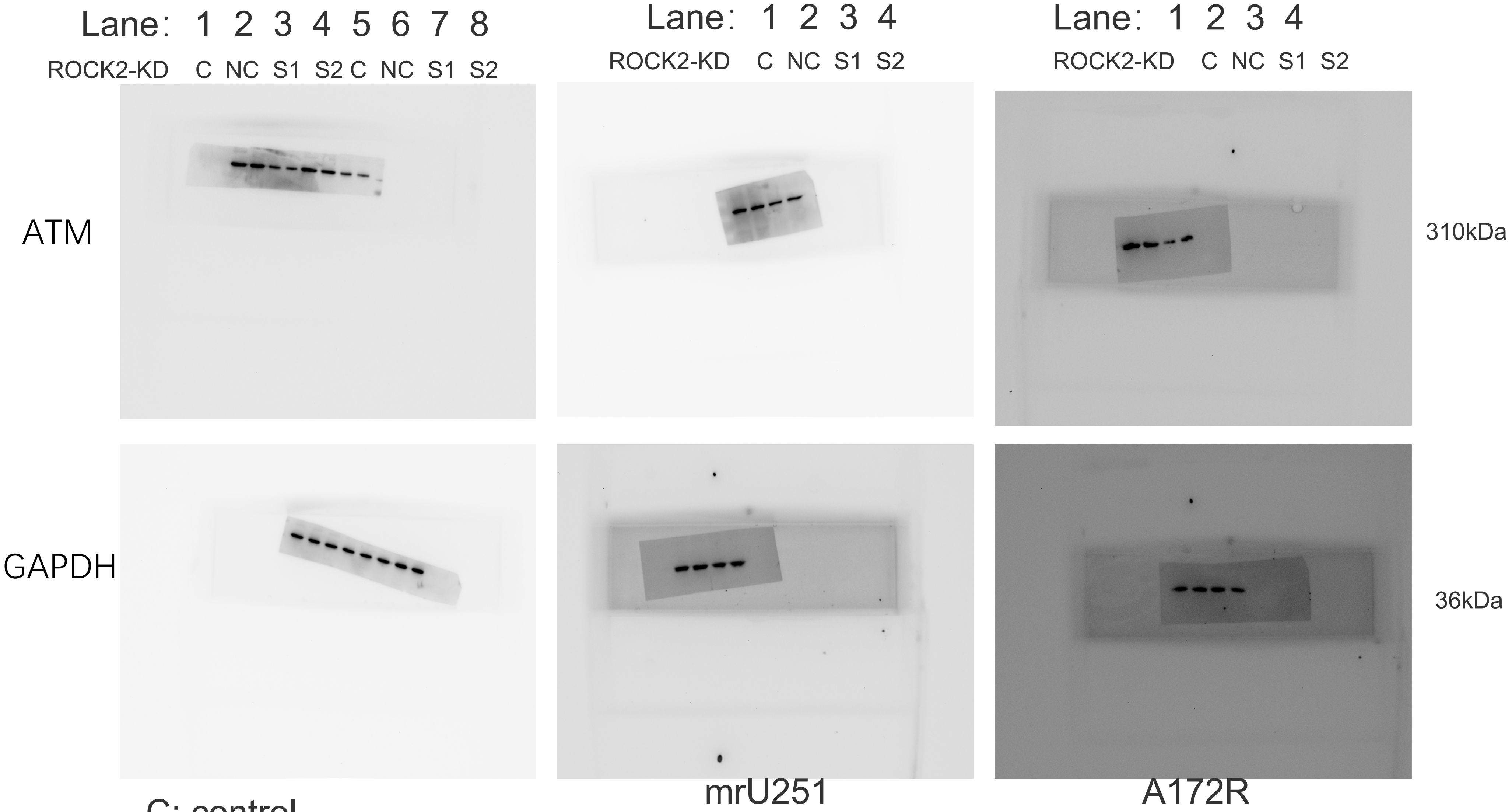

C: control
NC: Negative control
S1: shRNA1
S2: shRNA2
Lane1-4: U87R
Lane5-8: U251R

C: control
NC: Negative control
S1: shRNA1
S2: shRNA2

C: control
NC: Negative control
S1: shRNA1
S2: shRNA2

Fig.5
F

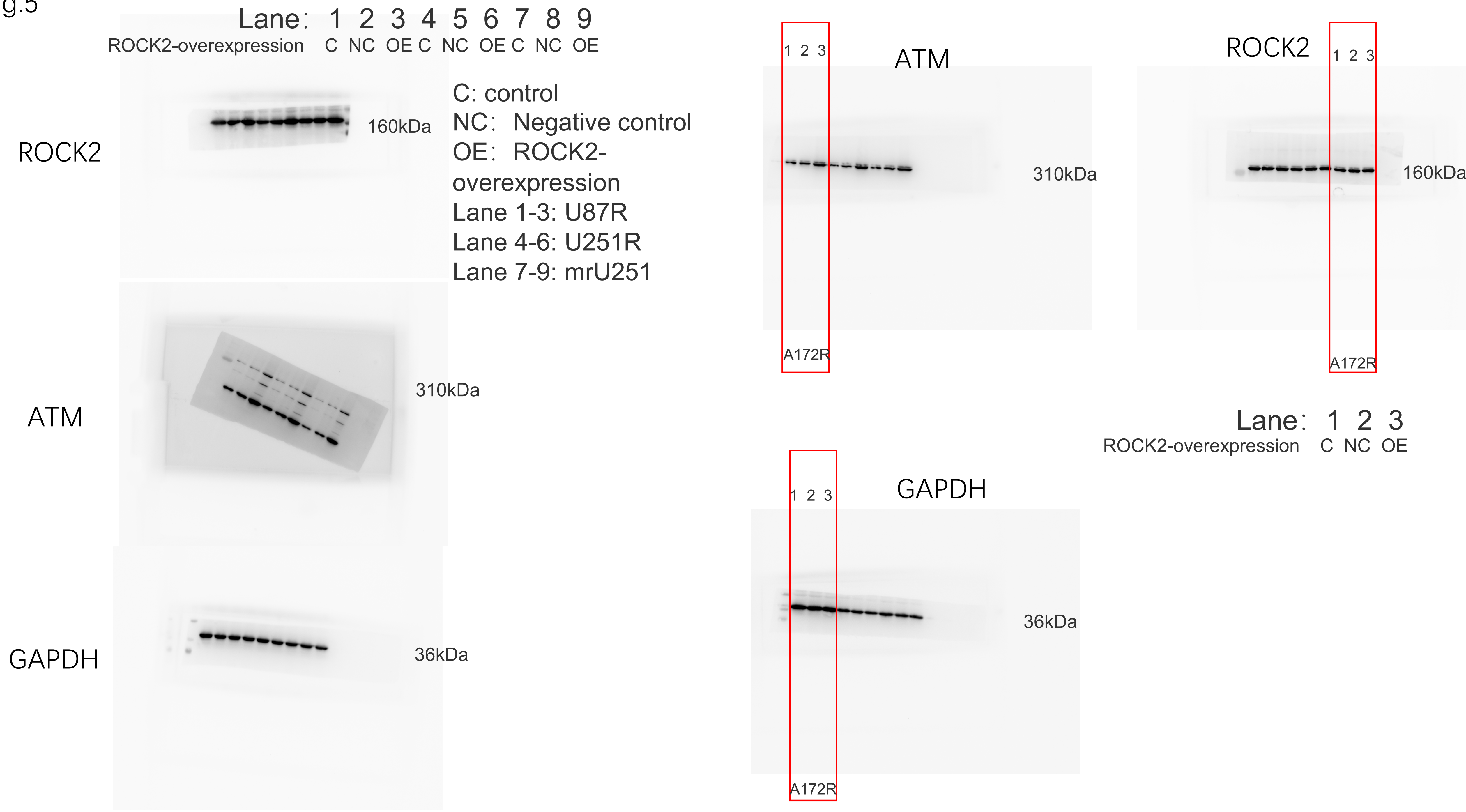

Lane: 1 2 3 4 5 6 7 8 9
ROCK2-overexpression C NC OE C NC OE C NC OE
ROCK2
160kDa
C: control
NC: Negative control
OE: ROCK2-overexpression
Lane 1-3: U87R
Lane 4-6: U251R
Lane 7-9: mrU251
ATM
310kDa
GAPDH
36kDa
1 2 3
ATM
310kDa
A172R
ROCK2
1 2 3
160kDa
A172R
Lane: 1 2 3
ROCK2-overexpression C NC OE
1 2 3
GAPDH
36kDa
A172R

Fig.5

J

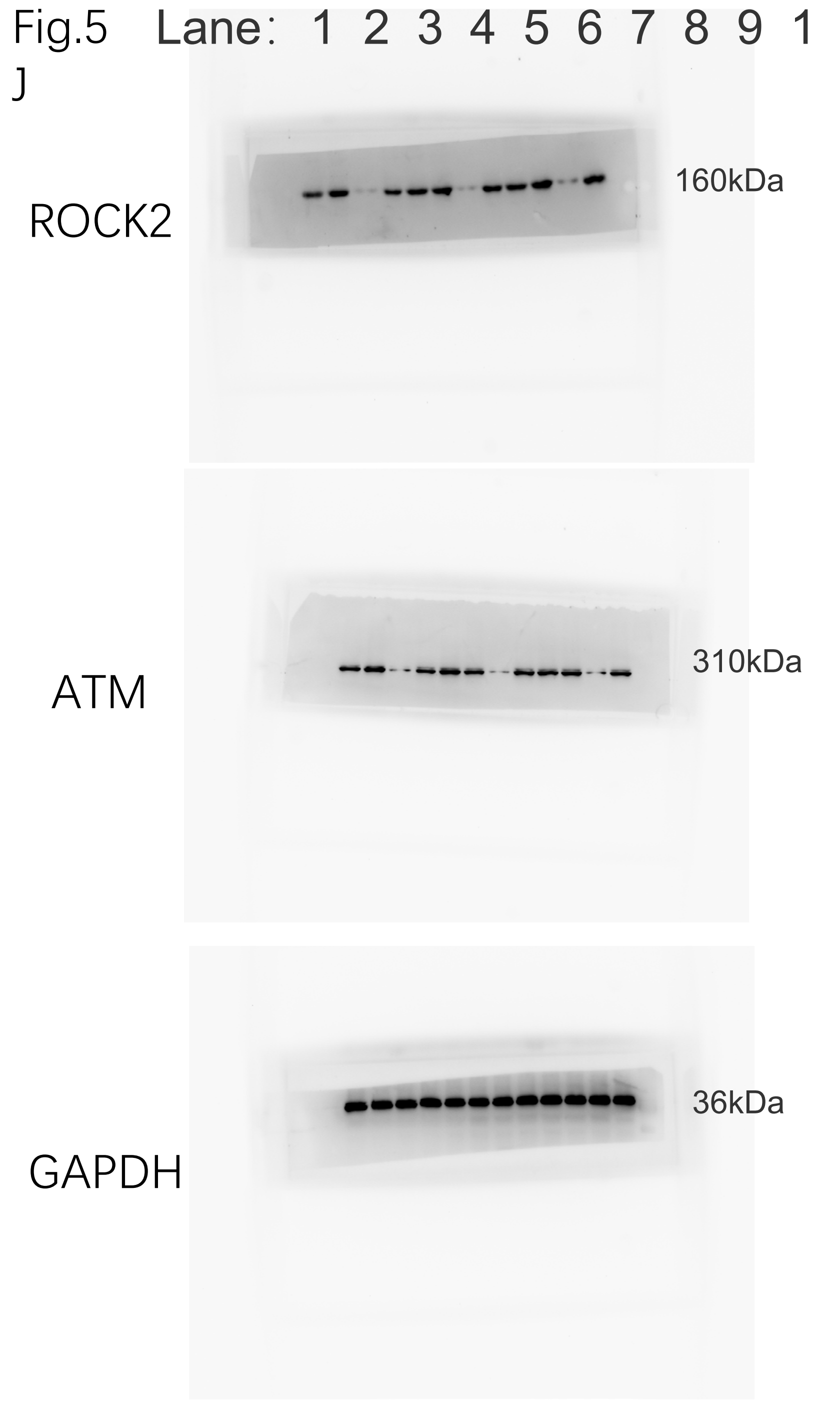

1: Control
2: Negative control
3: ROCK2-TET-kd+dox
4: ROCK2-TET-kd+dox (removal)
5: Control
6: Negative control
7: ROCK2-TET-kd+dox
8: ROCK2-TET-kd+dox (removal)
9: Control
10: Negative control
11: ROCK2-TET-kd+dox
12: ROCK2-TET-kd+dox (removal)

Lane 1-4: U87R
Lane 5-8: U251R
Lane 9-12: mrU251

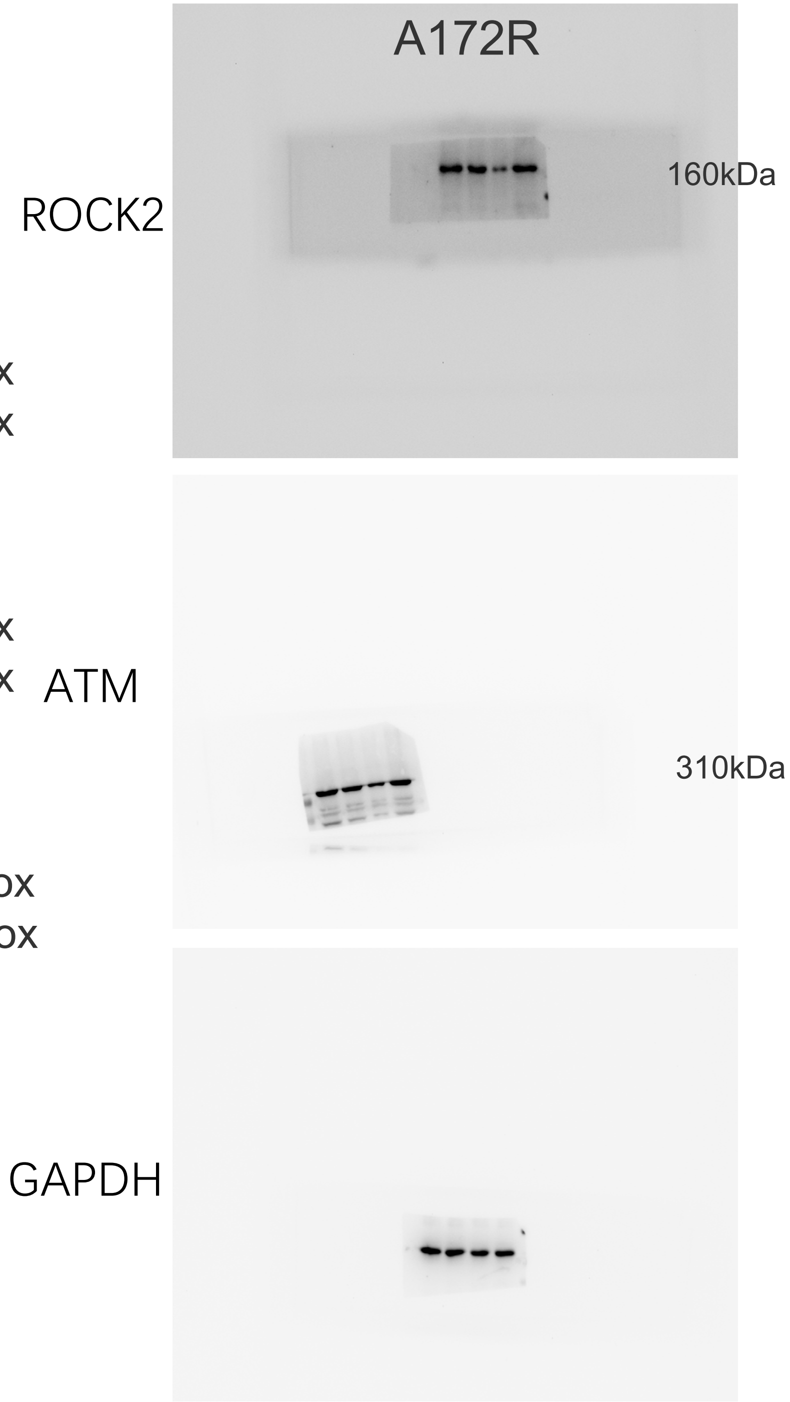

1: Control
2: Negative control
3: ROCK2-TET-kd+dox
4: ROCK2-TET-kd+dox (removal)

Fig.7
E

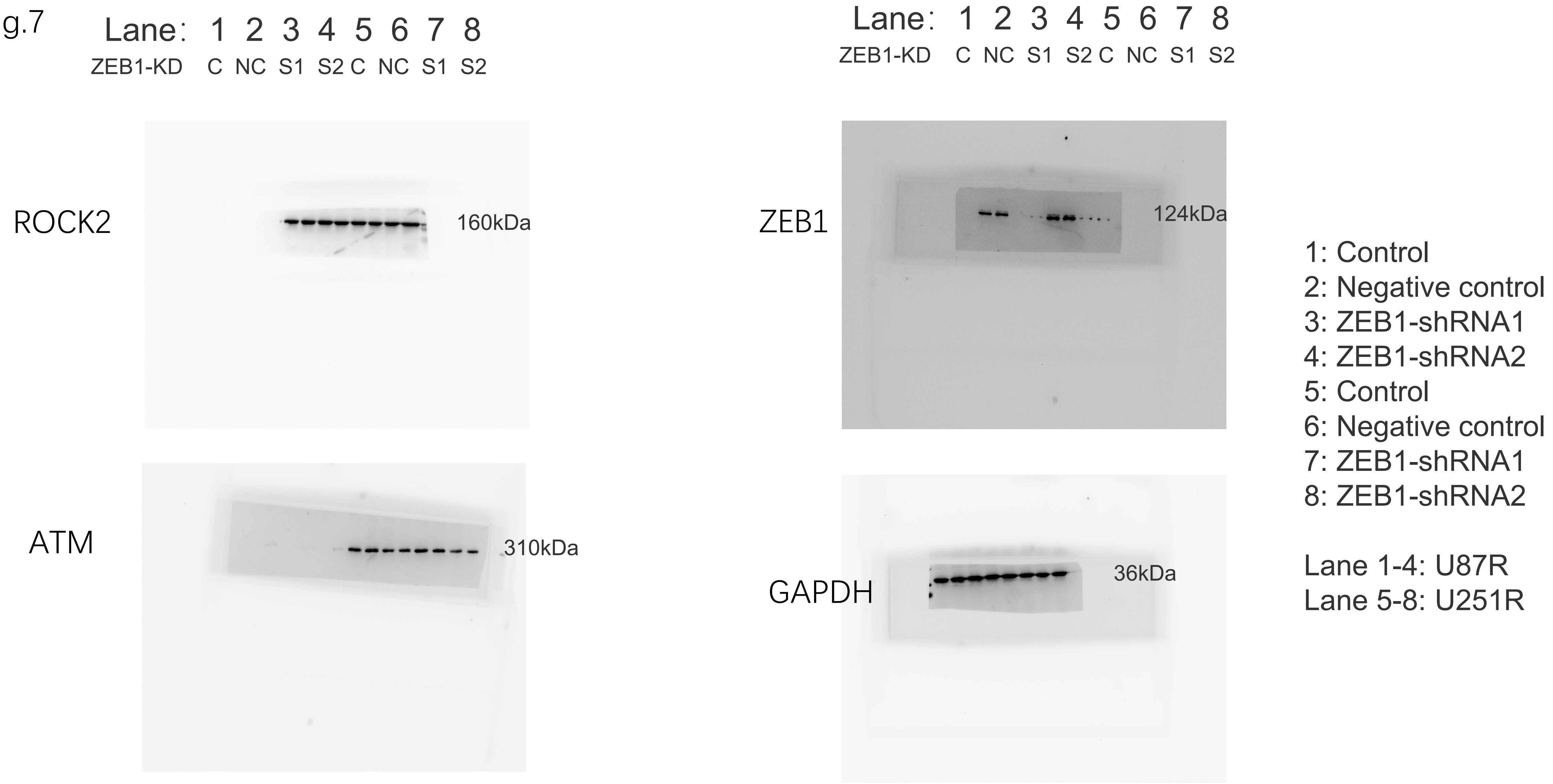
Lane: 1 2 3 4 5 6 7 8
ZEB1-KD C NC S1 S2 C NC S1 S2
ROCK2
160kDa
ATM
310kDa
Lane: 1 2 3 4 5 6 7 8
ZEB1-KD C NC S1 S2 C NC S1 S2
ZEB1
124kDa
GAPDH
36kDa
1: Control
2: Negative control
3: ZEB1-shRNA1
4: ZEB1-shRNA2
5: Control
6: Negative control
7: ZEB1-shRNA1
8: ZEB1-shRNA2
Lane 1-4: U87R
Lane 5-8: U251R

Fig.7
I

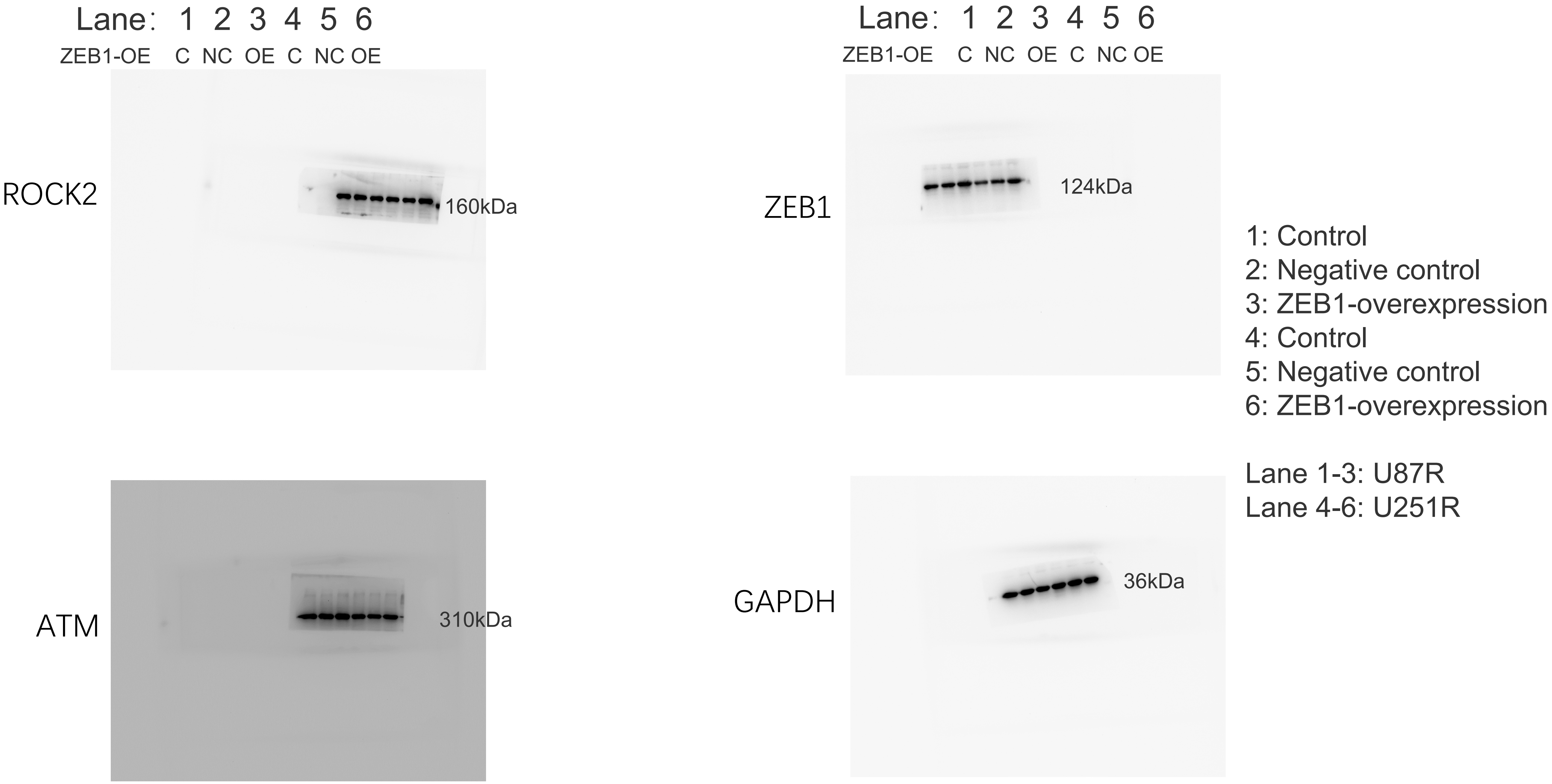
Lane: 1 2 3 4 5 6
ZEB1-OE C NC OE C NC OE
ROCK2
160kDa
ATM
310kDa
Lane: 1 2 3 4 5 6
ZEB1-OE C NC OE C NC OE
ZEB1
124kDa
GAPDH
36kDa
1: Control
2: Negative control
3: ZEB1-overexpression
4: Control
5: Negative control
6: ZEB1-overexpression
Lane 1-3: U87R
Lane 4-6: U251R

Fig.7
M

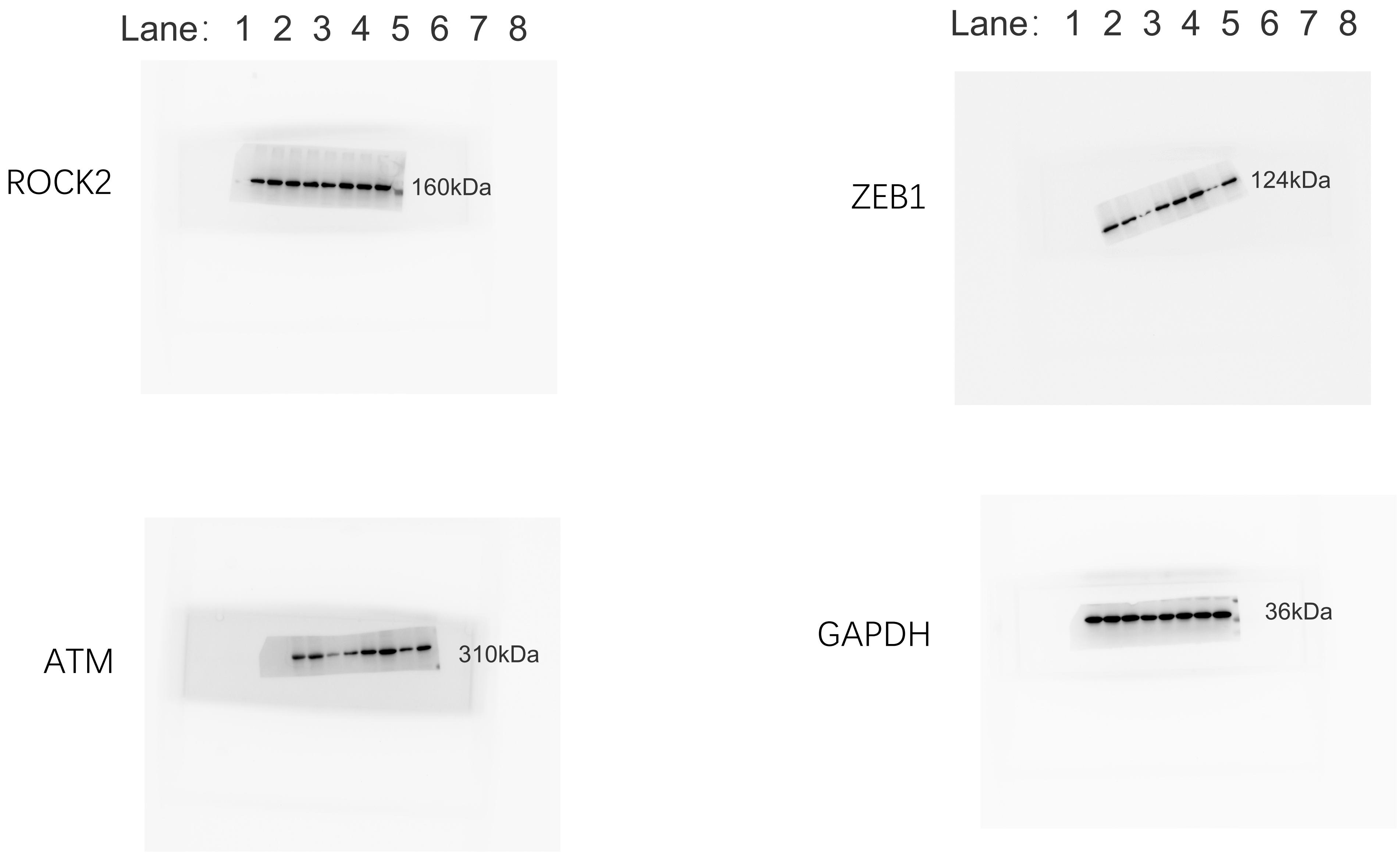

1: Control
2: Negative control
3: ZEB1-TET-kd+dox
4: ZEB1-TET-kd+dox (removal)
5: Control
6: Negative control
7: ZEB1-TET-kd+dox
8: ZEB1-TET-kd+dox (removal)

Lane 1-4: U87R
Lane 5-8: U251R

Fig.8
A

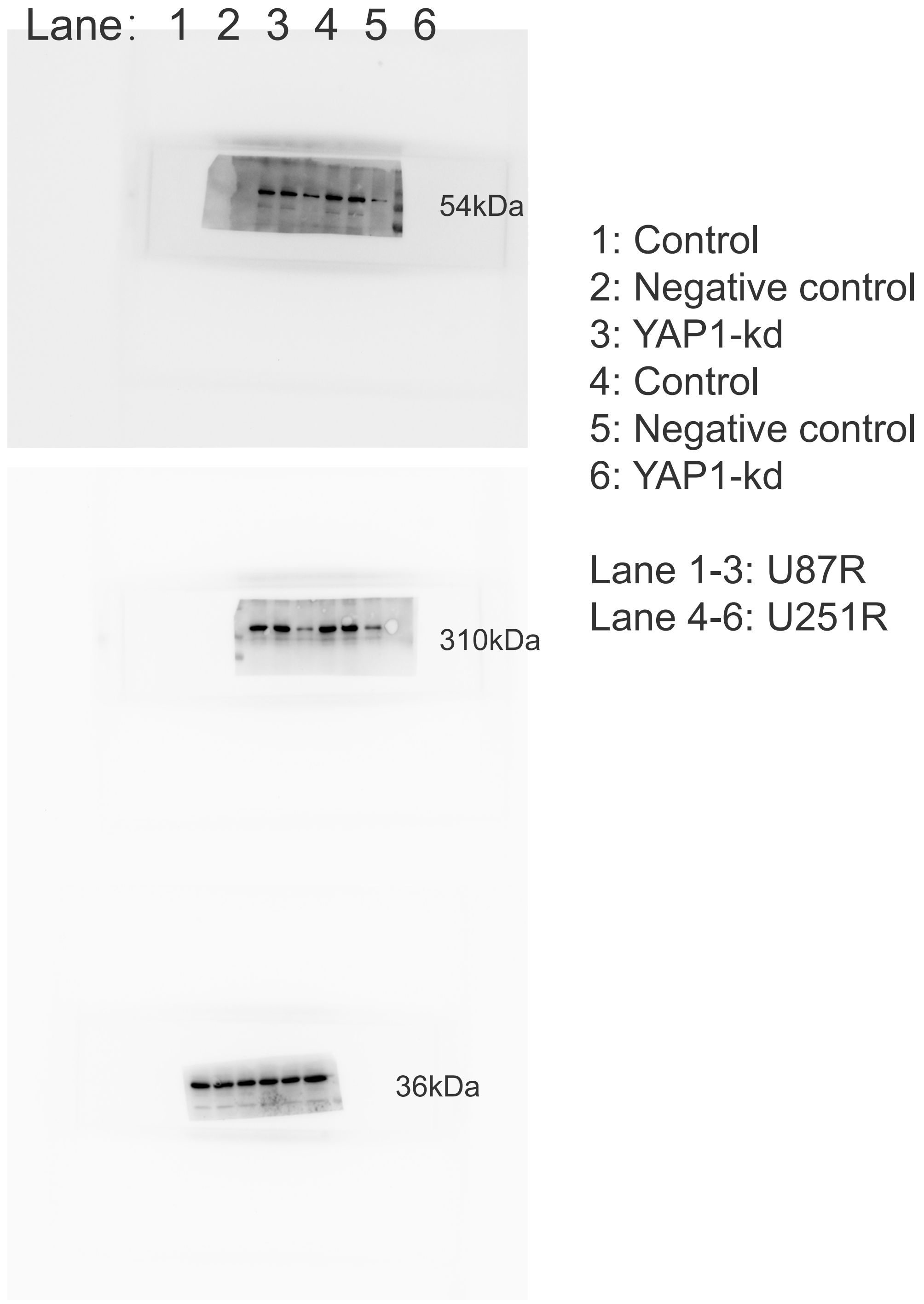
Lane: 1 2 3 4 5 6
YAP1
54kDa
ATM
310kDa
GAPDH
36kDa
1: Control
2: Negative control
3: YAP1-kd
4: Control
5: Negative control
6: YAP1-kd
Lane 1-3: U87R
Lane 4-6: U251R

Fig.8
B

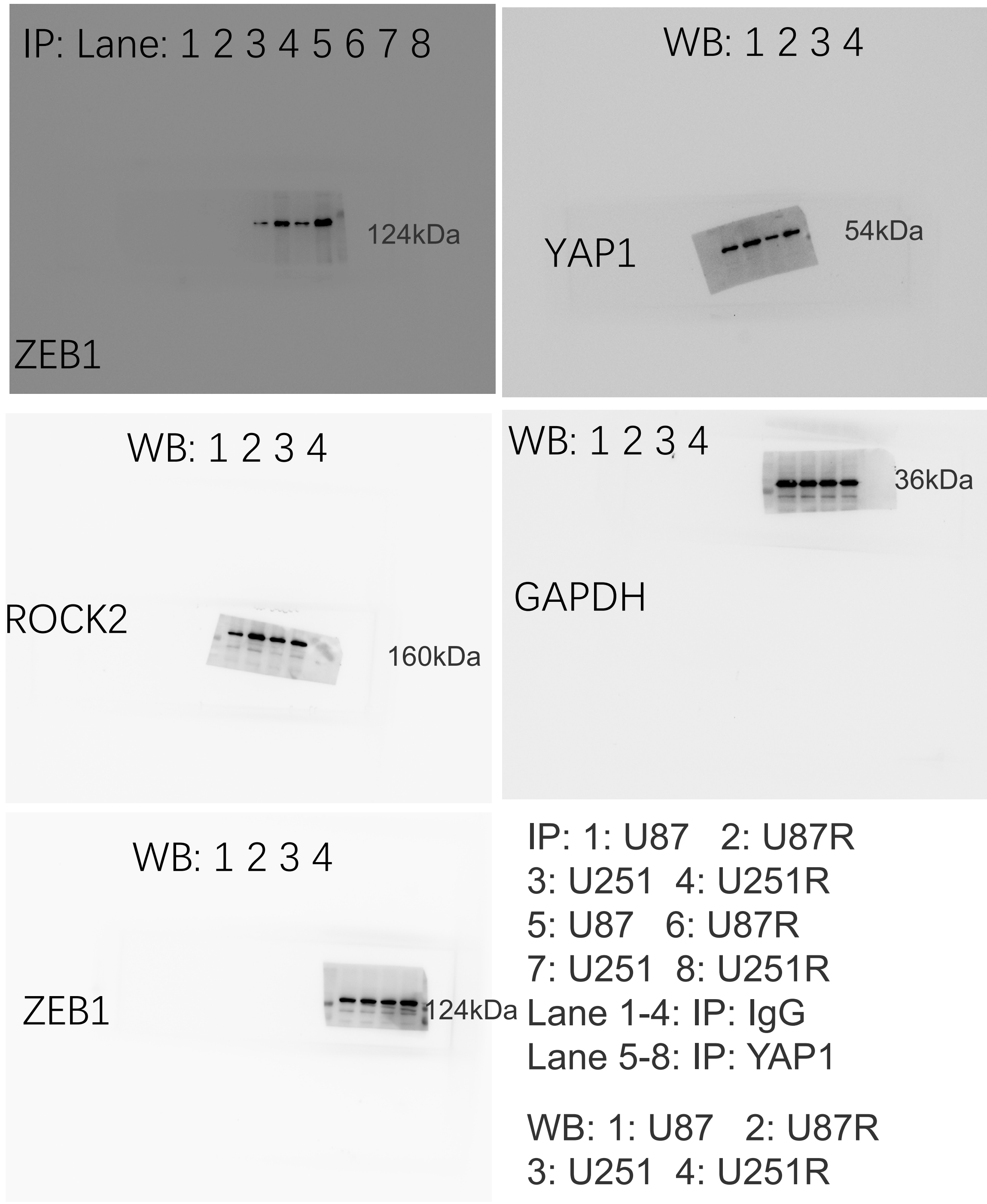
IP: Lane: 1 2 3 4 5 6 7 8
124kDa
ZEB1
WB: 1 2 3 4
YAP1
54kDa
WB: 1 2 3 4
ROCK2
160kDa
WB: 1 2 3 4
36kDa
GAPDH
WB: 1 2 3 4
ZEB1
124kDa
IP: 1: U87 2: U87R
3: U251 4: U251R
5: U87 6: U87R
7: U251 8: U251R
Lane 1-4: IP: IgG
Lane 5-8: IP: YAP1
WB: 1: U87 2: U87R
3: U251 4: U251R

Fig.8
C

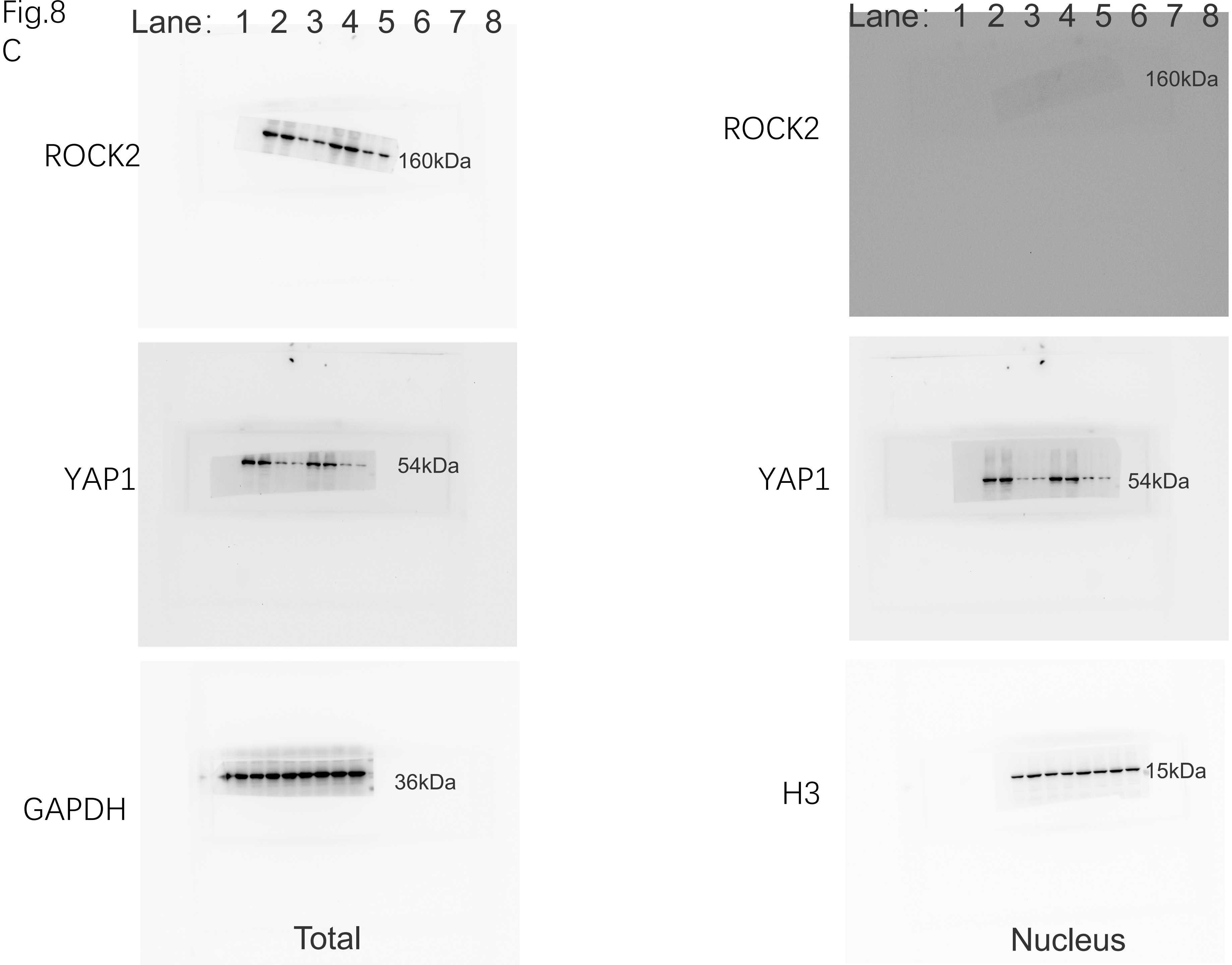

1: Control
2: Negative control
3: ROCK2-shRNA1
4: ROCK2-shRNA2
5: Control
6: Negative control
7: ROCK2-shRNA1
8: ROCK2-shRNA2

Lane 1-4: U87R
Lane 5-8: U251R

Fig.8
H

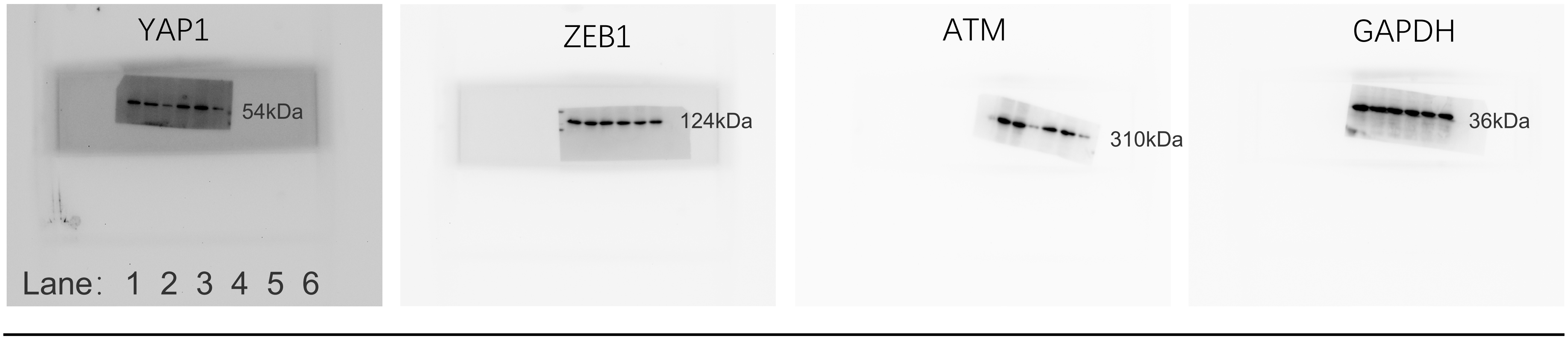

Total

1: Control 2: Negative control 3: YAP-shRNA 4: Control 5: Negative control 6: YAP-shRNA
Lane 1-3: U87R
Lane 4-6: U251R

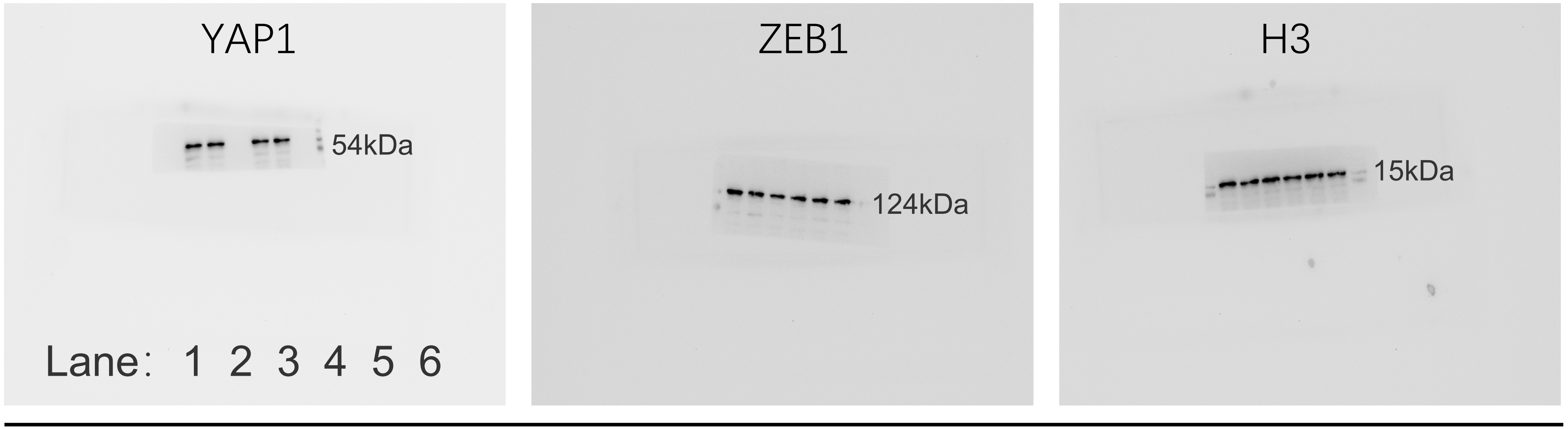

Nucleus

Fig.8
K

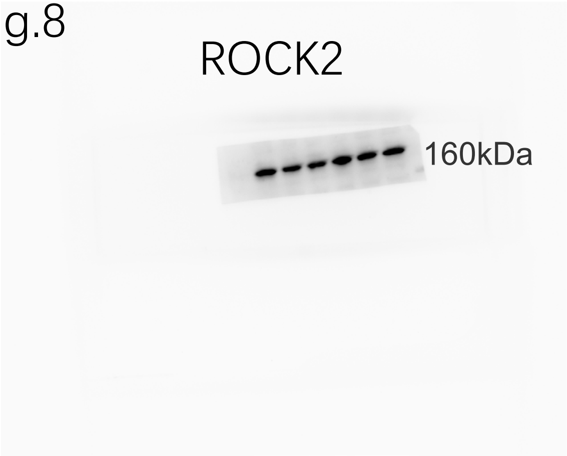

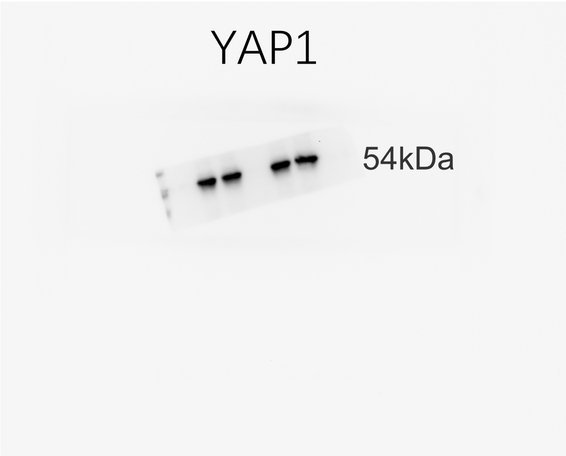

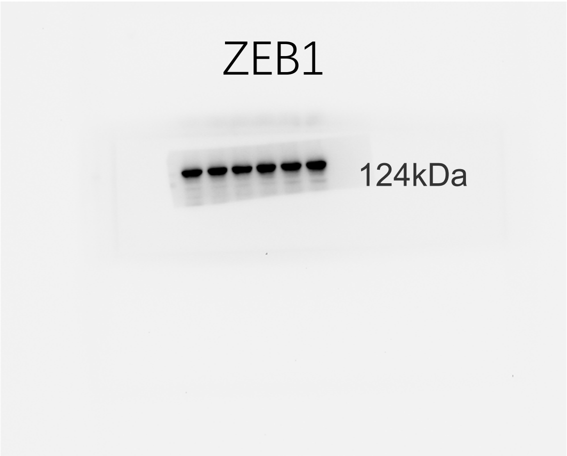

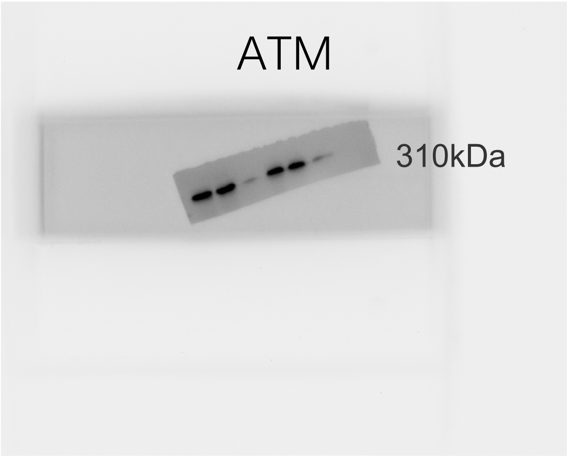

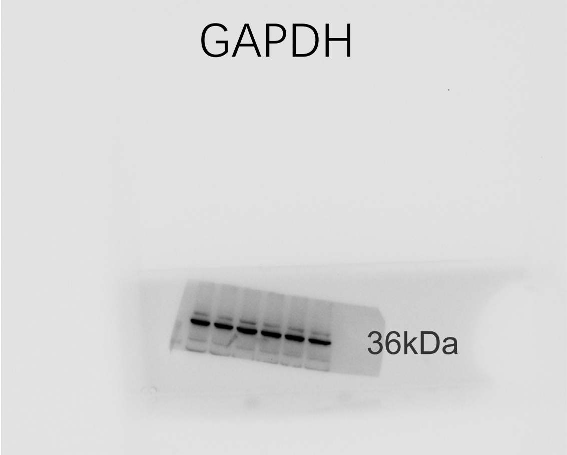

WB: Lane: 1 2 3 4 5 6

1: Control 2: Negative control 3: YAP-shRNA 4: Control 5: Negative control 6: YAP-shRN[illegible]
Lane 1-3: U87R
Lane 4-6: U87R-ROCK2-OE clones

IP: Lane: 1 2 3 4 5 6

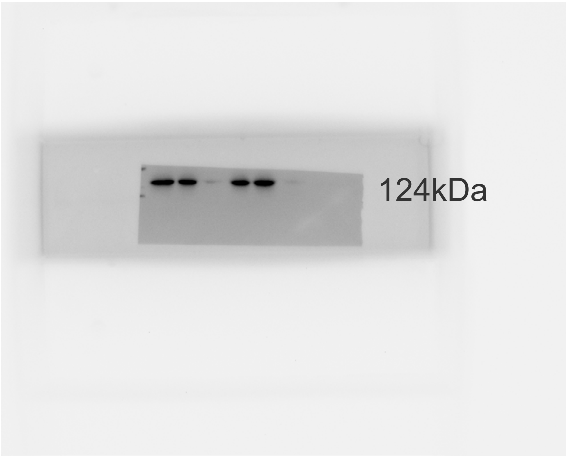

IP: YAP1, IB: ZEB1

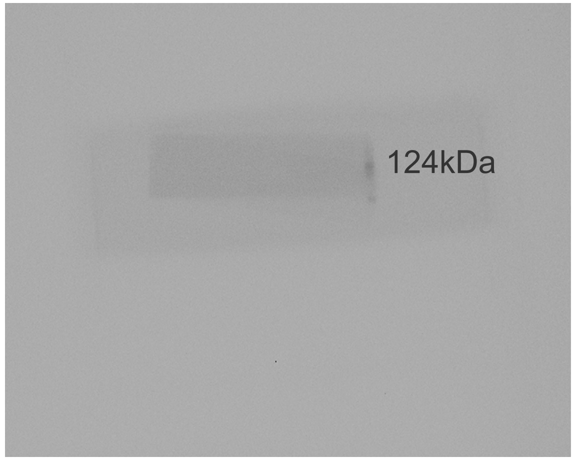

IP: IgG, IB: ZEB1

1: Control 2: Negative control 3: YAP-shRNA 4: Control 5: Negative control 6: YAP-shRNA
Lane 1-3: U87R
Lane 4-6: U87R-ROCK2-OE clones

Fig.8
K

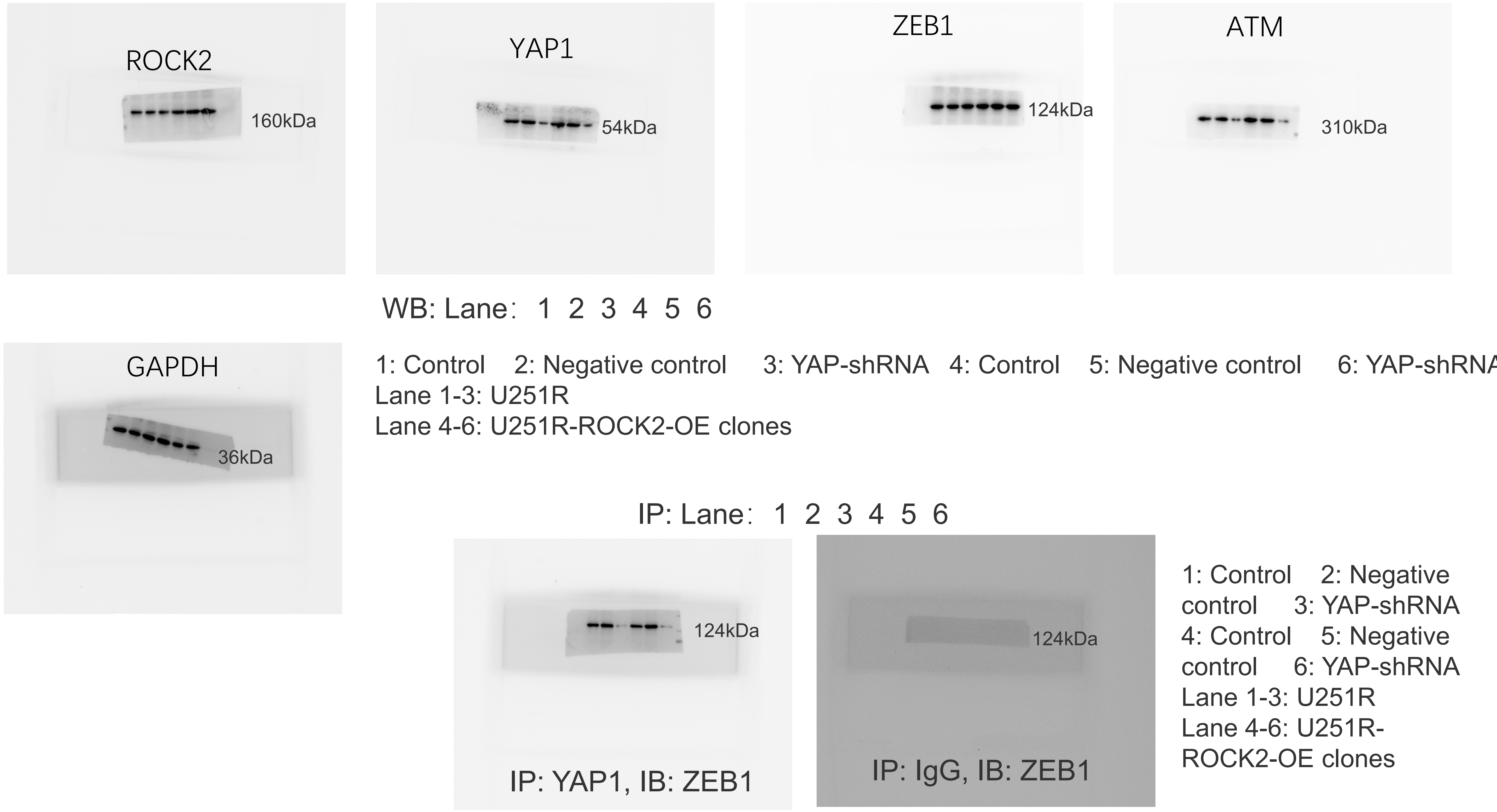
ROCK2
160kDa
YAP1
54kDa
ZEB1
124kDa
ATM
310kDa
WB: Lane: 1 2 3 4 5 6
GAPDH
36kDa
1: Control 2: Negative control 3: YAP-shRNA 4: Control 5: Negative control 6: YAP-shRNA
Lane 1-3: U251R
Lane 4-6: U251R-ROCK2-OE clones
IP: Lane: 1 2 3 4 5 6
124kDa
IP: YAP1, IB: ZEB1
124kDa
IP: IgG, IB: ZEB1
1: Control 2: Negative control 3: YAP-shRNA
4: Control 5: Negative control 6: YAP-shRNA
Lane 1-3: U251R
Lane 4-6: U251R-ROCK2-OE clones

Fig.8
L

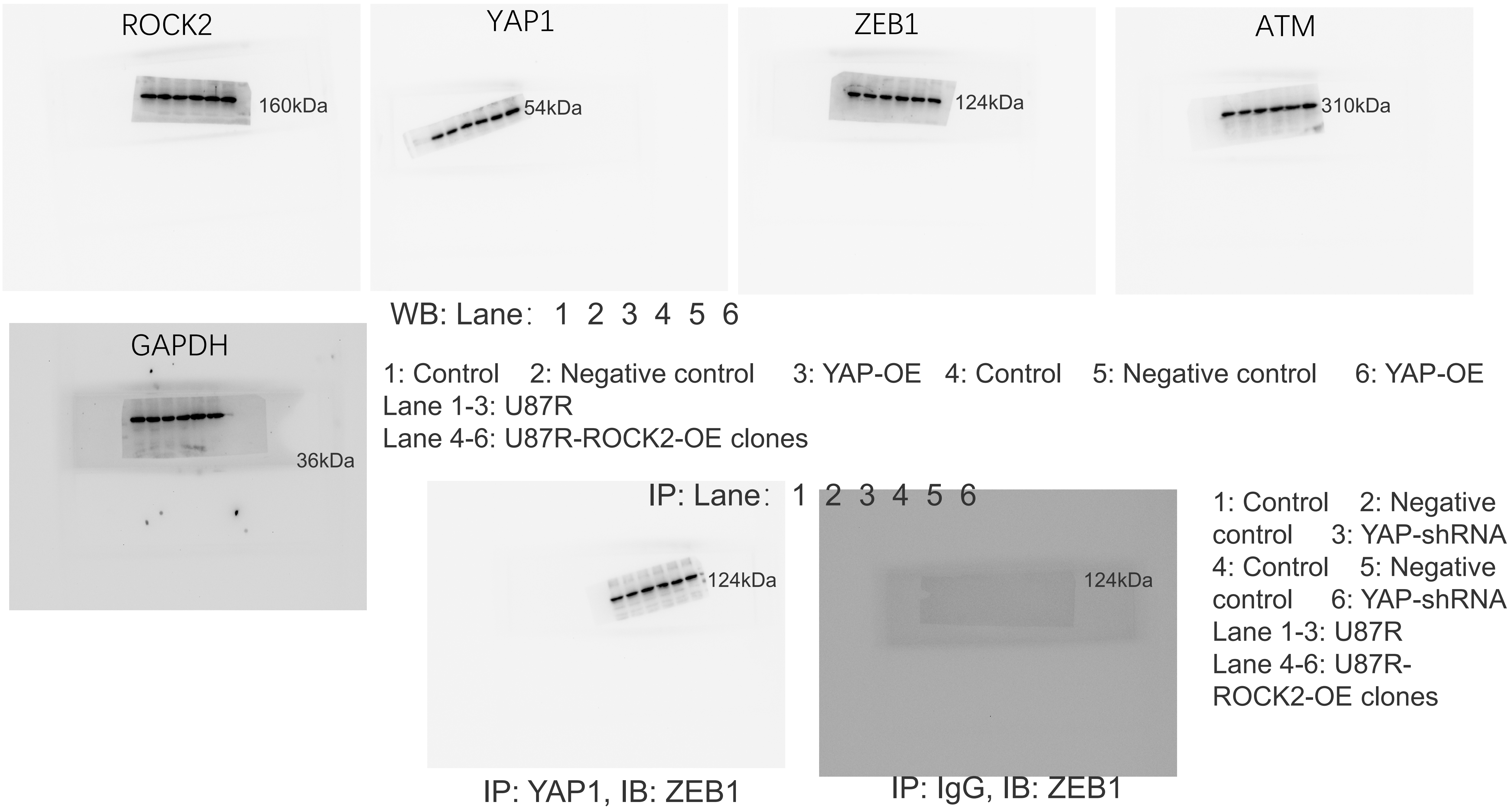
ROCK2
160kDa
YAP1
54kDa
ZEB1
124kDa
ATM
310kDa
GAPDH
36kDa
WB: Lane: 1 2 3 4 5 6
1: Control 2: Negative control 3: YAP-OE 4: Control 5: Negative control 6: YAP-OE
Lane 1-3: U87R
Lane 4-6: U87R-ROCK2-OE clones
IP: Lane: 1 2 3 4 5 6
124kDa
124kDa
1: Control 2: Negative control 3: YAP-shRNA
4: Control 5: Negative control 6: YAP-shRNA
Lane 1-3: U87R
Lane 4-6: U87R-ROCK2-OE clones
IP: YAP1, IB: ZEB1
IP: IgG, IB: ZEB1

Fig.8
L

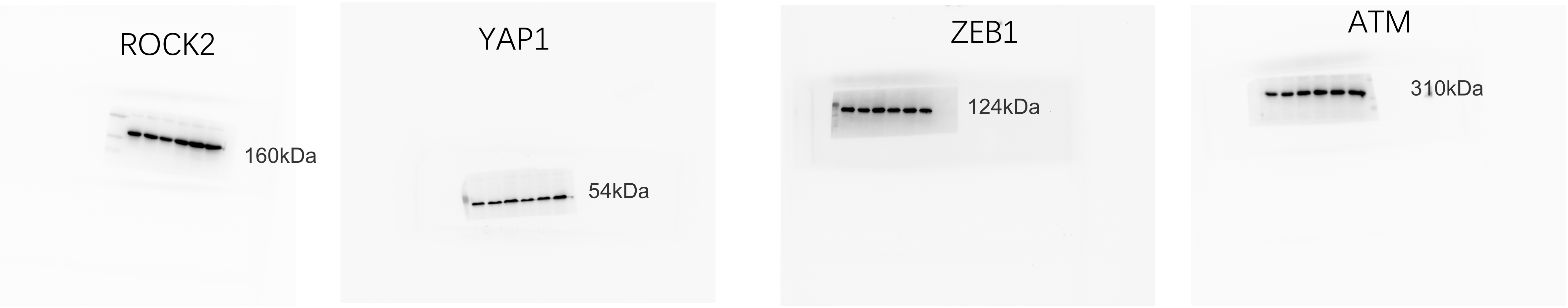

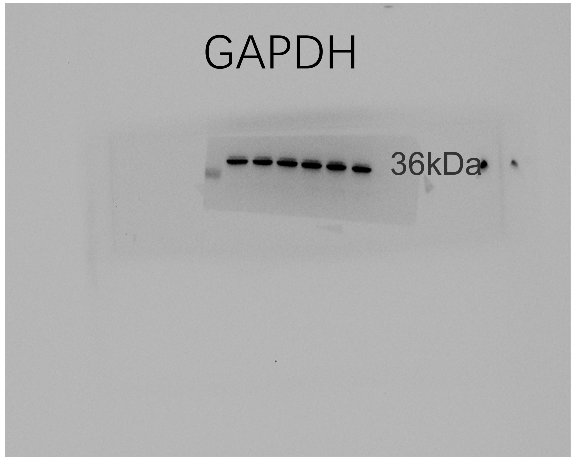

WB: Lane：1 2 3 4 5 6

1: Control 2: Negative control 3: YAP-shRNA 4: Control 5: Negative control 6: YAP-shRNA
Lane 1-3: U251R
Lane 4-6: U251R-ROCK2-OE clones

IP: Lane：1 2 3 4 5 6

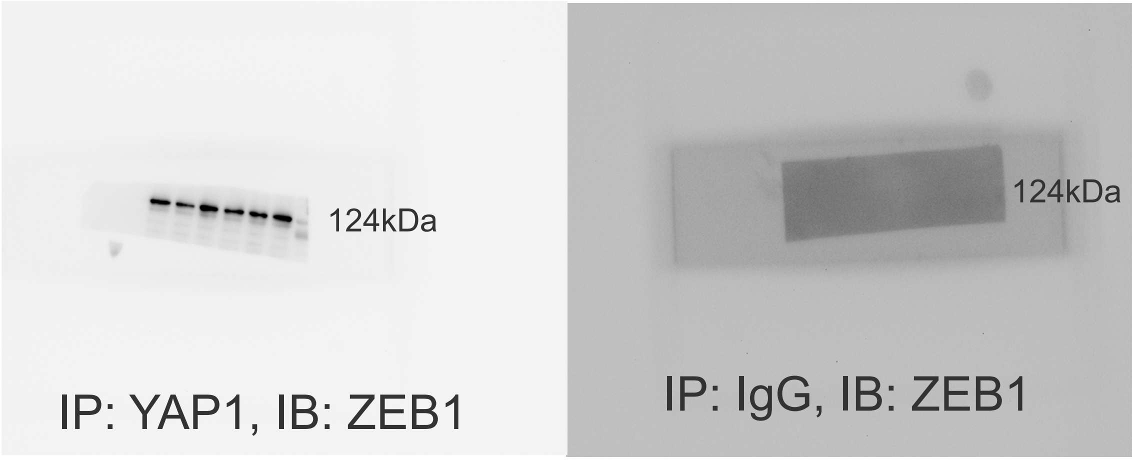

1: Control 2: Negative control 3: YAP-shRNA
4: Control 5: Negative control 6: YAP-shRNA
Lane 1-3: U251R
Lane 4-6: U251R-ROCK2-OE clones

Supplementary Figure S1

R

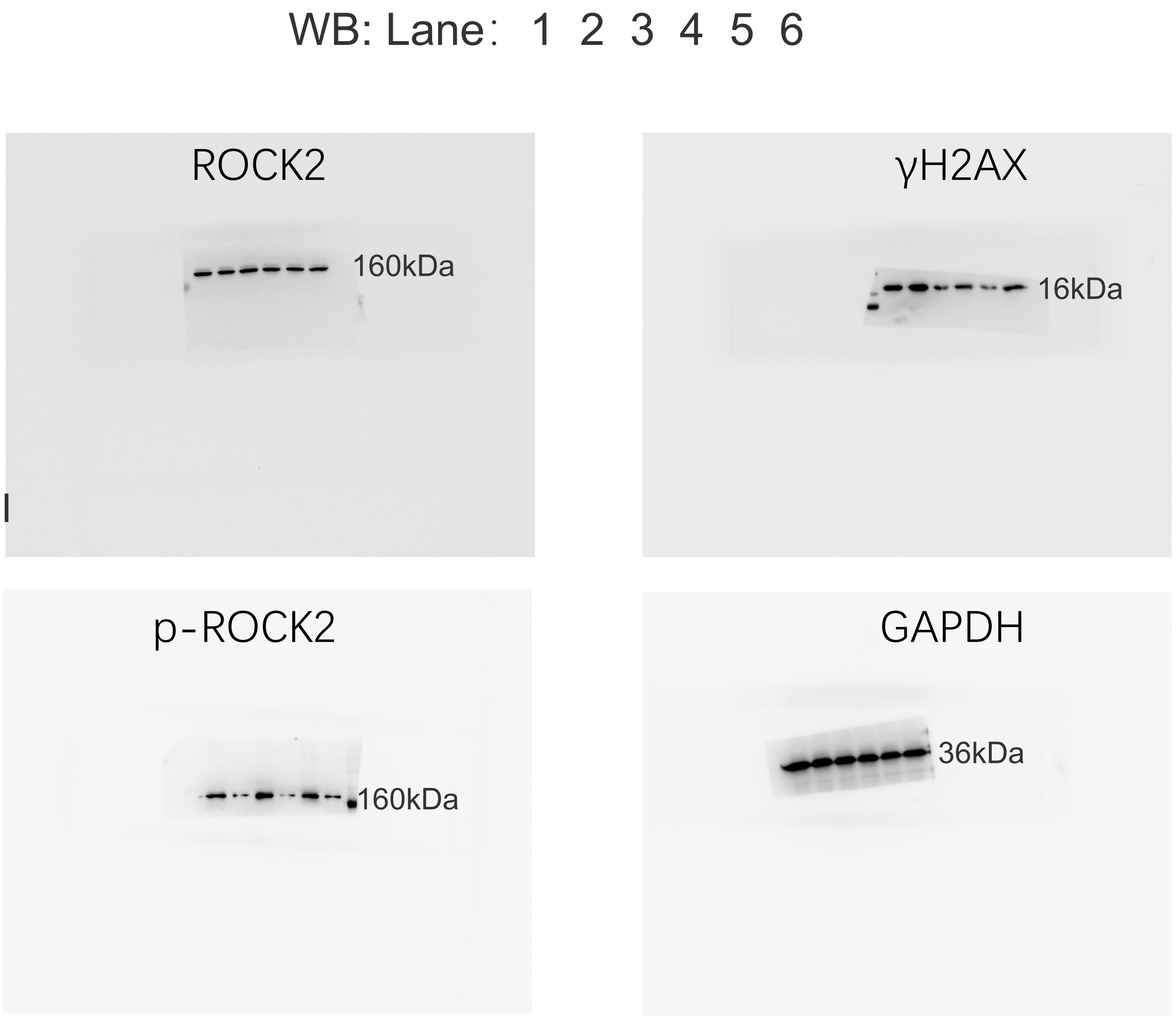

1: - 2: +Fasudil 3: - 4: +Fasudil

5: - 6: +Fasudil

Lane 1-2: A172R

Lane 3-4: U87R

Lane 5-6: U251R

Supplementary Figure S5
C

Lane: 1 2 3 4 5 6 7 8

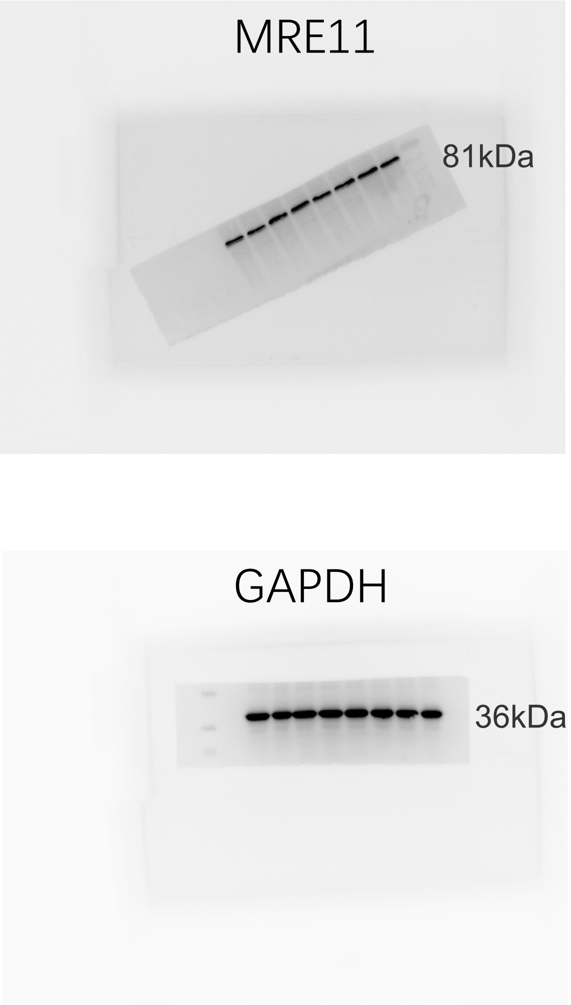

1: Control
2: Negative control
3: ROCK2-shRNA1
4: ROCK2-shRNA2
5: Control
6: Negative control
7: ROCK2-shRNA1
8: ROCK2-shRNA2
Lane 1-4: U87R
Lane 5-8: U251R

Lane: 1 2 3 4 5 6 7 8

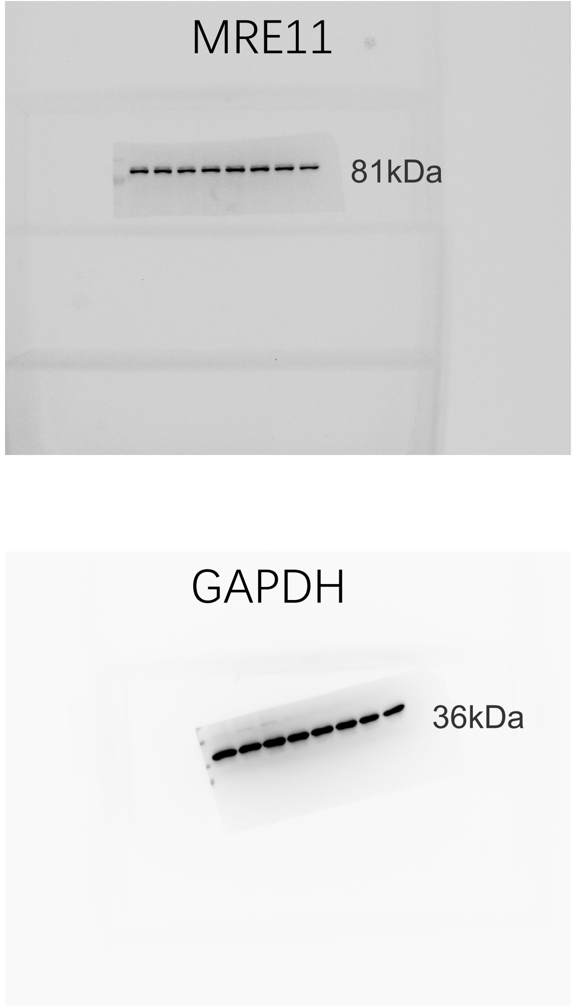

1: Control
2: Negative control
3: ROCK2-shRNA1
4: ROCK2-shRNA2
5: Control
6: Negative control
7: ROCK2-shRNA1
8: ROCK2-shRNA2
Lane 1-4: mrU251R
Lane 5-8: A172R

Supplementary Figure S7

D

Lane: 1 2 3 4 5 6 7 8 9 10 11 12

ROCK2 pROCK2 ATM GAPDH

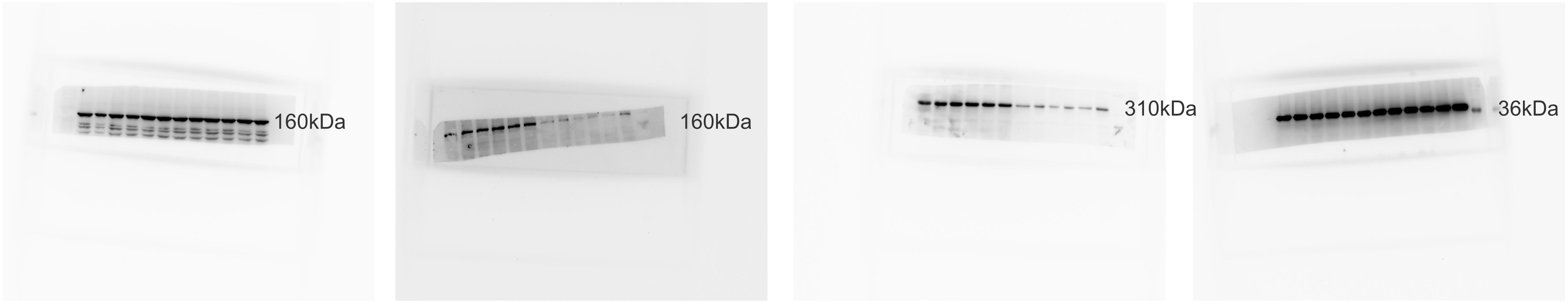

Lane 1-3 : Control
Lane 4-6 : +TMZ
Lane 7-9 : +Fasudil
Lane 1-3 : +TMZ plus Fasudil

Supplementary Figure S8

F

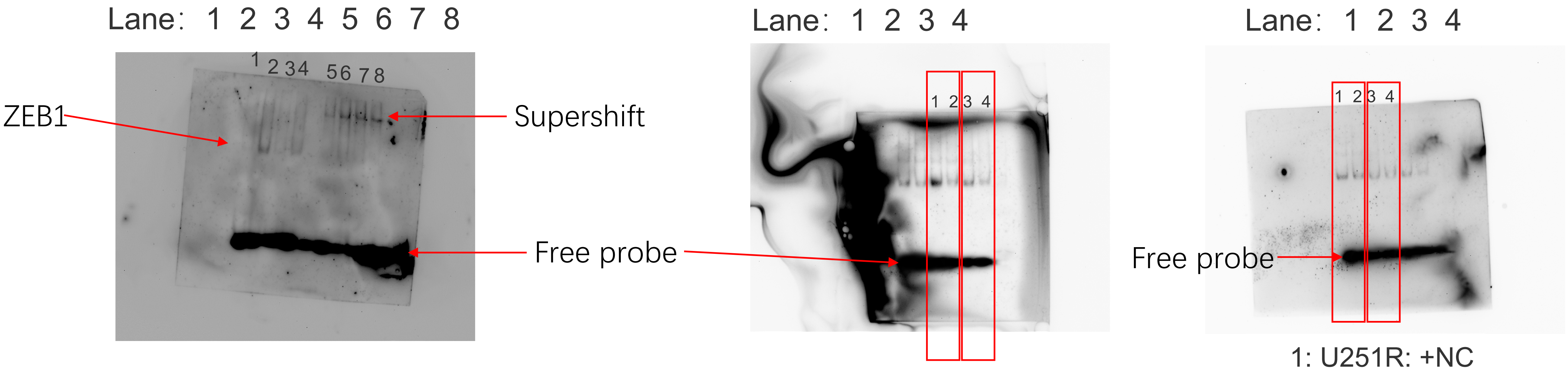

1: U87
2: U87R
3: U251
4: U251R
5: U87
6: U87R
7: U251
8: U251R
Lane 1-4: +Probe
Lane 5-8: +Probe plus anti-ZEB1

1: U87R: +NC
2: U87R: +SC
3: U87: +NC
4: U87: +SC

1: U251R: +NC
2: U251R: +SC
3: U251: +NC
4: U251: +SC

Supplementary Figure S8

H

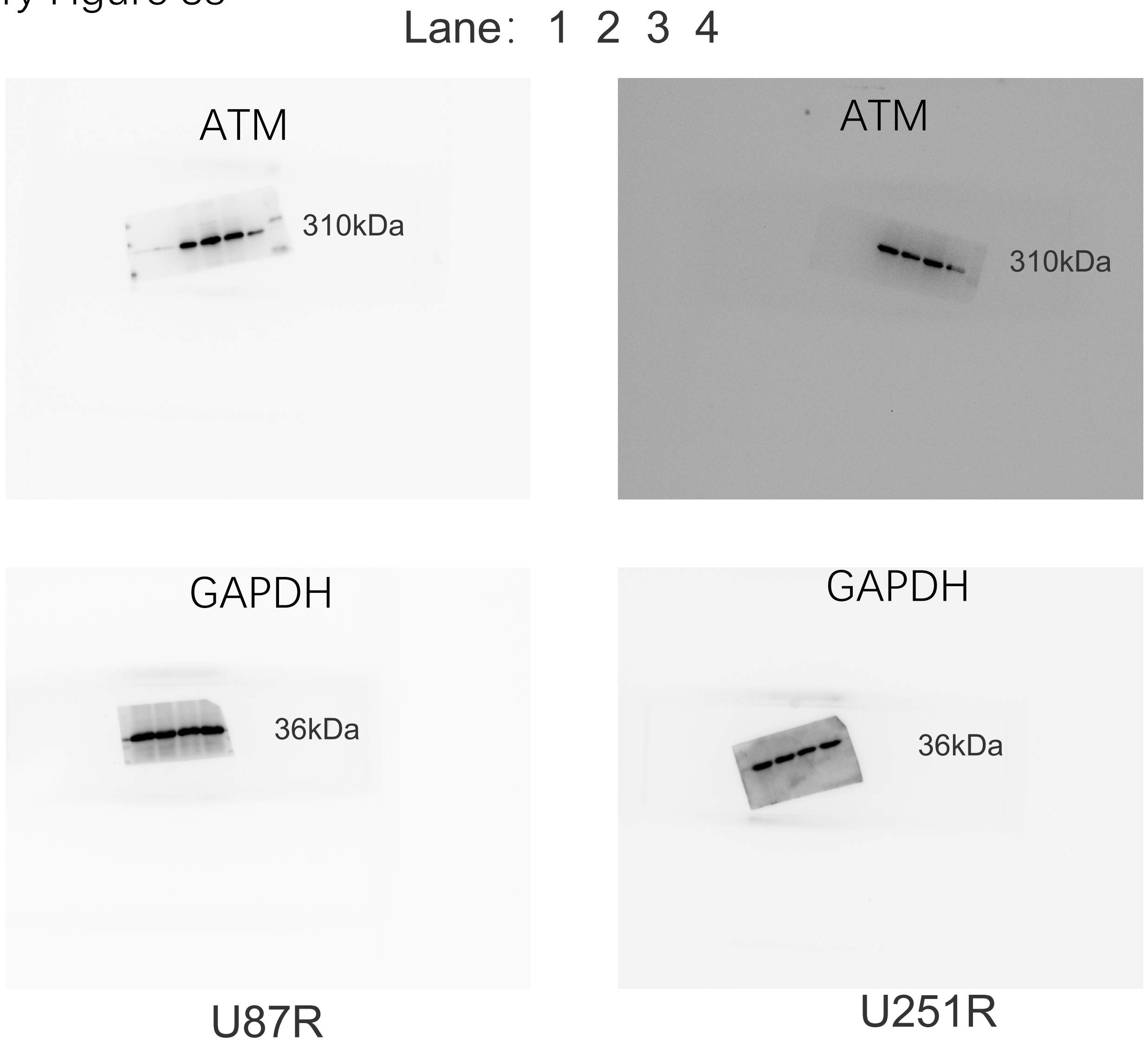

1: Control
2: pgl6
3: 1.5k-WT
4: 1.5k-646-635 mu

Supplementary Figure S8

J

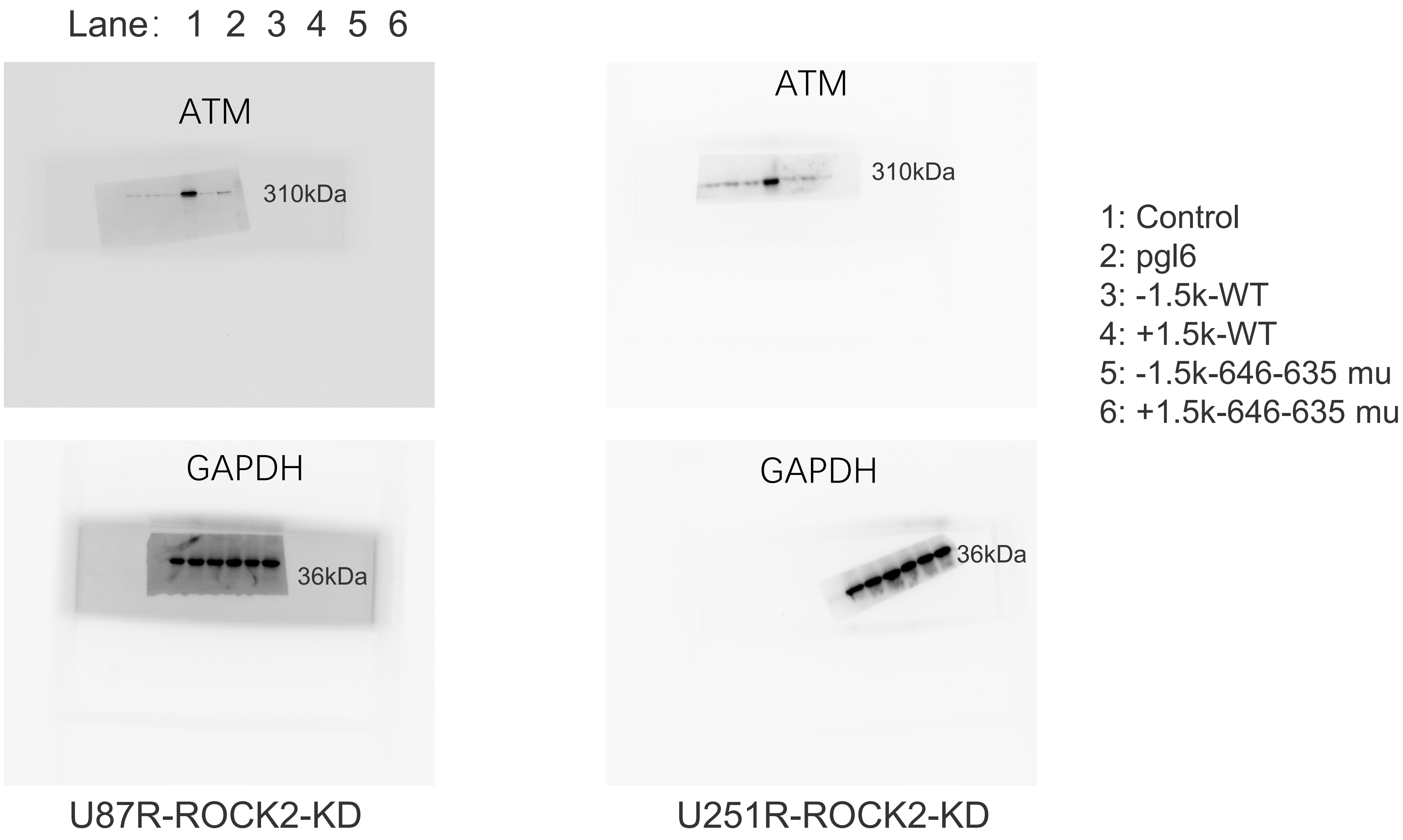

Supplementary Figure S8
L

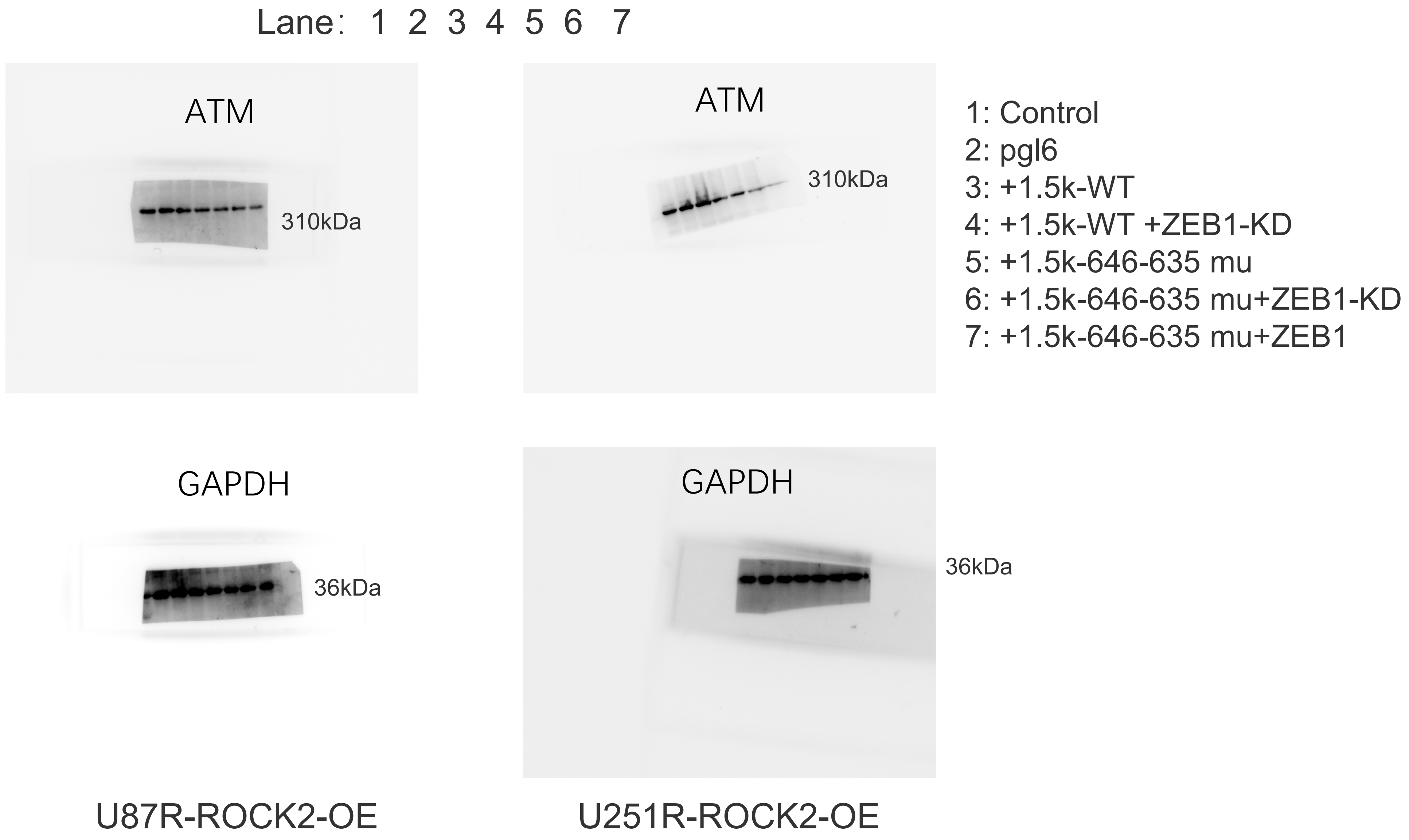

Supplementary Figure S9

A

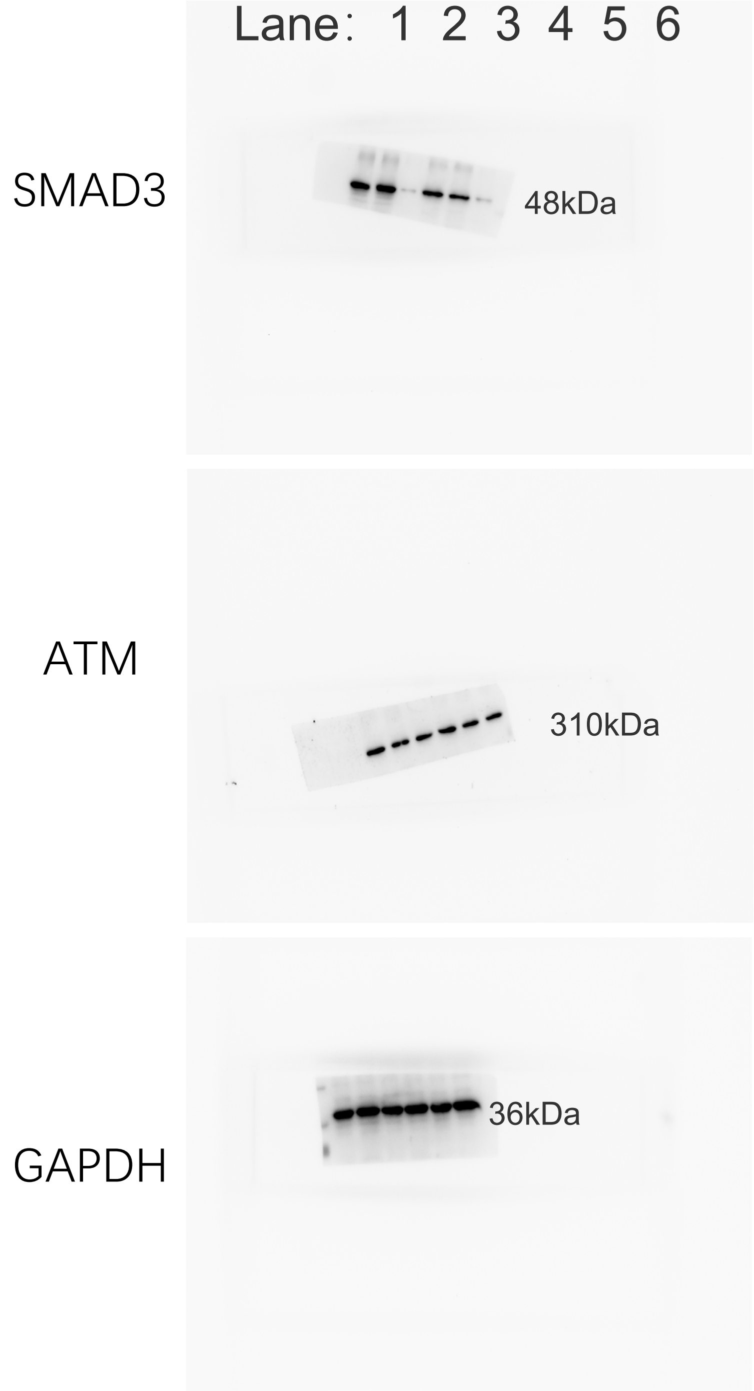

1: Control
2: Negative control
3: Smad3-KD
4: Control
5: Negative control
6: Smad3-KD
Line 1-3: U87R
Line 4-6: U251R

Supplementary Figure S9

B

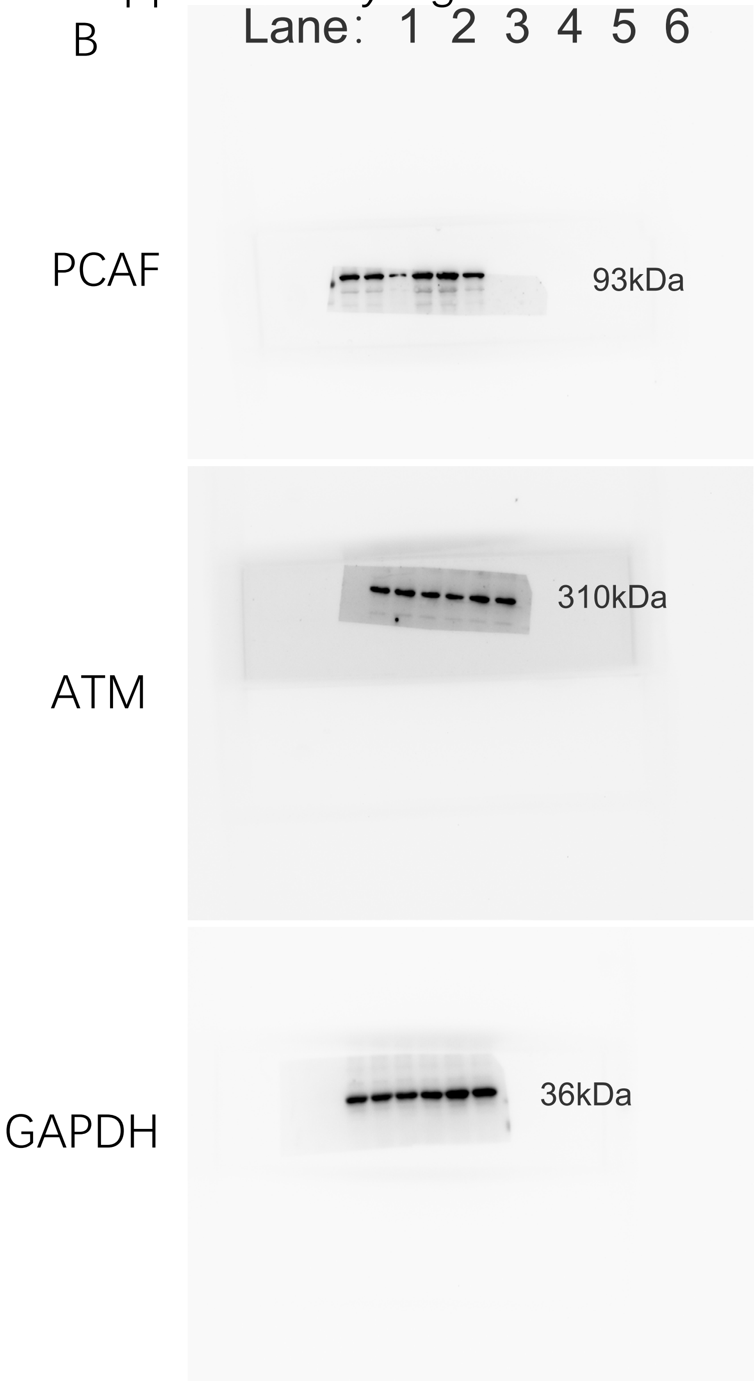

1: Control
2: Negative control
3: PCAF-KD
4: Control
5: Negative control
6: PCAF-KD
Line 1-3: U87R
Line 4-6: U251R

Supplementary Figure S9C

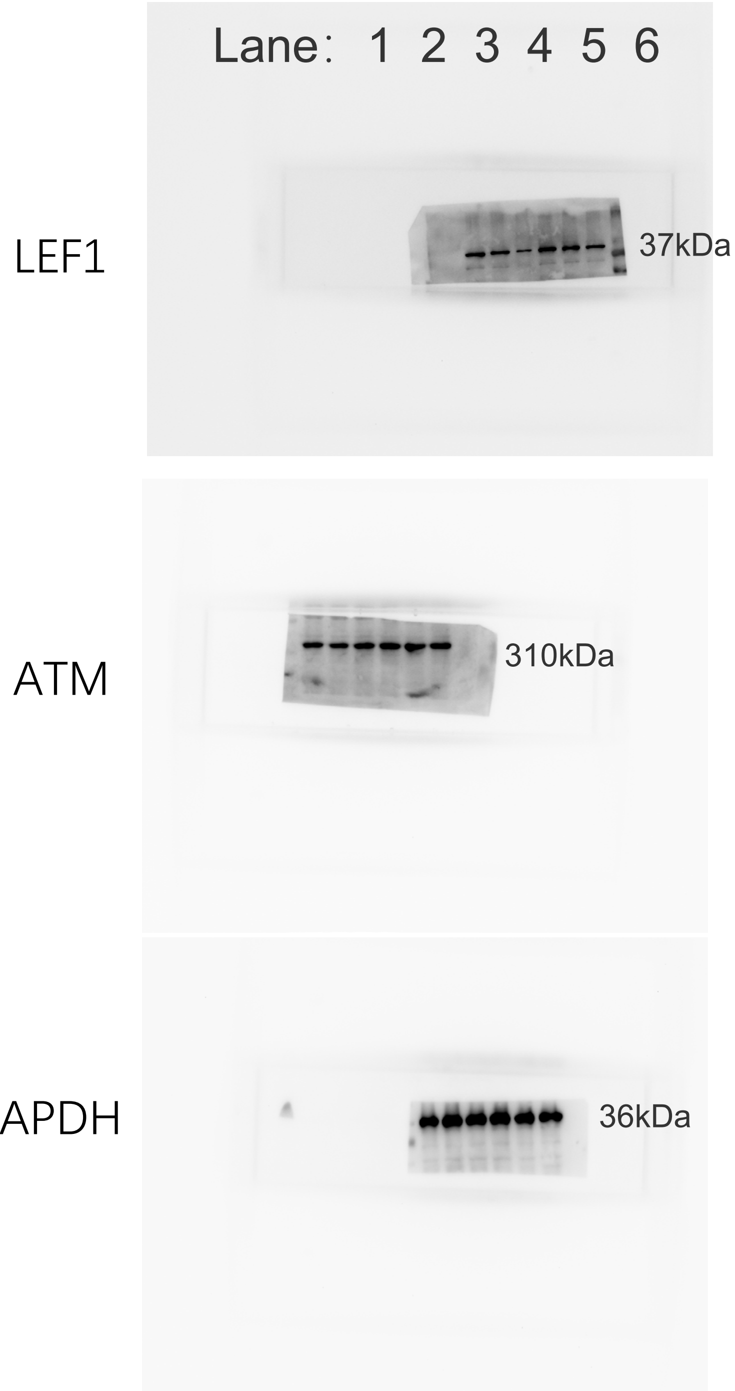

1: Control
2: Negative control
3: LEF1-KD
4: Control
5: Negative control
6: LEF1-KD
Line 1-3: U87R
Line 4-6: U251R

Supplementary Figure S9E

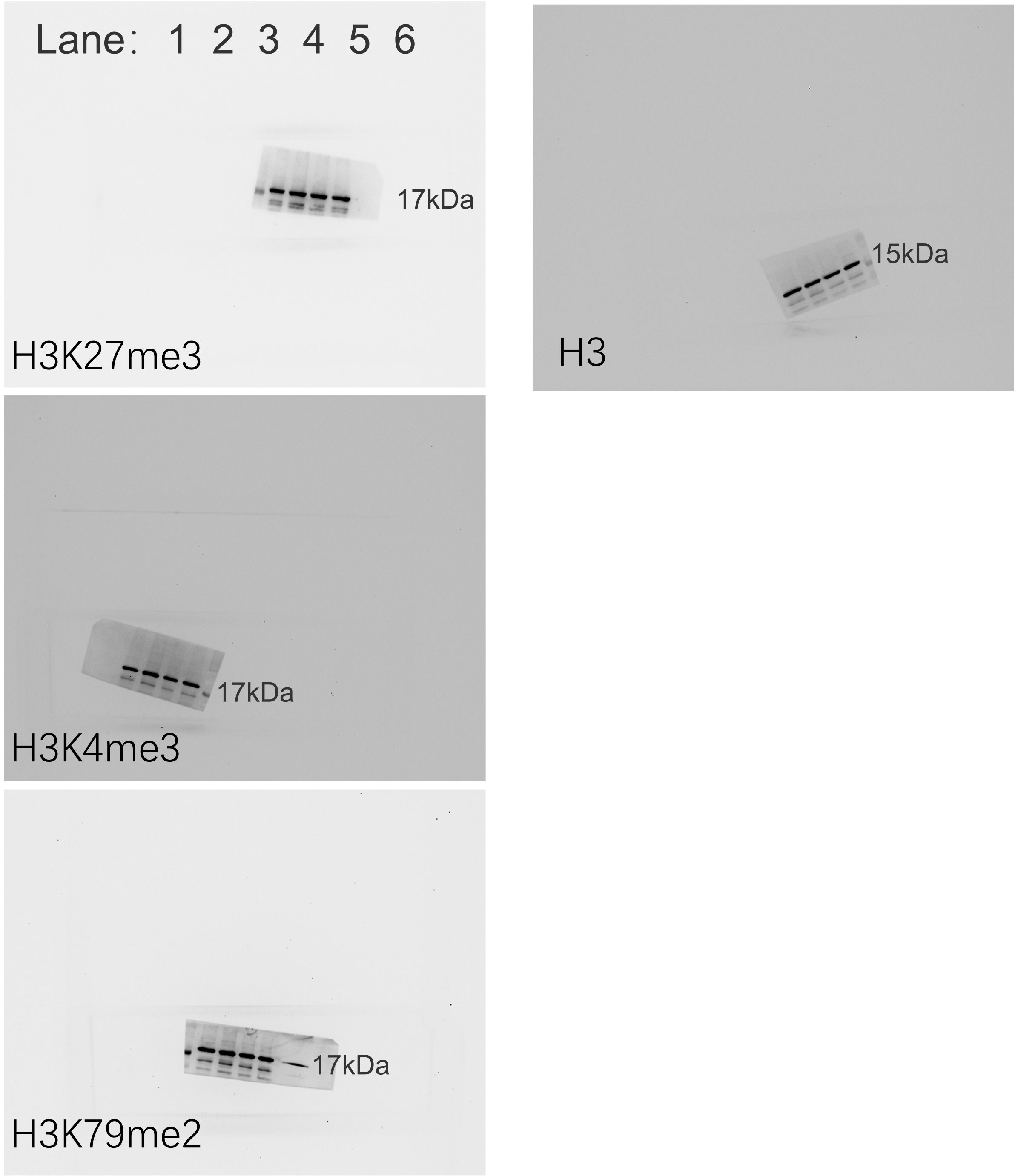

Supplement: Supplementary file 13 — Original Data of WB blots [file 41419_2022_4590_MOESM13_ESM.pdf]
